# Characterization and chemical reactivity of room-temperature-stable Mn^III^–alkylperoxo complexes†

**DOI:** 10.1039/d1sc01976g

**Published:** 2021-08-20

**Authors:** Adedamola A. Opalade, Joshua D. Parham, Victor W. Day, Timothy A. Jackson

**Affiliations:** The University of Kansas, Department of Chemistry, Center for Environmentally Beneficial Catalysis 1567 Irving Hill Road Lawrence KS 66045 USA taj@ku.edu +1-785-864-3968

## Abstract

While alkylperoxomanganese(iii) (Mn^III^–OOR) intermediates are proposed in the catalytic cycles of several manganese-dependent enzymes, their characterization has proven to be a challenge due to their inherent thermal instability. Fundamental understanding of the structural and electronic properties of these important intermediates is limited to a series of complexes with thiolate-containing N_4_S^−^ ligands. These well-characterized complexes are metastable yet unreactive in the direct oxidation of organic substrates. Because the stability and reactivity of Mn^III^–OOR complexes are likely to be highly dependent on their local coordination environment, we have generated two new Mn^III^–OOR complexes using a new amide-containing N_5_^−^ ligand. Using the 2-(bis((6-methylpyridin-2-yl)methyl)amino)-*N*-(quinolin-8-yl)acetamide (H^6Me^dpaq) ligand, we generated the [Mn^III^(OO^*t*^Bu)(^6Me^dpaq)]OTf and [Mn^III^(OOCm)(^6Me^dpaq)]OTf complexes through reaction of their Mn^II^ or Mn^III^ precursors with ^*t*^BuOOH and CmOOH, respectively. Both of the new Mn^III^–OOR complexes are stable at room-temperature (*t*_1/2_ = 5 and 8 days, respectively, at 298 K in CH_3_CN) and capable of reacting directly with phosphine substrates. The stability of these Mn^III^–OOR adducts render them amenable for detailed characterization, including by X-ray crystallography for [Mn^III^(OOCm)(^6Me^dpaq)]OTf. Thermal decomposition studies support a decay pathway of the Mn^III^–OOR complexes by O–O bond homolysis. In contrast, direct reaction of [Mn^III^(OOCm)(^6Me^dpaq)]^+^ with PPh_3_ provided evidence of heterolytic cleavage of the O–O bond. These studies reveal that both the stability and chemical reactivity of Mn^III^–OOR complexes can be tuned by the local coordination sphere.

## Abstract 2

A pair of room-temperature-stable Mn^III^–alkylperoxo complexes were characterized and shown to oxidize PPh_3_. Thermal decomposition studies provide evidence of both homolysis and heterolysis of the Mn^III^–alkylperoxo O–O bond.

## Graphical abstract

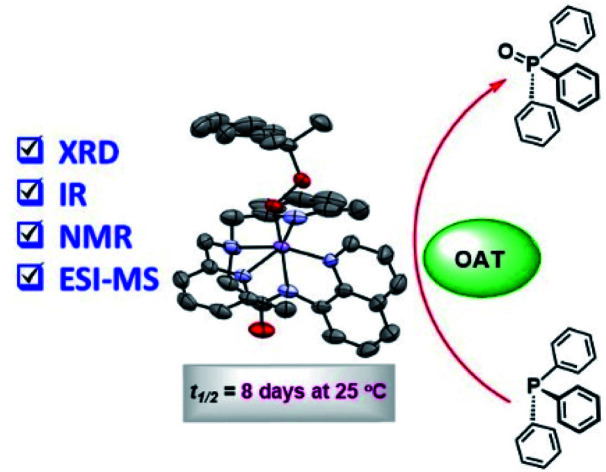

## Introduction

Metal–alkylperoxo adducts are essential species in industrial and biological oxidation reactions.^1–3^ For example, Co^III^–alkylperoxo adducts are proposed as intermediates in the industrial oxidation of cyclohexane to adipic acid.^1,4–7^ In the oxidation mechanism, homolytic cleavage of the O–O bond of a Co^III^–cyclohexylperoxo species leads to the production of cyclohexanol and cyclohexanone.^4,8^ Further radical-induced oxidation of cyclohexanone by C–C bond cleavage yields adipic acid.^4^ In biological systems, metal–alkylperoxo adducts are common intermediates in a variety of oxygenase enzymes, where they can be directly involved in substrate oxidation or precede the formation of high-valent metal–oxo species.^9,10^ Given the importance of metal–alkylperoxo species in such reactions, there are now many examples of synthetic Fe-,^11,12^ Co-,^1,4,5^ and Cu–alkylperoxo^3,13,14^ adducts, and these complexes are capable of oxidizing substrates such as 1,4-cyclohexadiene, 2-phenylpropionaldehyde, and triphenylphosphine.

**Fig. 1:**
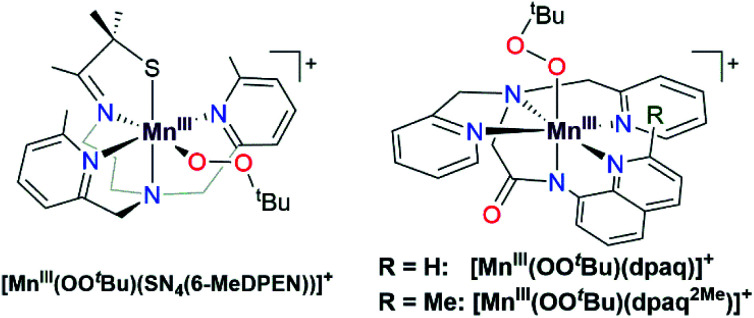
Schematic structures of [Mn^III^(OO^*t*^Bu)(SN_4_(6-MeDPEN))]^+^ (left) and [Mn^III^(OO^*t*^Bu)(dpaq^R^)]^+^ (right). The [Mn^III^(OO^*t*^Bu)(SN_4_(6-MeDPEN))]^+^ complex is a representative example of a Mn^III^–alkylperoxo adduct supported by an N_4_S^−^ ligand.

While such studies of synthetic model complexes have probed the properties and reactivity of many types of metal–alkylperoxo complexes, examples of Mn–alkylperoxo adducts are more limited, and there remain many open questions concerning the factors governing the decay and reactivity of these complexes. Kovacs and co-workers have performed pioneering investigations of Mn^III^–alkylperoxo adducts, including structural characterization of a family of complexes by X-ray crystallography.^15,16^ These studies employed pentadentate, thiolate-containing N_4_S^−^ ligands, which in the corresponding [Mn^III^(OOR)(N_4_S)]^+^ complexes placed the thiolate donor *cis* to the alkylperoxo ligand, with bulky quinolinyl or 6-methylpyridyl substituents *trans* to each other and *cis* to the alkylperoxo moiety (Fig. 1, left).^15,16^ The crystallographically observed Mn–N distances for the quinolinyl and 6-methylpyridyl donors range from 2.35 to 2.52 Å, which are quite long for Mn^III^–N interactions. Interestingly, these long Mn–N distances are correlated with the alkylperoxo O–O bond lengths, which vary from 1.43 to 1.47 Å.^16^ As shorter Mn–N distances gave longer O–O bonds, it was proposed that less Lewis acidic Mn^III^ centers yielded more activated Mn^III^–alkylperoxo adducts. By using variable-temperature kinetic studies, the O–O bond lengths for these Mn^III^–alkylperoxo complexes were in turn related to their thermal decay rates. Mn^III^–alkylperoxo adducts with longer O–O bonds decayed more rapidly, with lower Δ*H*^‡^ values and Δ*S*^‡^ values that were more negative.^16^ Because of the correlation between the Mn–N distances and the O–O bond lengths, these results suggest that activation of Mn^III^–alkylperoxo complexes can be controlled by the donor strength of groups *cis* to the alkylperoxo unit. Thermal decomposition studies and analysis of the decay products of the Mn^III^–cumylperoxo adduct supported a decay by homolytic cleavage of the alkylperoxo O–O bond.^16^ Very recently, Kovacs *et al.* reported a room-temperature stable Mn^III^–alkylperoxo complex supported by an alkoxide analogue of the N_4_S^−^ ligands.^17^ DFT computations for the [Mn^III^(OO^*t*^Bu)(N_4_O)]^+^ and [Mn^III^(OO^*t*^Bu)(N_4_S)]^+^ pair support the notion that the enhanced stability of the alkoxide-ligated complex arises from greater Lewis acidity of the Mn^III^ center.

While these studies provide structure-reactivity correlations with regards to the thermal decay pathway, the Mn^III^–alkylperoxo adducts of the N_4_S^−^ ligands failed to show any direct reaction towards a range of substrates.^16^ Product analysis of the reaction solutions provided evidence of the oxidation of a variety of substrates (*i.e.*, PEt_3_, TEMPOH, and cyclohexane carboxaldehyde) following the decay of the Mn^III^–alkylperoxo adducts, implying that a product of the decay pathway is a capable oxidant. In contrast, Mn^III^–hydroperoxo complexes supported by neutral, macrocyclic N_4_ ligands are known to react directly with aldehydes, sulfides, and hydrocarbons possessing weak C–H bonds.^18,19^ Given that metal–alkylperoxo adducts are often taken as analogues of metal–hydroxoperoxo species,^18–25^ the stark difference in reactivity between Mn^III^–alkylperoxo and Mn^III^–hydroperoxo adducts in substrate oxidation reactions is striking. The disparate reactivities of these complexes might reflect the differences in the properties of the supporting ligands employed (N_4_S^−^ for Mn^III^–alkylperoxo *versus* neutral N_4_ for Mn^III^–hydroperoxo). There is a clear need to understand better the role of non-thiolate-containing supporting ligands in influencing the properties and reactivity of Mn^III^–alkylperoxo complexes.

We previously generated a pair of Mn^III^–alkylperoxo complexes with ligands lacking thiolate ligation.^26^ These Mn^III^–alkylperoxo complexes were supported by the pentadentate dpaq and dpaq^2Me^ ligands, both of which feature strongly donating amide groups trans to the alkylperoxo moiety (Fig. 1, right; dpaq = 2-[bis(pyridin-2-ylmethyl)]amino-*N*-quinolin-8-yl-acetamidate, dpaq^2Me^ = 2-[bis(pyridin-2-ylmethyl)]amino-*N*-2-methyl-quinolin-8-yl-acetamidate).^26^ Both [Mn^III^(OO^*t*^Bu)(dpaq)]^+^ and [Mn^III^(OO^*t*^Bu)(dpaq^2Me^)]^+^ were unstable (*t*_1/2_ = 3200 and 3600 s, respectively, for 2 mM solution in CH_3_CN at −15 °C) but were characterized by electronic absorption, Mn K-edge X-ray absorption, and FT-IR spectroscopies.^26^ The observation of tBuOO˙ in EPR spectra of the complexes following their thermal decay provided support for a decay pathway involving Mn–O bond homolysis.^26^ While these data suggest differences in decay pathways for the thiolate- *versus* non-thiolate-ligated complexes, the large excess of ^*t*^BuOOH (*ca.* 100 equivalents) required to form the [Mn^III^(OO^*t*^Bu)(dpaq)]^+^ and [Mn^III^(OO^*t*^Bu)(dpaq^2Me^)]^+^ complexes made a complete analysis of their reactivity and decay pathways unfeasible. The large excess of ^*t*^BuOOH also complicated any investigations of substrate oxidation.

**Fig. 2:**
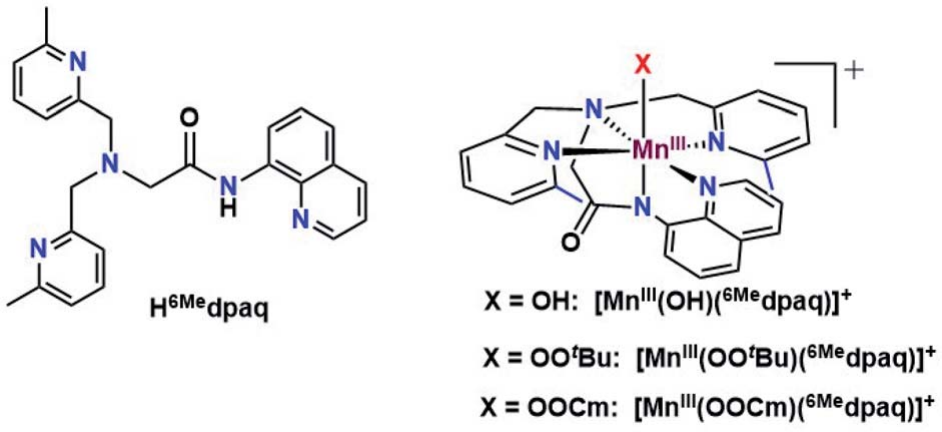
H^6Me^dpaq ligand (left), and [Mn^III^(OH)(^6Me^dpaq)]^+^, [Mn^III^(OO^*t*^Bu)(^6Me^dpaq)]^+^, and [Mn^III^(OOCm)(^6Me^dpaq)]^+^ complexes (right). The 6-Me-pyridyl substituents have been highlighted in blue in the structure of the metal complexes.

Given the limitations of the Mn^III^–alkylperoxo complexes of the dpaq and dpaq^2Me^ ligands, we sought to develop a derivative of the dpaq ligand that would better stabilize the Mn^III^–alkylperoxo adduct. Herein, we report Mn^III^–alkylperoxo adducts supported by ^6Me^dpaq (Fig. 2). This new ligand incorporates steric bulk at the 6 position of the pyridyl substituents in the equatorial plane. This choice was inspired by the higher stability of the Mn^III^–alkylperoxo complexes supported by N_4_S^−^ ligands with two bulky N-donor ligands *cis* to the alkylperoxo ligand (Fig. 1, left). X-ray crystallographic characterization of the Mn^III^–hydroxo adduct [Mn^III^(OH)(^6Me^dpaq)](OTf) reveals that the 6-methylpyridyl groups cause elongations in the Mn–N distance of 0.11 Å relative to [Mn^III^(OH)(dpaq)](OTf).^27^ The [Mn^III^(OH)(^6Me^dpaq)](OTf) complex reacts with stoichiometric amounts of ^*t*^BuOOH and CmOOH in CH_3_CN to generate the [Mn^III^(OO^*t*^Bu)(^6Me^dpaq)]^+^ and [Mn^III^(OOCm)(^6Me^dpaq)]^+^ complexes. These Mn^III^–alkylperoxo complexes are stable in solution at room temperature with half-lives of *ca.* 5 and 8 days, respectively. Structural characterization for [Mn^III^(OOCm)(^6Me^dpaq)]^+^ was obtained by X-ray crystallography. Despite their relatively high thermal stabilities, kinetic studies of [Mn^III^(OO^*t*^Bu)(^6Me^dpaq)]^+^ and [Mn^III^(OOCm)(^6Me^dpaq)]^+^ provide evidence for the direct reaction of these Mn^III^–alkylperoxo adducts with phosphines. These results show that the ligand sphere of Mn^III^–alkylperoxo adducts is critically important in governing their reactivity in substrate oxidation reactions.

## Results and analysis

### Formation and characterization of [Mn^II^(OH_2_)(^6Me^dpaq)](OTf) and [Mn^III^(OH)(^6Me^dpaq)](OTf)

**Fig. 3:**
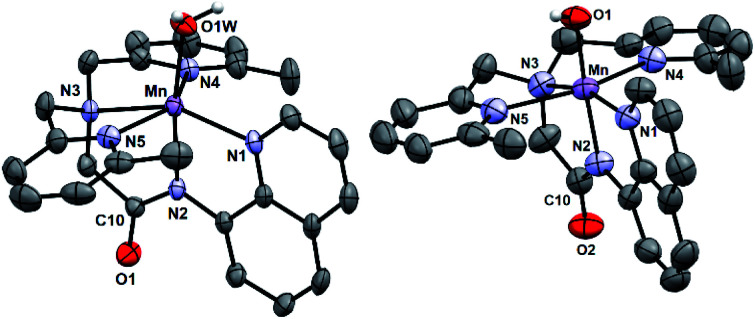
X-ray crystal structures of [Mn^II^(H_2_O)(^6Me^dpaq)]OTf and [Mn^III^(OH)(^6Me^dpaq)](OTf) showing 50% probability thermal ellipsoid. Triflate counter ion and non-aqua and non-hydroxo hydrogen atoms were removed for clarity.

[Mn^II^(H_2_O)(^6Me^dpaq)]OTf was prepared by the metalation of H^6Me^dpaq with Mn^II^(OTf)_2_. The X-ray crystal structure of [Mn^II^(H_2_O)(^6Me^dpaq)]OTf reveals a monomeric, six-coordinate Mn^II^ center coordinated by the pentadentate ^6Me^dpaq ligand and a water molecule (Fig. 3, left). Previous X-ray crystal structures for [Mn^II^(dpaq)](OTf)^27^ and [Mn^II^(dpaq^2Me^)](OTf)^28^ showed polymeric species, where the coordination site *trans* to the amide function was occupied by a carbonyl oxygen from a second [Mn^II^(dpaq)]^+^ (or [Mn^II^(dpaq^2Me^)]^+^) cation (Fig. S4†). The 6-Me-pyridyl functions in [Mn^II^(H_2_O)(^6Me^dpaq)]OTf give rise to elongated bonds (Mn–N4 and Mn–N5) when compared to those of [Mn^II^(dpaq)](OTf) and [Mn^II^(dpaq)^2Me^](OTf) (Tables 1 and S1†). The bonds involving the amide and amine functions (Mn–N2 and Mn–N3, respectively) of [Mn^II^(H_2_O)(^6Me^dpaq)]OTf are slightly contracted compared to the corresponding distances in [Mn^II^(dpaq)](OTf) and [Mn^II^(dpaq)^2Me^](OTf) (Table 1), which could reflect some compensation for the longer bond lengths with the 6-Me-pyridyl donors. In the solid-state structure, the aqua ligand of [Mn^II^(H_2_O)(^6Me^dpaq)]OTf hydrogen bonds with both an amide oxygen of a neighboring [Mn^II^(H_2_O)(^6Me^dpaq)]OTf molecule in the unit cell and with an oxygen of a triflate counter anion (H⋯O separations of 1.79(7) and 1.86(6) Å, respectively). Solution-phase characterization of [Mn^II^(H_2_O)(^6Me^dpaq)]OTf in CH_3_CN by EPR (Fig. S5†), ESI-MS (Fig. S6†), and Evans NMR (Fig. S7†) provide evidence that the mononuclear structure observed in the solid state is retained in solution.

**Table 1:** Manganese–ligand bond lengths (Å) and angles from X-ray crystal structures of [Mn^II^(OH_2_)(^6Me^dpaq)](OTf), [Mn^II^(dpaq)](OTf), [Mn^III^(OH)(^6Me^dpaq)](OTf), [Mn^III^(OH)(dpaq)](OTf), and [Mn^III^(OOCm)(^6Me^dpaq)](OTf)

Bond	[Mn^II^(OH_2_)(L)](OTf)	[Mn^II^(L)](OTf)	[Mn^III^(OH)(L)](OTf)		[Mn^III^(OOCm)(L)](OTf)
	L = ^6Me^dpaq	L = dpaq	L = ^6Me^dpaq	L = dpaq	L = ^6Me^dpaq
Mn–O1	2.108(3)	2.079(2)^a^	1.806(6)	1.806(13)	1.849(3)
Mn–N1	2.233(3)	2.214(3)	2.041(7)	2.072(14)	2.044(4)
Mn–N2	2.152(4)	2.191(3)	1.962(6)	1.975(14)	1.955(4)
Mn–N3	2.280(3)	2.314(3)	2.130(6)	2.173(14)	2.100(4)
Mn–N4	2.354(4)	2.244(3)	2.322(6)	2.260(14)	2.284(4)
Mn–N5	2.417(3)	2.286(3)	2.381(7)	2.216(15)	2.394(4)
O1–O2					1.466(4)
Mn–O1–O2					110.4(2)

a For [Mn^II^(dpaq)](OTf), the oxygen atom derives from a carbonyl unit of a separate [Mn^II^(dpaq)]^+^ cation.

[Mn^II^(H_2_O)(^6Me^dpaq)]OTf reacts very slowly with dioxygen. Electronic absorption data collected during the oxygenation of a 2.5 mM solution of [Mn^II^(H_2_O)(^6Me^dpaq)]OTf in CH_3_CN shows the appearance of a single feature at 510 nm, but this new chromophore is still forming even after 48 hours (Fig. S8,† left). This reactivity contrasts with that of [Mn^II^(dpaq)](OTf) and the majority of its derivatives, as these Mn^II^ complexes reacted with dioxygen with full conversion to Mn^III^ products over the course of several hours.^27–29^ Presumably, the elongation of the Mn–N4 and Mn–N5 bonds in [Mn^II^(H_2_O)(^6Me^dpaq)]OTf leads to a more electron-deficient Mn^II^ center with muted reactivity with dioxygen. In contrast, the reaction between [Mn^II^(H_2_O)(^6Me^dpaq)]OTf and 0.5 equiv. PhIO is rapid, resulting in the formation of a bronze colored solution with a single electronic absorption feature at *ca*. 510 nm (Fig. S8,† right), which can be attributed to [Mn^III^(OH)(^6Me^dpaq)](OTf) (*vide infra*).

X-ray diffraction studies of crystals obtained from this dark orange solution establish the oxidation product as [Mn^III^(OH)(^6Me^dpaq)](OTf) (Fig. 3, right). In this complex, a six-coordinate Mn^III^ center is in a distorted octahedral geometry with the hydroxo ligand *trans* to the amide function. This coordination mode is identical to that observed in Mn^III^–hydroxo complexes of dpaq and its derivatives.^27–29^ The Mn–OH distance of 1.806(6) Å is within error of that observed for [Mn^III^(OH)(dpaq)](OTf) (1.806(13); see Table 1)^27^ and is on the low end of the range of Mn–OH bond lengths reported for other Mn^III^–hydroxo complexes (1.81–1.86 Å).^28,30–38^ Further comparisons of the X-ray structure of [Mn^III^(OH)(^6Me^dpaq)](OTf) with those of related Mn^III^–hydroxo species reveals that the 6-Me-pyridyl groups give rise to substantial bond elongations. Specifically, the Mn–N4 and Mn–N5 distances of [Mn^III^(OH)(^6Me^dpaq)](OTf) are 2.322(6) and 2.381(7) Å, while the corresponding distances in [Mn^III^(OH)(dpaq)](OTf) are 2.260(14) and 2.216(15) Å (Table 1).^27^ Mn^III^ complexes of the N_4_S^−^ class of ligands (Fig. 1) with 6-Me-pyridyl or quinolinyl substituents in the equatorial field had Mn–N distances ranging from 2.352–2.581 Å.^16,39^ The 6-Me-pyridyl Mn–N distances of [Mn^III^(OH)(^6Me^dpaq)](OTf) are thus on the short end of this range. For [Mn^III^(OH)(^6Me^dpaq)](OTf), the longer Mn–N4 and Mn–N5 distances are accompanied by modest contractions of Mn–N1, Mn–N2, and Mn–N3 by 0.031, 0.013, and 0.043 Å, respectively, relative to [Mn^III^(OH)(dpaq)](OTf) (Table 1). The extended X-ray structure of [Mn^III^(OH)(^6Me^dpaq)](OTf) reveals a hydrogen bond between the hydroxo ligand and the amide oxygen of a neighboring [Mn^III^(OH)(^6Me^dpaq)]^+^ cation (H⋯O distance of 2.006 Å, see Fig. S11†). This kind of interaction was also observed in the crystal structure of [Mn^III^(OH)(^2Me^dpaq)](OTf) (H⋯O distance of 1.982 Å, see Fig. S11†).^28^ A free triflate ion is also present in the asymmetric unit of [Mn^III^(OH)(^6Me^dpaq)](OTf), though there is no interaction with the Mn center (closest Mn–O distance of *ca.* 7.5 Å).

**Fig. 4:**
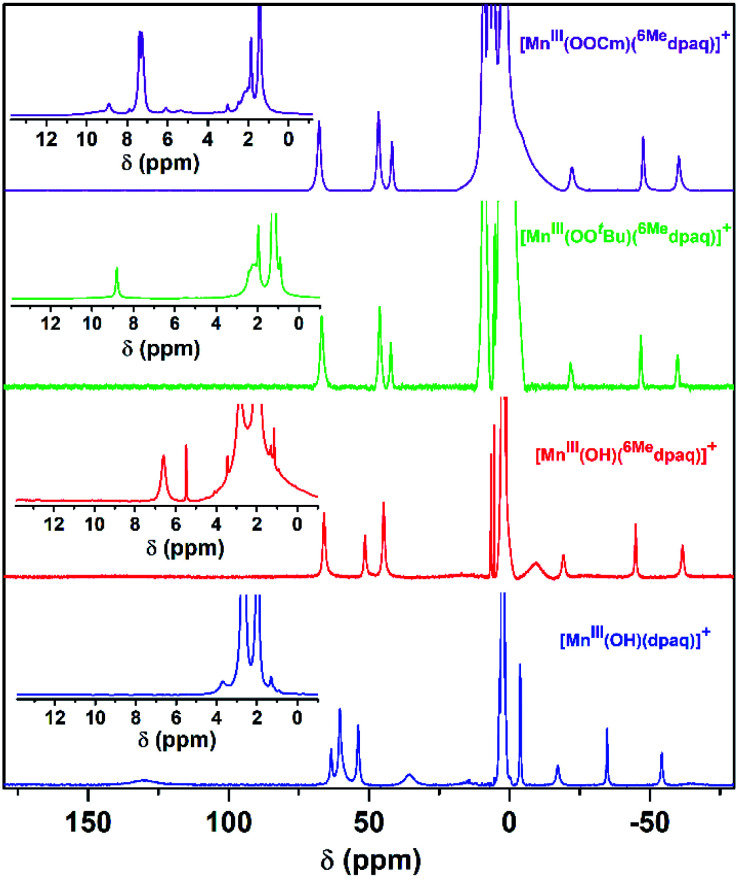
^1^H NMR spectra of solutions of 20 mM [Mn^III^(OH)(^6Me^dpaq)]^+^ (red), 15 mM [Mn^III^(OO^*t*^Bu)(^6Me^dpaq)]^+^ (green), 15 mM [Mn^III^(OOCm)(^6Me^dpaq)]^+^ (purple), and 15 mM [Mn^III^(OH)(dpaq)]^+^ (blue) with 45 μL added D_2_O. All samples were prepared in CD_3_CN at 298 K. The Mn^III^–alkylperoxo complexes were prepared by treating [Mn^III^(OH)(^6Me^dpaq)]^+^ with 1 equiv. ^*t*^BuOOH (green) or CmOOH (purple). Inset: expanded view of the 0 to 13 ppm region.

Previous investigations of [Mn^III^(OH)(dpaq)](OTf) and a subset of its derivatives have shown that dissolution of the salts of these Mn^III^–hydroxo complexes in dried CH_3_CN leads to the formation of an equilibrium mixture of Mn^III^–hydroxo and (μ-oxo)dimanganese(III,III) complexes that can both be detected by ^1^H NMR spectroscopy.^29,40^ The ^1^H NMR spectrum of [Mn^III^(OH)(^6Me^dpaq)](OTf) in CD_3_CN at 298 K exhibits seven hyperfine-shifted peaks that lie well outside 0–20 ppm (the diamagnetic region), as well as two well-resolved peaks in the 0–10 ppm region (Fig. 4, red trace). The lack of a large number of peaks in the diamagnetic region suggests that dissolution of [Mn^III^(OH)(^6Me^dpaq)](OTf) in CD_3_CN does not result in the formation of (μ-oxo)dimanganese(iii,iii) species. The chemical shifts for the ^1^H NMR signals of [Mn^III^(OH)(^6Me^dpaq)]^+^ are quite similar to those of [Mn^III^(OH)(dpaq)]^+^ (Fig. 4 and Table 2).^29,40^ The four most upfield-shifted peaks in the ^1^H NMR spectrum of [Mn^III^(OH)(dpaq)]^+^ were assigned to protons from the quinolinyl moiety.^40^ The upfield region of the ^1^H NMR spectrum of [Mn^III^(OH)(^6Me^dpaq)]^+^ shows three sharp peaks at −19.3, −45.0, and −61.6 ppm that resemble the peaks of [Mn^III^(OH)(dpaq)]^+^ at −15.5, −33.7, −53.8 ppm (Fig. 4). The ^1^H NMR spectrum of [Mn^III^(OH)(^6Me^dpaq)]^+^ lacks a broad, highly upfield-shifted peak analogous to the weak, broad −63.4 ppm signal of [Mn^III^(OH)(dpaq)]^+^, but the breadth of this signal renders it difficult to resolve. The overall similarities between the upfield regions of the ^1^H NMR spectra of [Mn^III^(OH)(^6Me^dpaq)]^+^ and [Mn^III^(OH)(dpaq)]^+^ are expected given the lack of changes to the quinolinyl group in the former complex. The downfield ^1^H NMR signals of [Mn^III^(OH)(dpaq)]^+^ and the resonance at −4.6 ppm were attributed to pyridyl protons.^40^ Consequently, the larger relative perturbations in the downfield regions of the ^1^H NMR spectra of [Mn^III^(OH)(dpaq)]^+^ and [Mn^III^(OH)(^6Me^dpaq)]^+^ can be rationalized by changes in chemical shifts of pyridyl protons. The peak at 130.5 ppm for [Mn^III^(OH)(dpaq)]^+^ was assigned to the α-H of the pyridine substituent.^40^ The lack of a corresponding peak in the ^1^H NMR spectrum of [Mn^III^(OH)(^6Me^dpaq)]^+^ is consistent with the functionalization of the pyridyl functions in the 6-position. The ^1^H NMR spectrum of [Mn^III^(OH)(^6Me^dpaq)]^+^ contains a broad, upfield peak at −9.5 ppm, lacking in the ^1^H NMR spectrum of [Mn^III^(OH)(dpaq)]^+^, that we attribute to protons of the 6-methyl-substituents. Overall, the ^1^H NMR spectra of [Mn^III^(OH)(^6Me^dpaq)]^+^ and [Mn^III^(OH)(dpaq)]^+^ are quite similar, and the observed differences can be rationalized in terms of the presence of the 6-Me-pyridyl groups in the former complex.

**Table 2:** ^1^H NMR chemical shifts (ppm) for [Mn^III^(OH)(^6Me^dpaq)]^+^, [Mn^III^(OO^*t*^Bu)(^6Me^dpaq)]^+^, [Mn^III^(OOCm)(^6Me^dpaq)]^+^, and [Mn^III^(OH)(dpaq)]^+^ in CD_3_CN at 298 K^a^

[Mn^III^(OH)(^6Me^dpaq)]^+^	[Mn^III^(OO^*t*^Bu)(^6Me^dpaq)]^+^	[Mn^III^(OOCm)(^6Me^dpaq)]^+^	[Mn^III^(OH)(dpaq)]^+^
			130.5 (H-py)
66.0	66.8	67.8	62.7 (H-qn)
51.4	46.1	46.7	60.9 (H-py)
44.8	42.2	41.8	54.3 (H-py)
			40.5
8.9	9.2	8.9, 7.38, 7.26	
5.5		6.05, 5.3	
−9.6			−4.6 (H-py)
−19.3	−22	−22.3	−15.5 (H-qn)
			−33.7 (H-qn)
−45	−46.9	−47.6	−53.8 (H-qn)
−61.6	−60	−60.3	−63.4 (H-qn)

a Data and assignments (in parentheses) for [Mn^III^(OH)(dpaq)]^+^ in CD_3_CN with 880 equivalents of D_2_O from ref. 40 (py = pyridine, qn = quinoline).

### Formation of [Mn^III^(OO^*t*^Bu)(^6Me^dpaq)]^+^ and [Mn^III^(OOCm)(^6Me^dpaq)]^+^

**Fig. 5:**
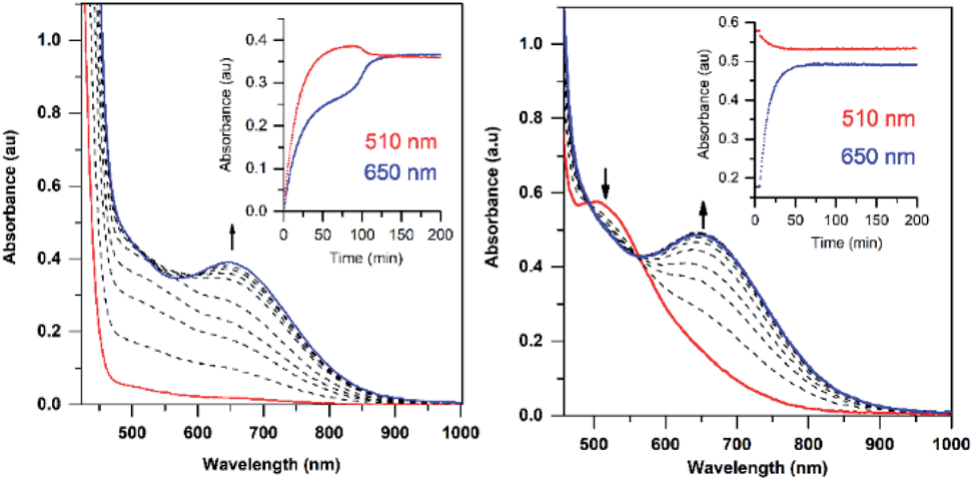
Left: electronic absorption spectra showing the formation of the green 1.5 mM [Mn^III^(OO^*t*^Bu)(^6Me^dpaq)]^+^ species (blue trace) from the oxidation of [Mn^II^(H_2_O)(^6Me^dpaq)]OTf (red trace) with 1.5 equiv. ^*t*^BuOOH. Right: electronic absorption spectra showing the formation of 2.0 mM [Mn^III^(OO^*t*^Bu)(^6Me^dpaq)]^+^ from the reaction of [Mn^III^(OH)(^6Me^dpaq)]^+^ (red trace) with 1.0 equiv. ^*t*^BuOOH (blue trace is the final spectrum). Time courses for each reaction are shown in the insets.

The addition of a slight excess of ^*t*^BuOOH to [Mn^II^(H_2_O)(^6Me^dpaq)]OTf in CH_3_CN at 298 K results in the formation of a green chromophore with a prominent electronic absorption band at 650 nm (*ε* = 240 M^−1^ cm^−1^) and a shoulder near 500 nm (*ε* = 263 M^−1^ cm^−1^; see Fig. 5, left). (Extinction coefficients were determined assuming full conversion under these conditions.) Maximal formation of the green chromophore from [Mn^II^(H_2_O)(^6Me^dpaq)]OTf requires 1.5 equiv. ^*t*^BuOOH (Fig. S12†). A similar reaction is observed upon the addition of CmOOH to [Mn^II^(H_2_O)(^6Me^dpaq)]OTf (Fig. S13†). Spectroscopic data provided below and in the ESI (Fig. S14–S16†) support the formulation of these green chromophores as [Mn^III^(OO^*t*^Bu)(^6Me^dpaq)]^+^ and [Mn^III^(OOCm)(^6Me^dpaq)]^+^. The half-lives of the 2 mM solutions of the [Mn^III^(OO^*t*^Bu)(^6Me^dpaq)]^+^ and [Mn^III^(OOCm)(^6Me^dpaq)]^+^ are *ca.* 5 and 8 days, respectively, in CH_3_CN at 298 K.

**Scheme 1:**
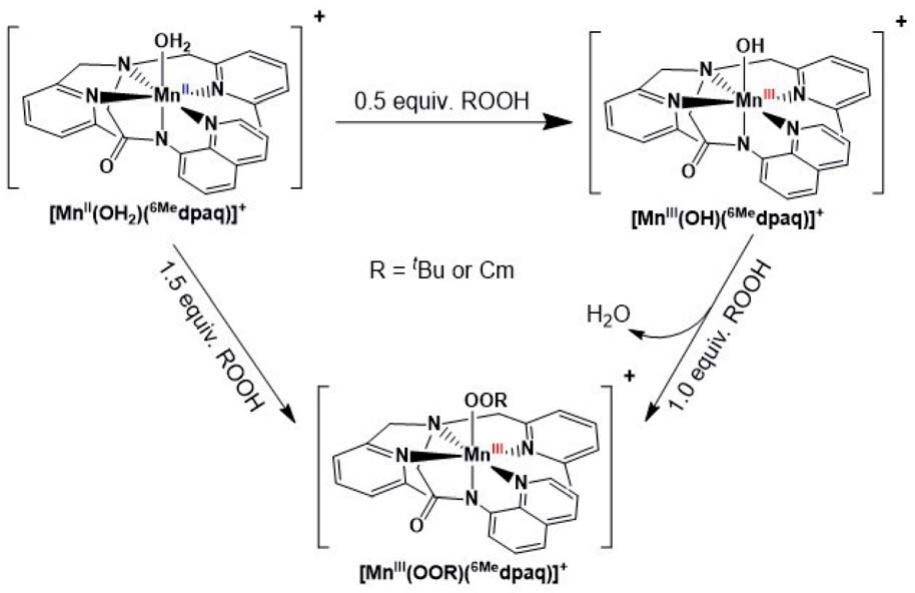
Synthetic route for the preparation of [Mn^III^(OOR)(^6Me^dpaq)]^+^.

The time course for the formation of [Mn^III^(OO^*t*^Bu)(^6Me^dpaq)]^+^ from [Mn^II^(H_2_O)(^6Me^dpaq)]^+^ shows an initial rise in absorbance intensity at 510 nm that maximizes near *ca*. 60 minutes and then drops and levels by 120 minutes. In contrast, the absorbance intensity at 650 nm shows a steep rise from 0 to 40 minutes, grows more slowly from 40–100 minutes, and then rises quickly and levels by 120 minutes (Fig. 5, left inset). On the basis of this reaction profile, we propose the formation of the Mn^III^–hydroxo adduct [Mn^III^(OH)(^6Me^dpaq)]^+^ as an intermediate in this reaction, as this species shows an absorption maximum at 510 nm. This proposal is consistent with the 1.5 : 1 ^*t*^BuOOH : Mn^II^ stoichiometry, where initial oxidation of [Mn^II^(H_2_O)(^6Me^dpaq)]OTf to [Mn^III^(OH)(^6Me^dpaq)]^+^ consumes 0.5 equiv. ^*t*^BuOOH, and the remaining 1.0 equiv. ^*t*^BuOOH converts [Mn^III^(OH)(^6Me^dpaq)]^+^ to [Mn^III^(OO^*t*^Bu)(^6Me^dpaq)]^+^ by a ligand substitution reaction that yields water as a co-product (Scheme 1). To test this mechanism, we added 1.0 equiv. ^*t*^BuOOH to [Mn^III^(OH)(^6Me^dpaq)]^+^ in CH_3_CN at 298 K and observed the formation of [Mn^III^(OO^*t*^Bu)(^6Me^dpaq)]^+^ in maximal yield (Fig. 5, right). In this case, the conversion is accompanied with isosbestic points at 505 and 555 nm, indicating the lack of an accumulating intermediate. The formation of a Mn^III^–hydroxo intermediate during the reaction of ^*t*^BuOOH with [Mn^II^(dpaq)](OTf) and [Mn^II^(dpaq^2Me^)](OTf) was observed previously.^26^

### Structural properties of [Mn^III^(OOCm)(^6Me^dpaq)]^+^

**Fig. 6:**
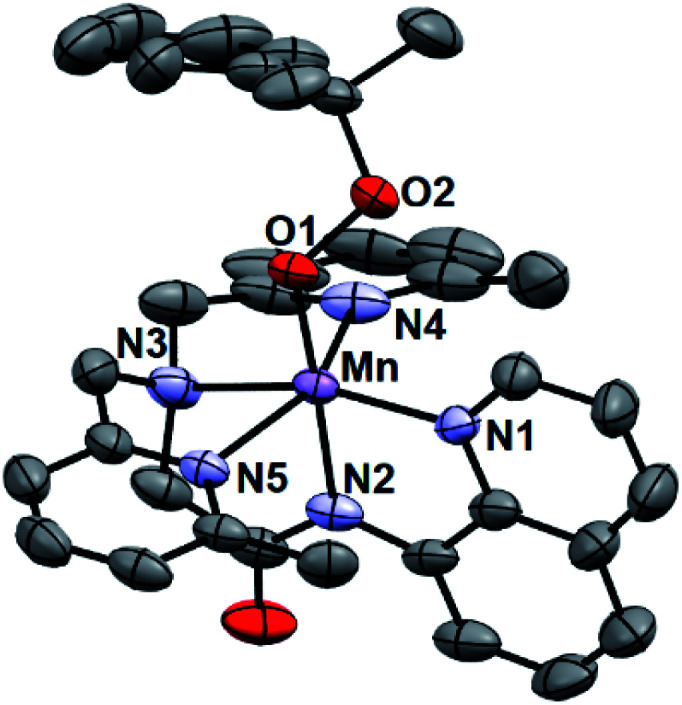
ORTEP diagram of cationic portion of [Mn^III^(OOCm)(^6Me^dpaq)](OTf) showing 50% probability thermal ellipsoids. Hydrogen atoms of the ^6Me^dpaq ligand, solvent of crystallization, and the triflate counterion were omitted for clarity.

While all attempts at obtaining crystalline material for [Mn^III^(OO^*t*^Bu)(^6Me^dpaq)](OTf) were unsuccessful, we were able to obtain diffraction-quality crystals for [Mn^III^(OOCm)(^6Me^dpaq)](OTf), which confirmed the formulation of this complex (Fig. 6). The cumylperoxo ligand of [Mn^III^(OOCm)(^6Me^dpaq)](OTf) is bound *trans* to the amide nitrogen (N2–Mn–O1 angle of 176.6°), occupying the position of the hydroxo ligand in [Mn^III^(OH)(^6Me^dpaq)](OTf). The Mn–O1 bond length for [Mn^III^(OOCm)(^6Me^dpaq)](OTf) is longer than the Mn–O1 bond in the Mn^III^–hydroxo analogue (1.849(3) and 1.806(6) Å, respectively), but similar to crystallographic Mn–OOCm distances for the [Mn^III^(OOCm)(N_4_S)]^+^ complexes (1.848(4) and 1.84(1) Å).^15,16^ The O1–O2 bond is oriented such that the projection of this bond onto the equatorial plane bisects the N1–Mn–N4 bond angle. The aryl ring of the cumyl moiety is parallel to the plane of the pyridines coordinated to the Mn^III^ center, as opposed to the out-of-plane orientation observed in the [Mn^III^(S^Me2^N_4_(6-Me-DPEN))(OOCm)](BPh_4_) complex.^16^ This orientation of the cumyl moiety is stabilized by π–CH interactions between the aryl ring and a methylene group of the ^6Me^dpaq ligand, as evidenced by short H⋯C contacts of *ca.* 2.8 Å. The O1–O2 bond length of 1.466(4) Å for [Mn^III^(OOCm)(^6Me^dpaq)](OTf) is consistent with that expected for an alkylperoxo moiety^4,5,41,42^ and is within the range of values observed for the [Mn^III^(OOCm)(N_4_S)]^+^ complexes (1.457(5) and 1.51(2) Å).^15,16^ The nitrogen atoms of the 6-Me-pyridyl groups (N4 and N5 of [Mn^III^(OOCm)(^6Me^dpaq)](OTf)) are 2.284(4) and 2.394(4) Å, respectively, from the Mn center.

### Spectroscopic properties of [Mn^III^(OO^*t*^Bu)(^6Me^dpaq)]^+^ and [Mn^III^(OOCm)(^6Me^dpaq)]^+^

**Fig. 7:**
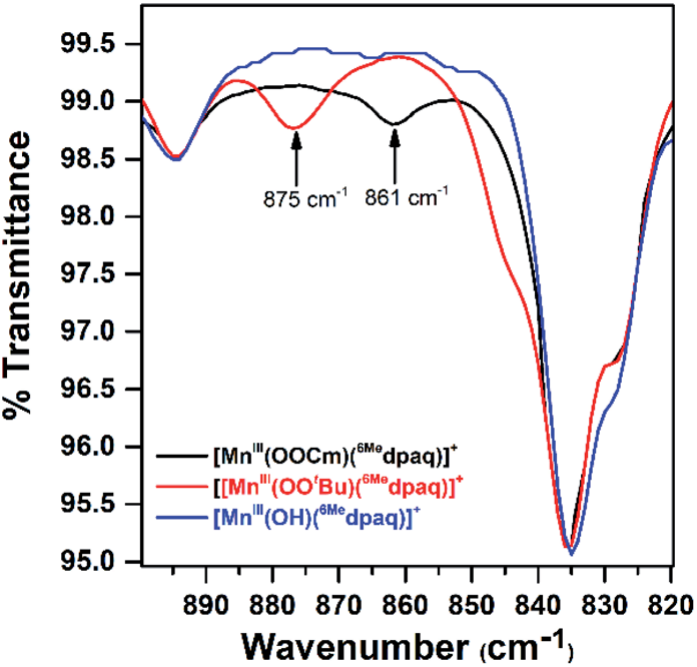
Solution FT-IR spectra obtained following the reactions of 2 mM solutions of [Mn^III^(OH)(^6Me^dpaq)]^+^ in CH_3_CN and with 1.0 equiv. ^*t*^BuOOH (red trace) and 1.0 equiv. CmOOH (black trace). The FT-IR spectrum of [Mn^III^(OH)(^6Me^dpaq)]^+^ (blue trace) is shown for comparison.

Although we were unable to obtain crystallographic information concerning [Mn^III^(OO^*t*^Bu)(^6Me^dpaq)]^+^, the ^1^H NMR spectrum of this species in CD_3_CN is essentially identical to that of [Mn^III^(OOCm)(^6Me^dpaq)]^+^ (Fig. 4 and Table 2). Each spectrum shows six peaks outside the diamagnetic region, three downfield and three upfield. The only notable difference between the spectra of [Mn^III^(OO^*t*^Bu)(^6Me^dpaq)]^+^ and [Mn^III^(OOCm)(^6Me^dpaq)]^+^ are the number of peaks from 10–5 ppm (Table 2). While [Mn^III^(OO^*t*^Bu)(^6Me^dpaq)]^+^ shows a single prominent peak at 9.2 ppm, [Mn^III^(OOCm)(^6Me^dpaq)]^+^ shows five peaks in this region. We tentatively attributed these peaks to protons from the *t*-butyl and cumyl moieties, respectively. The ^1^H NMR spectra of [Mn^III^(OOCm)(^6Me^dpaq)]^+^ and [Mn^III^(OO^*t*^Bu)(^6Me^dpaq)]^+^ are also very similar to that of [Mn^III^(OH)(^6Me^dpaq)]^+^ (Fig. 4 and Table 2), which is consistent with the same binding mode of the ^6Me^dpaq ligand in each of these complexes. In particular, the three upfield peaks of the Mn^III^–alkylperoxo adducts at *ca.* −22, −47, and −60 ppm have chemical shifts very similar to the upfield resonances observed for [Mn^III^(OH)(^6Me^dpaq)]^+^ (−19.3, −45, and −61.6 ppm; see Table 2). Similarly, the downfield peaks at *ca.* 67, 46, and 42 ppm in the ^1^H NMR spectrum of the Mn^III^–alkylperoxo adducts show only slight deviations from the corresponding resonances of [Mn^III^(OH)(^6Me^dpaq)]^+^ (66.0, 51.4, and 44.8 ppm; see Table 2). The solution FT-IR spectra of [Mn^III^(OO^*t*^Bu)(^6Me^dpaq)]^+^ and [Mn^III^(OOCm)(^6Me^dpaq)]^+^ show features at 875 cm^−1^ and 861 cm^−1^, respectively, that are absent in the FT-IR spectrum of [Mn^III^(OH)(^6Me^dpaq)]^+^ (Fig. 7). These features have energies similar to those of O–O vibrations reported for Mn^III^–alkylperoxo complexes (872–895 cm^−1^; Table S2†).^15,16,26^ To evaluate this assignment, we prepared [Mn^III^(^18^O^18^O^*t*^Bu)(^6Me^dpaq)]^+^, which was confirmed by ESI-MS experiments (Fig. S17†). In the IR spectrum of the ^18^O isotopologue, the 875 cm^−1^ feature found in the spectrum of [Mn^III^(^16^O^16^O^*t*^Bu)(^6Me^dpaq)]^+^ has disappeared, and we observe two new features at 854 cm^−1^ and 818 cm^−1^ (Fig. S18†). The lack of the 875 cm^−1^ band supports its assignment as the O–O vibration. The appearance of two new peaks suggests that the O–O vibration is coupled with other vibrations. The IR spectrum of ^18^O-labeled [Mn^III^(OO^*t*^Bu)(dpaq)]^+^ also showed two new features relative to the ^16^O sample.^26^ A normal coordinate analysis of high-spin Fe^III^–alkylperoxo adducts revealed coupling of O–O and *t*-butyl C–C vibrations that gave rise to unusual isotope shift patterns,^43^ and vibrational coupling has also been observed for Fe^III^–hydroperoxo adducts.^24,44^ Vibrational coupling could explain the multiple peaks observed in the IR spectrum of [Mn^III^(^18^O^18^O^*t*^Bu)(^6Me^dpaq)]^+^ and account for deviations in isotope shifts from Hooke's law, as coupled modes would not follow a simple harmonic oscillator model. Under this proposal, one might expect to observe multiple isotope-sensitive peaks in the IR spectrum of [Mn^III^(^16^O^16^O^*t*^Bu)(^6Me^dpaq)]^+^. However, such peaks could easily be obscured by the solvent-derived vibrations at *ca.* 895 cm^−1^ and from *ca.* 845–820 cm^−1^ (Fig. 7).

EPR analysis of frozen 5 mM CH_3_CN solutions of [Mn^III^(OO^*t*^Bu)(^6Me^dpaq)]^+^ and [Mn^III^(OOCm)(^6Me^dpaq)]^+^ collected at 10 K showed no signals in either perpendicular- or parallel-mode (Fig. S19†). This observation is consistent with the lack of X-band EPR signals for many Mn^III^ complexes due to moderate to large zero-field splitting relative to the microwave energy.^45–49^ The magnetic moments of these complexes (4.8*μ*_B_) are consistent with *S* = 2 Mn^III^ centers (Fig. S15 and S16†). Additional ESI-MS data and solution-phase magnetic moments further support the formulations for these Mn^III^–alkylperoxo complexes (Fig. S14†).

### Thermal decay pathways of [Mn^III^(OO^*t*^Bu)(^6Me^dpaq)]^+^ and [Mn^III^(OOCm)(^6Me^dpaq)]^+^

**Fig. 8:**
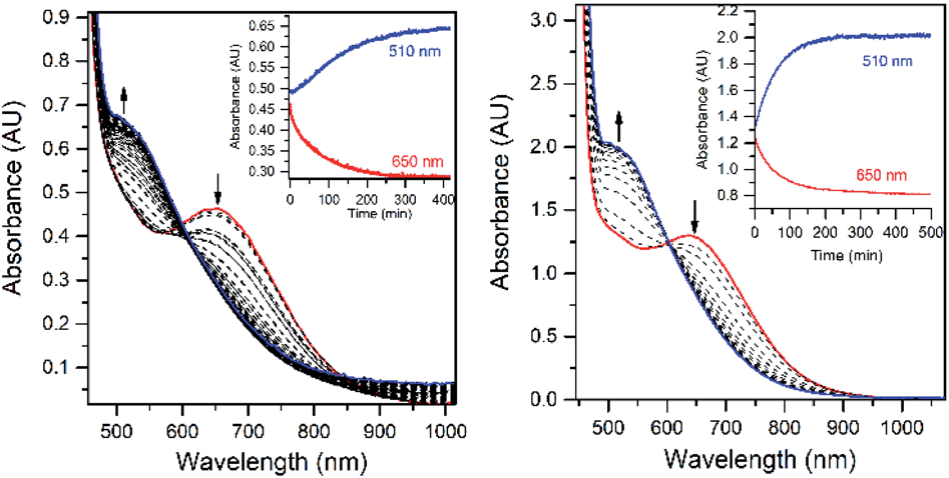
Electronic absorption spectra showing the decay of anaerobic CH_3_CN solutions of 2 mM [Mn^III^(OO^*t*^Bu)(^6Me^dpaq)]^+^ and 6 mM [Mn^III^(OOCm)(^6Me^dpaq)]^+^ (red traces) at 323 K to give [Mn^III^(OH)(^6Me^dpaq)]^+^ (blue traces). The inset shows the change in absorbance at 650 and 510 nm over the course of the decay reaction.

The thermal decay of [Mn^III^(OO^*t*^Bu)(^6Me^dpaq)]^+^ and [Mn^III^(OOCm)(^6Me^dpaq)]^+^ in CH_3_CN at 323 K under anaerobic conditions was monitored by electronic absorption spectroscopy. Each decay reaction progressed with the disappearance of the 650 nm feature associated with the Mn^III^–alkylperoxo adduct and the appearance of a feature at 510 nm (Fig. 8). The feature at 510 nm, along with ESI-MS analyses of the product solutions (Fig. S20†), marks [Mn^III^(OH)(^6Me^dpaq)]^+^ as the major product of these decay reactions (99 and 92% formation from [Mn^III^(OOCm)(^6Me^dpaq)]^+^ and [Mn^III^(OO^*t*^Bu)(^6Me^dpaq)]^+^, respectively, on the basis of the extinction coefficient of the Mn^III^–hydroxo complex). For [Mn^III^(OOCm)(^6Me^dpaq)]^+^, the decay proceeds with isosbestic behavior, and the respective decay and formation rates of the 650 and 510 nm electronic absorption signals are identical (Fig. 8, right; *k*_obs_ = 0.016 min^−1^ for both processes). In contrast, the thermal decay for [Mn^III^(OO^*t*^Bu)(^6Me^dpaq)]^+^ is not isosbestic, and the rate of formation of the 510 nm chromophore lags behind the decay of the 650 nm band (Fig. 8, left). We tentatively attribute this difference to the higher purity of [Mn^III^(OOCm)(^6Me^dpaq)]^+^ used in these experiments, as this complex was obtained as a recrystallized solid. In support, thermal decay studies of crude [Mn^III^(OOCm)(^6Me^dpaq)]^+^ also failed to show isosbestic behavior (Fig. S22†). Additional product analysis following the thermal decay of 18 mM [Mn^III^(OOCm)(^6Me^dpaq)]^+^ in CH_3_CN at 323 K revealed 61.3 ± 0.1% 2-phenyl-2-propanol and 25.7 ± 0.1% acetophenone formed relative to the initial [Mn^III^(OOCm)(^6Me^dpaq)]^+^ concentration. (The organic products from the thermal decay of [Mn^III^(OO^*t*^Bu)(^6Me^dpaq)]^+^ were not quantified, because the volatility of acetone, a potential product, renders quantification unreliable.)

When the decay of 6 mM [Mn^III^(OOCm)(^6Me^dpaq)]^+^ was performed in CD_3_CN, we observed 50 ± 0.3% 2-phenyl-2-propanol and 40 ± 0.3% acetophenone relative to the initial [Mn^III^(OOCm)(^6Me^dpaq)]^+^, a marked change in the product distribution. In addition, when [Mn^III^(OOCm)(^6Me^dpaq)]^+^ was allowed to decay in benzonitrile (PhCN; see Fig. S23†), we observed 30 ± 1.1% 2-phenyl-2-propanol and 70 ± 1.1% acetophenone. The implications of these results with respect to the decay mechanism are explored in the Discussion section (*vide infra*).

**Fig. 9:**
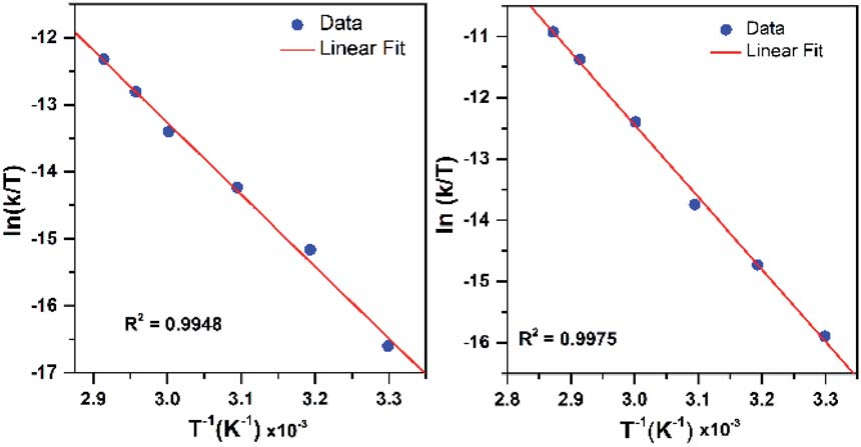
Eyring plot from variable-temperature thermal decay kinetics of [Mn^III^(OO^*t*^Bu)(^6Me^dpaq)]^+^ (left) and [Mn^III^(OOCm)(^6Me^dpaq)]^+^ (right) in CH_3_CN.

To further probe the decay reactions, we monitored decay kinetics for 1.25 mM CH_3_CN solutions of each complex from 303–348 K in CH_3_CN under anaerobic conditions. At each temperature, the decay could be fit to a pseudo-first-order process, and the *k*_obs_ values at different temperatures were fit to the Eyring equation to obtain activation parameters (Fig. 9). This analysis yielded Δ*H*^‡^ = 21.4 ± 1.5 kcal mol^−1^, Δ*S*^‡^ = −9.5 ± 4.9 cal mol^−1^ K^−1^, and Δ*G*^‡^ = 24.2 ± 3.0 kcal mol^−1^ at 298 K for [Mn^III^(OO^*t*^Bu)(^6Me^dpaq)]^+^; and Δ*H*^‡^ = 23.5 ± 1.2 kcal mol^−1^, Δ*S*^‡^ = −1.5 ± 3.6 cal mol^−1^ K^−1^, and Δ*G*^‡^ = 23.9 ± 2.2 kcal mol^−1^ at 298 K for [Mn^III^(OOCm)(^6Me^dpaq)]^+^. Both complexes display Δ*H*^‡^ values significantly higher than those of the [Mn^III^(OOR)(N_4_S)]^+^ complexes (Δ*H*^‡^ = 15.9–10.5 kcal mol^−1^),^16^ which is in line with the greater thermal stabilities of [Mn^III^(OO^*t*^Bu)(^6Me^dpaq)]^+^ and [Mn^III^(OOCm)(^6Me^dpaq)]^+^. The entropies of activation of [Mn^III^(OO^*t*^Bu)(^6Me^dpaq)]^+^ and [Mn^III^(OOCm)(^6Me^dpaq)]^+^ are slightly negative and smaller in magnitude than those of the [Mn^III^(OOR)(N_4_S)]^+^ complexes (Δ*S*^‡^ = −15 to −34 cal mol^−1^ K^−1^).

### Substrate oxidation by Mn^III^–alkylperoxo adducts

#### 1 Direct oxidation of triphenylphosphine

**Fig. 10:**
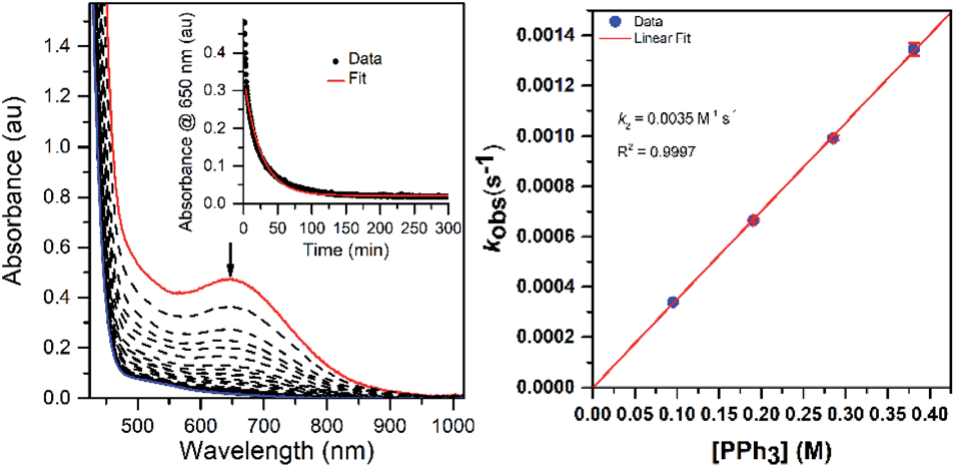
Left: electronic absorption spectra monitoring the reaction of a 2.0 mM anaerobic solution of [Mn^III^(OO^*t*^Bu)(^6Me^dpaq)]^+^ (red trace) in CH_3_CN with 100 equivalents of PPh_3_ at 298 K. Left inset: time course for the spectral changes. Right: pseudo-first-order rate constants, *k*_obs_ (s^−1^), *versus* PPh_3_ concentration (M) for a 1.25 mM solution of [Mn^III^(OO^*t*^Bu)(^6Me^dpaq)]^+^ at 298 K.

The addition of 100 equiv. PPh_3_ to an anaerobic solution of [Mn^III^(OO^*t*^Bu)(^6Me^dpaq)]^+^ (2.0 mM in CH_3_CN) at 298 K resulted in the loss of intensity at 650 nm over the course of two hours, resulting in an electronic absorption spectrum consistent with the generation of Mn^II^ products (Fig. 10, left). The decay of the 650 nm absorption signal could be well-fit to a first-order model, yielding a pseudo-first- order rate constant *k*_obs_ (Fig. 10, left inset). A ^31^P NMR analysis of the organic products revealed the formation of Ph_3_PO in 70% yield relative to the Mn^III^–alkylperoxo concentration (Fig. S24†). EPR analysis of the reaction mixture shows a signal centered at *g* = 2.03 that is similar in appearance to that of the [Mn^II^(H_2_O)(^6Me^dpaq)]OTf starting material, albeit with the lack of apparent hyperfine splitting (Fig. S25†).^28,39,50^ This evidence is in accordance with the featureless UV-vis spectrum of the final reaction mixture, which is characteristic of a Mn^II^ product (Fig. 10, left). The rate of decay of [Mn^III^(OO^*t*^Bu)(^6Me^dpaq)]^+^ increased linearly with increasing concentrations of PPh_3_ (Fig. 10, right). A linear fit of *k*_obs_*versus* PPh_3_ concentration yields a second-order rate constant for PPh_3_ oxidation by [Mn^III^(OO^*t*^Bu)(^6Me^dpaq)]^+^ of 0.0035 M^−1^s^−1^ at 298 K in CH_3_CN. The [Mn^III^(OOCm)(^6Me^dpaq)]^+^ complex displayed similar reactivity with PPh_3_ (*k*_2_ = 0.0033 M^−1^s^−1^ at 298 K; see Fig. S26†). These rates show that the nature of the alkylperoxo group does not have a significant influence on the reactivity with PPh_3_.

**Fig. 11:**
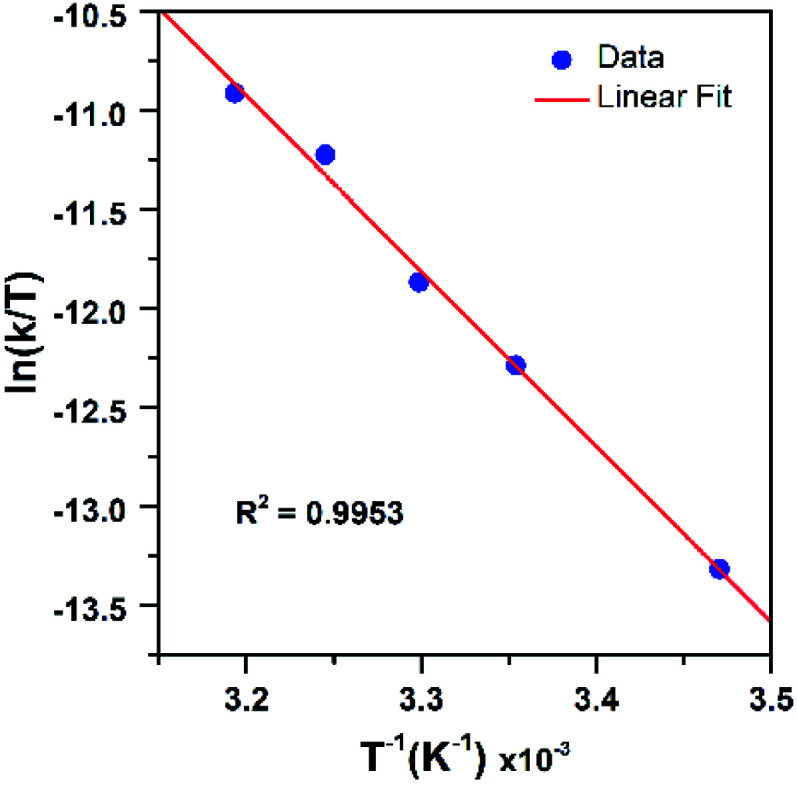
Eyring plot from variable-temperature reaction kinetics of [Mn^III^(OO^*t*^Bu)(^6Me^dpaq)]^+^ with PPh_3_ in CH_3_CN.

Further insight into the reaction of PPh_3_ with [Mn^III^(OO^*t*^Bu)(^6Me^dpaq)]^+^ was obtained through an Eyring analysis of variable-temperature kinetic experiments (Fig. 11). These experiments yielded Δ*H*^‡^ = 17.6 ± 1.4 kcal mol^−1^, Δ*S*^‡^ = −12.6 ± 4.6 cal mol^−1^ K^−1^, and Δ*G*^‡^ = 21.3 ± 2.8 kcal mol^−1^ at 298 K. The high activation enthalpy and Gibbs free energy of activation account for the sluggishness of this reaction, and the negative entropy of activation is consistent with a bimolecular reaction.

Mechanistic insight into the reaction between PPh_3_ and the Mn^III^–alkylperoxo was obtained by the reaction of 22 mM [Mn^III^(OOCm)(^6Me^dpaq)]^+^ with 5 equiv. of PPh_3_ in CH_3_CN at 298 K. Quantification of the organic products of this reaction by GC-MS revealed 88.5 ± 0.3% 2-phenyl-2-propanol and 1.5 ± 0.3% acetophenone based on the initial concentration of [Mn^III^(OOCm)(^6Me^dpaq)]^+^. ESI-MS analysis of the final reaction mixtures for the reaction of [Mn^III^(OOCm)(^6Me^dpaq)]^+^ with PPh_3_ showed the presence of peaks at *m*/*z* = 465.14, 482.14 and 743.23, which are consistent with expected *m*/*z* values for [Mn^II^(^6Me^dpaq)]^+^, [Mn^III^(OH)(^6Me^dpaq)]^+^ and [Mn(OPPh_3_)(^6Me^dpaq)]^+^, respectively (Fig. S27†).

#### 2 Indirect oxidation of 9,10-dihydroanthracene (DHA)

The reaction of the Mn^III^–alkylperoxo complexes with the hydrogen-atom donor DHA was also explored. In this case, the addition of a large excess of DHA (100 equiv. relative to Mn^III^) to [Mn^III^(OO^*t*^Bu)(^6Me^dpaq)]^+^ or [Mn^III^(OOCm)(^6Me^dpaq)]^+^ in CH_3_CN at 323 K did not result in any change in the rate of decay of the Mn^III^–alkylperoxo complexes (Fig. S28†). We also observed no change in the thermal decay rate with *d*_4_-DHA (Fig. S29†). Nonetheless, an analysis of the reaction solution following the full thermal decay of [Mn^III^(OO^*t*^Bu)(^6Me^dpaq)]^+^ or [Mn^III^(OOCm)(^6Me^dpaq)]^+^ (after *ca.* 7 hours) revealed the formation of *ca.* 1.4 equiv. anthracene relative to the starting Mn^III^–alkylperoxo concentration (Fig. S30†). Thus, while neither [Mn^III^(OO^*t*^Bu)(^6Me^dpaq)]^+^ nor [Mn^III^(OOCm)(^6Me^dpaq)]^+^ is capable of direct oxidation of DHA, a product of the thermal decay of each complex is an effective oxidant of DHA.

## Discussion

### Ligand-sphere influence on the structure–property correlations of Mn^III^–alkylperoxo complexes

The generation of the room-temperature stable [Mn^III^(OO^*t*^Bu)(^6Me^dpaq)]^+^ and [Mn^III^(OOCm)(^6Me^dpaq)]^+^ complexes relied upon previous observations that Mn^III^ centers with higher Lewis acidity give rise to corresponding Mn^III^–alkylperoxo adducts with shorter and more stable O–O bonds.^16^ The basis of this correlation rests on the π-donating properties of the alkylperoxo ligand. According to this model, which was originally proposed by Kovacs, DeBeer, and co-workers,^16^ a more Lewis acidic Mn^III^ center fosters greater π-donation from the alkylperoxo π* MO, which strengths the O–O bond. The 6-Me-pyridyl groups of the structurally characterized [Mn^III^(OOCm)(^6Me^dpaq)]^+^ complex give two elongated Mn–N distances of 2.284(4) and 2.394(4) Å. These weak metal–ligand interactions increase the Lewis acidity of the Mn^III^ center, stabilizing the Mn^III^–alkylperoxo unit. DFT computations lend credence to this model, showing a reduction in the Mulliken charge of [Mn^III^(OO^*t*^Bu)(^6Me^dpaq)]^+^ relative to [Mn^III^(OO^*t*^Bu)(dpaq)]^+^ (0.48 and 0.52, respectively). The computations also predict a greater admixture of alkylperoxo character in the Mn–OO^*t*^Bu π-antibonding MO for [Mn^III^(OO^*t*^Bu)(^6Me^dpaq)]^+^ (Fig. S33†), which supports stronger π-donation in this complex.

**Fig. 12:**
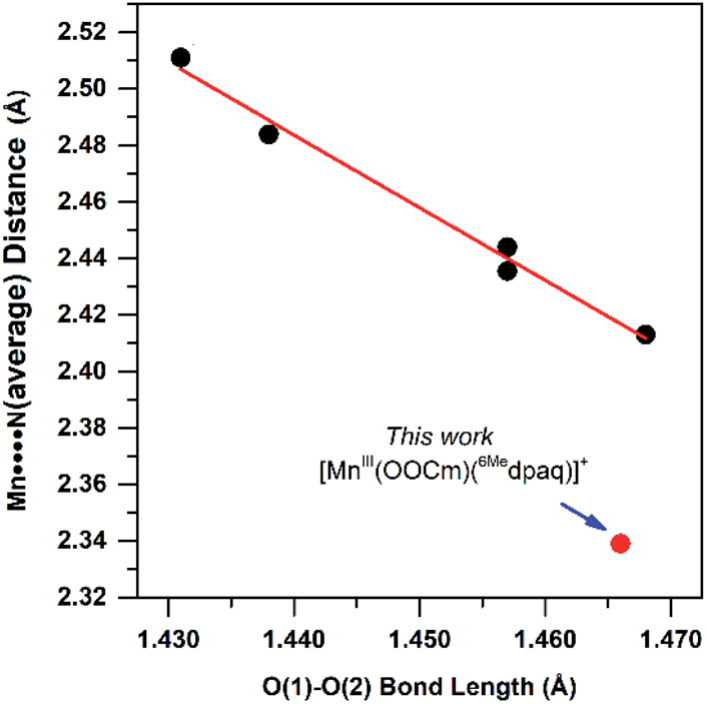
Correlation between average Mn–N distance and alkylperoxo O–O bond length for [Mn^III^(OOR)(N_4_S)]^+^ complexes (see ref. 16) and corresponding point for [Mn^III^(OOCm)(^6Me^dpaq)](OTf) described in this work.

The crystal structure of [Mn^III^(OOCm)(^6Me^dpaq)]^+^ allows us to determine how closely this complex follows previously observed correlations based on the [Mn^III^(OOR)(N_4_S)]^2+^ complexes. The [Mn^III^(OOR)(N_4_S)]^+^ complexes showed a linear correlation between the elongated Mn–N distances and the O–O bond lengths, with the shortest Mn–N distances of *ca.* 2.40 Å giving rise to the longest O–O bonds of *ca.* 1.47 Å (Fig. 12). The metric parameters for [Mn^III^(OOCm)(^6Me^dpaq)]^+^ follow the spirit of this correlation; that is, this complex has a short average Mn–N distance of 2.34 Å and an O–O bond of 1.466(4) Å, on the long end of that observed for Mn^III^–alkylperoxo adducts (1.43–1.47 Å). However, if we use the previous correlation as a guide, the Mn–N distances observed for [Mn^III^(OOCm)(^6Me^dpaq)]^+^ would predict a O–O bond length far longer than that observed, making this complex a clear outlier (Fig. 12). It is not completely surprising that the markedly different coordination spheres of [Mn^III^(OOCm)(^6Me^dpaq)]^+^ and the [Mn^III^(OOR)(N_4_S)]^+^ series would cause such a deviation, as the O–O distance should be a reporter of the entire coordination sphere. It is additionally possible that there is a limit to the extent to which the O–O bond can be elongated in Mn^III^–OOR complexes, and that the limit is near 1.47 Å.

The [Mn^III^(OOCm)(^6Me^dpaq)]^+^ complex also breaks the previously observed correlation that Mn^III^–alkylperoxo adducts with longer O–O bonds are less stable than those with shorter O–O bonds.^15^ [Mn^III^(OOCm)(^6Me^dpaq)]^+^ has an O–O distance at the long end of the [Mn^III^(OOR)(N_4_S)]^+^ series but has a room-temperature half-life of *ca*. 5 days. In contrast, the most stable [Mn^III^(OOR)(N_4_S)]^+^ complex has a half-life of *ca.* 5 minutes at 293 K.^16^ One caveat that must be noted in comparing the thermal stability of [Mn^III^(OOCm)(^6Me^dpaq)]^+^ with the [Mn^III^(OOR)(N_4_S)]^+^ series is the difference in solvents (CH_3_CN and CH_2_Cl_2_, respectively).^16^ To address this complication, we determined the half-life of [Mn^III^(OOCm)(^6Me^dpaq)]^+^ in CH_2_Cl_2_ at 298 K. The [Mn^III^(OOCm)(^6Me^dpaq)]^+^ complex did decay more rapidly in CH_2_Cl_2_ than in CH_3_CN (half-life of 3 *vs.* 8 days, respectively). While solvent does have some effect on the stability of the Mn^III^–alkylperoxo complex, the solvent change alone cannot account for the dramatic increase in stability of the [Mn^III^(OOR)(^6Me^dpaq)]^+^ complexes relative to the [Mn^III^(OOR)(N_4_S)]^+^ series. On this basis, while also noting the limited sample size, it is tempting to speculate that the presence of thiolate ligands in the [Mn^III^(OOR)(N_4_S)]^2+^ series severely reduces the stability of the Mn^III^–alkylperoxo adducts. This conclusion makes it all the more remarkable that the first isolable Mn^III^–alkylperoxo adducts contained thiolate ligands.

### Thermal decay mechanism of [Mn^III^(OOCm)(^6Me^dpaq)]^+^

**Scheme 2:**
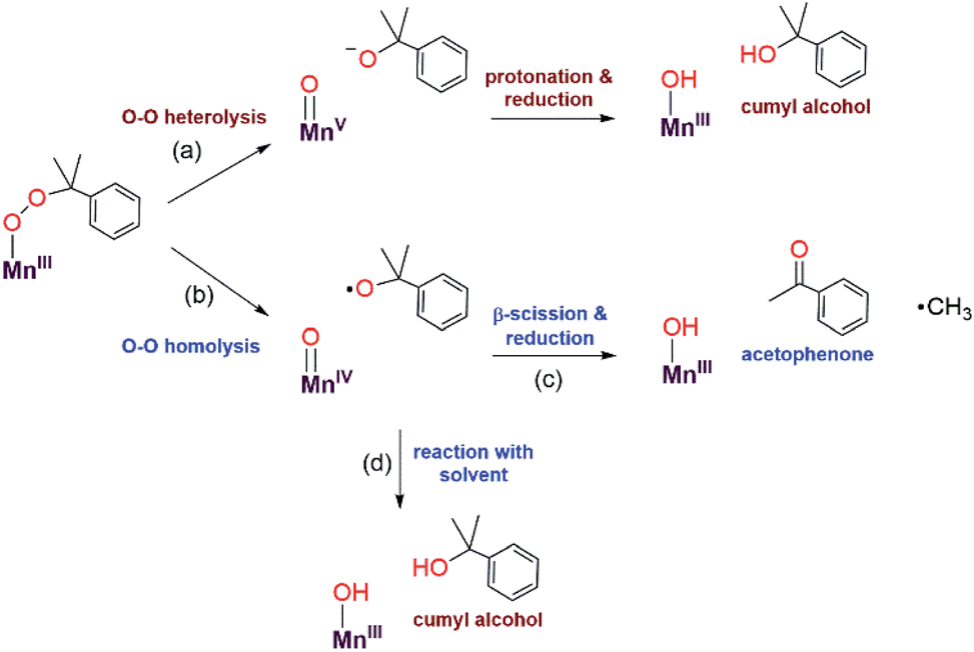
Possible decay pathways for [Mn^III^(OOCm)(^6Me^dpaq)](OTf).

The organic products observed upon the decay of cumylperoxo–metal complexes are often used to infer the nature of the decay pathway.^51^ Homolytic cleavage of the O–O bond produces cumyloxyl radical that can rearrange by β-scission to produce acetophenone and ˙CH_3_ (Scheme 2, path b). Alternatively, heterolytic cleavage of the O–O bond produces cumyl oxyanion that can deprotonate solvent to produce 2-phenyl-2-propanol (Scheme 2, path a).^51–53^ Previous studies of Mn^III^–alkylperoxo^16^ and some Fe^III^–alkylperoxo^12^ complexes showed organic products exclusively attributable to O–O homolysis. In contrast, there are examples of Cu^II^–alkylperoxo^54^ adducts that decay exclusively by heterolytic cleavage of the O–O bond, and there are a handful of examples where the decay of a metal–alkylperoxo adduct yields a mixture of products characteristic of both pathways.^2,5,11,13,55^

Analysis of the organic decay products of [Mn^III^(OOCm)(^6Me^dpaq)]^+^ reveal both 2-phenyl-2-propanol and acetophenone (61 and 26% yield relative to the [Mn^III^(OOCm)(^6Me^dpaq)]^+^adduct). Electronic absorption, ^1^H NMR, and ESI-MS data identify [Mn^III^(OH)(^6Me^dpaq)]^+^ as a dominant decay product (Fig. S20 and S21†). The distribution of 2-phenyl-2-propanol and acetophenone could suggest that both homolysis and heterolysis of the O–O bond occur in the thermal decay of [Mn^III^(OOCm)(^6Me^dpaq)]^+^ in CH_3_CN, with a preference for the heterolytic pathway. Either O–O cleavage pathway would yield a high-valent Mn–oxo intermediate that could be reduced to the observed Mn^III^–hydroxo product. There are recent reports of Mn^IV^–oxo adducts of the closely related dpaq ligand that react with C–H bonds to yield a Mn^III^–hydroxo product.^56,57^

To clarify the decay pathway of [Mn^III^(OOCm)(^6Me^dpaq)]^+^, we examined the products formed when the complex decayed in CD_3_CN. In this case, we observed increased formation of acetophenone and decreased formation of 2-phenyl-2-propanol (40 : 50%) compared to the decay in CH_3_CN (26 : 61%). A change in product distribution in deuterated solvent was also observed by Cho *et al.* in their investigations of a Cu^II^–alkylperoxo complex.^13^ Itoh^2,3^ and others^51,52^ have rationalized a change in the acetophenone: 2-phenyl-2-propanol distribution in terms of solvent involvement in the decay pathway. In CH_3_CN, the cumyloxyl radical decays by competing reactions: (1) β-scission to yield acetophenone and methyl radical, and (2) hydrogen-atom abstraction from solvent to yield 2-phenyl-2-propanol (Scheme 2, paths c and d).^2,3,51,54,55,58^ In CD_3_CN, the rate of the hydrogen-atom abstraction reaction from the solvent is decreased, yielding a marked increase in acetophenone formation by β-scission. When [Mn^III^(OOCm)(^6Me^dpaq)]^+^ decays in PhCN, we observe an even greater increase in acetophenone formation (70%), with only 30% formation of 2-phenyl-2-propanol. We attribute this change to the strong C–H bonds in PhCN that suppress the reaction of solvent with the cumyloxyl radical (Scheme 2, path d). Taken together, the change in the distribution of acetophenone: 2-phenyl-2-propanol in CD_3_CN and PhCN is strong evidence that [Mn^III^(OOCm)(^6Me^dpaq)]^+^ decays, at least to some extent, by O–O homolysis. However, the persistence of a significant fraction of 2-phenyl-2-propanol product, even in CD_3_CN and PhCN, suggests that the thermal decay of [Mn^III^(OOCm)(^6Me^dpaq)]^+^ also involves O–O heterolysis. Thus, our data provide evidence for both decay pathways in Scheme 2, each of which yields [Mn^III^(OH)(^6Me^dpaq)]^+^ as the Mn-containing product. This situation is distinct from that observed for [Mn^III^(OOR)(N_4_S)]^+^ complexes,^16^ where the decay proceeded exclusively by O–O homolysis and yielded a mixture of Mn-containing products.

A second line of evidence for the production of cumyloxyl radicals by O–O homolysis of [Mn^III^(OOCm)(^6Me^dpaq)]^+^ comes from changes to the decay kinetics of [Mn^III^(OOCm)(^6Me^dpaq)]^+^ in CH_3_CN and CD_3_CN. The [Mn^III^(OOCm)(^6Me^dpaq)]^+^ complex decays slowly in CH_3_CN, and this rate of decay matches the rate of formation of the [Mn^III^(OH)(^6Me^dpaq)]^+^ product (Fig. 8). In contrast, the decay rate of [Mn^III^(OOCm)(^6Me^dpaq)]^+^ increases in CD_3_CN by about eight-fold relative to that in CH_3_CN (Fig. S34†). In addition, the decay rate of [Mn^III^(OOCm)(^6Me^dpaq)]^+^ in CD_3_CN is five-fold faster than the rate of formation of [Mn^III^(OH)(^6Me^dpaq)]^+^. These observations are consistent with our proposal that a fraction of the [Mn^III^(OOCm)(^6Me^dpaq)]^+^ complex decays by homolytic O–O cleavage to give a Mn^IV^–oxo adduct and cumyloxyl radical. In CH_3_CN, the cumyloxyl radical and Mn^IV^–oxo intermediates react rapidly and preferentially with solvent to give the observed [Mn^III^(OH)(^6Me^dpaq)]^+^ and 2-phenyl-2-propanol products. Under these conditions, a relatively small amount of cumyloxyl radical undergoes β-scission to yield acetophenone. In CD_3_CN, the Mn^IV^–oxo adduct and cumyloxyl radical decay products have slower rates of reaction with solvent, allowing for reaction with [Mn^III^(OOCm)(^6Me^dpaq)]^+^, which hastens its decay.

### Reaction mechanism of [Mn^III^(OOCm)(^6Me^dpaq)]^+^ with PPh_3_

To the best of our knowledge, the reactions of [Mn^III^(OO^*t*^Bu)(^6Me^dpaq)]^+^ and [Mn^III^(OOCm)(^6Me^dpaq)]^+^ with PPh_3_ at 298 K are the first observations of direct substrate oxidation by Mn^III^–alkylperoxo complexes. The reaction of [Mn^III^(OOCm)(^6Me^dpaq)]^+^ with PPh_3_ showed the near exclusive formation of 2-phenyl-2-propanol, with only a trace amount of acetophenone (Fig. S35†). This distribution suggests a change to O–O heterolysis under these conditions. An Eyring analysis for the reaction of [Mn^III^(OO^*t*^Bu)(^6Me^dpaq)]^+^ with PPh_3_ gives Δ*S*^‡^ = −12.6 ± 4.6 cal mol^−1^ K^−1^, which is consistent with a bimolecular reaction involving the association of [Mn^III^(OO^*t*^Bu)(^6Me^dpaq)]^+^ with PPh_3_ to form the activated complex. The change in reaction rate as a function of PPh_3_ concentration is further evidence of a direct reaction between the [Mn^III^(OOR)(^6Me^dpaq)]^+^ complexes and PPh_3_. In addition, an ESI-MS analysis of the products of the reaction of [Mn^III^(OO^*t*^Bu)(^6Me^dpaq)]^+^ with PPh_3_ revealed a peak for [Mn(OPPh_3_)(^6Me^dpaq)]^+^ that shifts by +2 mass units when the Mn^III^–alkylperoxo adduct is prepared using ^*t*^Bu^18^O^18^OH (Fig. S27†). Thus, the oxygen in the OPPh_3_ product derives from the Mn^III^–alkylperoxo unit.

**Scheme 3:**
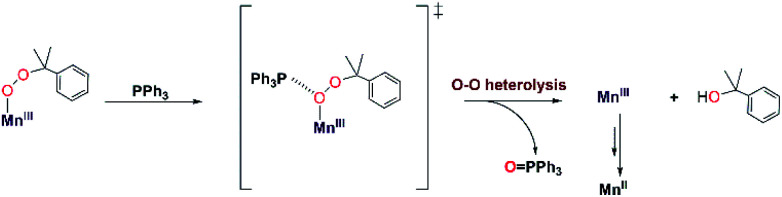
Proposed mechanisms for PPh_3_ oxidation by [Mn^III^(OOCm)(^6Me^dpaq)]^+^.

We propose a reaction mechanism where [Mn^III^(OOR)(^6Me^dpaq)]^+^ and PPh_3_ form an activated complex, with PPh_3_ interacting with the proximal oxygen of the alkylperoxo ligand (Scheme 3). Recent reports show that Brønsted and Lewis acids, or the introduction of secondary coordination interaction through pendant amines which act as hydrogen-bond acceptor in an Fe^III^–OOR (R = H, acyl) adduct could direct heterolytic cleavage.^11,59–61^ This interaction between [Mn^III^(OOR)(^6Me^dpaq)]^+^ and PPh_3_ may also be able to instigate heterolytic cleavage of the Mn^III^–alkylperoxo O–O bond. For the [Mn^III^(OOCm)(^6Me^dpaq)]^+^ complex, this decay will lead to the formation of cumyloxy anion, which gives 2-phenyl-2-propanol after protonation^2,54,55^ and a Mn^III^-species that is reduced to the Mn^II^ product observed by UV-vis and EPR spectroscopy (Scheme 3). The identity of the reductant for the Mn^III^ center is unclear.

### Reaction mechanism of [Mn^III^(OOCm)(^6Me^dpaq)](OTf) with DHA

**Scheme 4:**
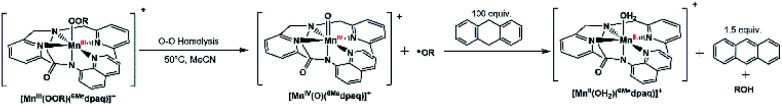
Mechanistic proposal for the reaction of DHA with [Mn^III^(OO^*t*^Bu)(^6Me^dpaq)]^+^.

In contrast to the direct oxidation of PPh_3_, the reaction of [Mn^III^(OO^*t*^Bu)(^6Me^dpaq)]^+^ and [Mn^III^(OOCm)(^6Me^dpaq)]^+^ with DHA is an indirect oxidation caused by products of the Mn^III^–alkylperoxo decay process (Scheme 4). Neither [Mn^III^(OOR)(^6Me^dpaq)]^+^ complex shows any change in decay rate in the presence of an excess amount of DHA (Fig. S29†), although the decay solutions reveal the formation of 1.4 equiv. anthracene relative to the initial Mn^III^–OOR concentration. The notion that the reaction is indirect is further supported by the lack of any change in decay rate when *d*_4_-DHA is used as substrate.

The formation of 1.4 equiv. anthracene is consistent with the thermal decay of the [Mn^III^(OOR)(^6Me^dpaq)]^+^ complexes by O–O homolysis (Scheme 4). The Mn^IV^–oxo decay product should be capable of DHA oxidation, as observed for several oxo–manganese complexes.^56,57^ This reaction will result in a Mn^II^–aqua complex, consistent with the observation of a Mn^II^ signal in the EPR spectrum of the final reaction mixture (Fig. S30 and S31†). The cumyloxyl radical also generated by O–O homolysis could be responsible for the remaining 0.4 equiv. anthracene.

## Conclusions

Inspired by previously developed structure-reactivity correlations, we developed a new ligand derivative (^6Me^dpaq) that provides remarkable stability to Mn^III^–alkylperoxo complexes. A simple change of two pyridyl groups to 6-Me-pyridyl groups results in new Mn^III^–alkylperoxo complexes that (i) can be generated using stoichiometric amounts of oxidant rather than large excesses, and (ii) are stable at room temperature. This enhanced stability allowed us to structurally characterize a Mn^III^–cumylperoxo adduct by X-ray diffraction. In spite of the unusual stability of these Mn^III^–alkylperoxo adducts, these complexes are the first members of their class to show direct reactivity with a substrate (triphenylphosphine). This result demonstrates that the ligand-sphere of Mn^III^–alkylperoxo adducts has great control over reactivity. Examination of the thermal decay of these N_5_^−^-ligated Mn^III^–alkylperoxo adducts provides evidence from both homolytic and heterolytic O–O bond cleavage, which is distinct from that observed for Mn^III^–alkylperoxo adducts bound by thiolate-containing N_4_S^−^ ligands.

While the basis of the enhanced stability of [Mn^III^(OO^*t*^Bu)(^6Me^dpaq)]^+^ and [Mn^III^(OOCm)(^6Me^dpaq)]^+^ will be the subject of future investigations, it is tempting to speculate that the thiolate ligands in the [Mn^III^(OOR)(N_4_S)]^+^ complexes serve to lower the activation energy for decay. In addition, while the new Mn^III^–alkylperoxo adducts generally follow a previously identified structural correlation between Mn–N and O–O distances, the observed O–O distance for the Mn^III^–cumylperoxo adduct is far shorter than expected on the basis of the Mn–N distances. Thus, perturbations to the primary coordination sphere not only affect reactivity but also render this complex an outlier compared to previous compounds. Future work will be aimed at understanding the basis for this outlier status in terms of both structural correlations and chemical reactivity.

## Data availability

Crystallographic data are available through the Cambridge Crystallographic Data Centre (CCDC) at https://www.ccdc.cam.ac.uk/ with structure codes 2048663, 2049911, and 2048664.

## Author contributions

A. A. O., J. D. P. and T. A. J. conceived and planned the experiments. A. A. O. and J. D. P. performed all experiments and computations, except those involving X-ray crystallography, which were performed by V. W. D. All authors contributed to data analysis and provided contributions to writing of the final manuscript.

## Conflicts of interest

There are no conflicts to declare.

## Supplementary Material

SC-012-D1SC01976G-s001

SC-012-D1SC01976G-s002
