# Supplementary material for: Characterization and chemical reactivity of room-temperature-stable MnIII–alkylperoxo complexes
Source: Chem Sci. 2021 Aug 20;12(38):12564–75. doi: 10.1039/d1sc01976g (PMC8494025; doi:10.1039/d1sc01976g)
Supplement: SC-012-D1SC01976G-s001 [file SC-012-D1SC01976G-s001.pdf]

Electronic Supplementary Material (ESI) for Chemical Science.
This journal is © The Royal Society of Chemistry 2021

Supplementary Information for

Characterization and Chemical Reactivity of Room-Temperature-Stable Mn^{III}-alkylperoxo Complexes

Adedamola A. Opalade,[a] Joshua D. Parham,[a] Victor W. Day[a] and Timothy A. Jackson *[a]

[a] *The University of Kansas, Department of Chemistry and Center for Environmentally Beneficial Catalysis, 1567 Irving Hill Road, Lawrence, KS 66045, USA.*

Contents

Experimental Details and Methods. 4

Synthesis of (H^{6Me}dpaq). 4

Fig. S1. ^{1}H NMR spectrum of H^{6Me}dpaq. 6

Fig. S2. ^{13}C NMR spectrum of H^{6Me}dpaq. 6

Fig. S3. HSQC NMR data for H^{6Me}dpaq. 7

Synthesis and Characterization of $[Mn^{II}(OH_2)(^{6Me}dpaq)](OTf)$. 7

X-ray diffraction data collection and analysis for $[Mn^{II}(H_2O)(^{6Me}dpaq)](OTf)$. 8

Fig. S4. XRD structures of $[Mn^{II}(dpaq)](OTf)$ and $[Mn^{II}(dpaq^{2Me})](OTf)$ 9

Fig. S5. EPR spectrum of $[Mn^{II}(H_2O)(^{6Me}dpaq)]OTf$ 10

Fig. S6. ESI-MS spectrum of $[Mn^{II}(^{6Me}dpaq)]^{+}$. 10

Fig. S7. Evans NMR of $[Mn^{II}(H_2O)(^{6Me}dpaq)]OTf$ 11

Synthesis and Characterization of $[Mn^{III}(OH)(^{6Me}dpaq)](OTf)$. 12

XRD Analysis of $[Mn^{III}(OH)(^{6Me}dpaq)](OTf)$. 12

Fig. S8. Electronic absorption spectra for formation of $[Mn^{III}(OH)(^{6Me}dpaq)](OTf)$ 13

Fig. S9. ESI-MS of $[Mn^{III}(OH)(^{6Me}dpaq)]^{+}$ in CH_3CN 14

Fig. S10. Evans NMR of $[Mn^{III}(OH)(^{6Me}dpaq)]OTf$ in CD_3CN 14

Fig. S11. H-bonding interaction in $[Mn^{III}(OH)(^{6Me}dpaq)]$+ and $[Mn^{III}(OH)(^{2Me}dpaq)]$+ 15

Table S1. Comparism of structural parameters in Mn-complexes 15

Synthesis and Characterization of $[Mn^{III}(OOR)(^{6Me}dpaq)]^{+}$ 16

X-ray diffraction data collection and analysis for $[Mn^{III}(OOCm)(^{6Me}dpaq)](OTf)$. 16

Fig. S12. Uv-vis titration for the formation of $[Mn^{III}(OO^{t}Bu)(^{6Me}dpaq)]^{+}$ 17

Fig. S13. Uv-vis spectrum for the formation of $[Mn^{III}(OOR)(^{6Me}dpaq)]^{+}$ 18

Fig. S14. ESI-MS of $[Mn^{III}(OO^{t}Bu)(^{6Me}dpaq)]^{+}$ and $[Mn^{III}(OOCm)(^{6Me}dpaq)]^{+}$ 18

Fig. S15. Evans NMR Data for $[Mn^{III}(OO^{t}Bu)(^{6Me}dpaq)]OTf$ in CD_3CN 19

Fig. S16. Evans NMR Data for $[Mn^{III}(OOCm)(^{6Me}dpaq)]OTf$ in CD_3CN 20

Fig. S17. ESI-MS Data for labeled and unlabeled $[Mn^{III}(OO^{t}Bu)(^{6Me}dpaq)]OTf$ 21

Fig. S18. IR Data for labeled and unlabeled $[Mn^{III}(OO^{t}Bu)(^{6Me}dpaq)]OTf$ 22

Table S2. Uv-vis, structural, and IR data for Mn^{III}-alkylperoxo Complexes 22

Fig. S19. X-band EPR spectra of $[Mn^{III}(OOR)(^{6Me}dpaq)]^+$ 23

Decay kinetics. 23

Fig. S20. ESI-MS for $[Mn^{III}(OOR)(^{6Me}dpaq)]^+$ decay products 23

Fig. S21. ^{1}H NMR spectra of $[Mn^{III}(OOR)(^{6Me}dpaq)]^+$ (R = H, OtBu and OCm) 24

Fig. S22. Uv-vis spectra for the decay of crude $[Mn^{III}(OOCm)(^{6Me}dpaq)]^+$ 25

PPh3 reaction kinetics and Eyring analysis. 25

Fig. S23. Decay of $[Mn^{III}(OOCm)(^{6Me}dpaq)]^+$ in Benzonitrile 26

Fig. S24. ^{31}P NMR of the reaction product of PPh_3 with $[Mn^{III}(OO^tBu)(^{6Me}dpaq)]^+$ 26

Fig. S25. EPR of $[Mn^{II}(H_2O)(^{6Me}dpaq)]^+$ and reaction product of $[Mn^{III}(OO^tBu)(^{6Me}dpaq)]^+$ with PPh_3 27

Fig. S26. k_{obs} (s^{-1}), vs. PPh_3 concentration reaction with of $[Mn^{III}(OOCm)(^{6Me}dpaq)]$+ 28

Fig. S27. ESI-MS of reaction product of labeled and unlabeled $[Mn^{III}(OOR)(^{6Me}dpaq)]^+$ with PPh_3 29

Reactivity of $[Mn^{III}(OO^tBu)(^{6Me}dpaq)]^+$ and $[Mn^{III}(OOCm)(^{6Me}dpaq)]^+$ with DHA. 30

Fig. S28. Uv-vis of the reaction of $[Mn^{III}(OO^tBu)(^{6Me}dpaq)]^+$ with DHA at 323 K 31

Fig. S29. Time trace for the reaction of $[Mn^{III}(OO^tBu)(^{6Me}dpaq)]^+$ with DHA, d_4-DHA at 323 K and the decay of $[Mn^{III}(OO^tBu)(^{6Me}dpaq)]^+$ at 323 K 31

Fig. S30. ^{1}H-NMR for the reaction of DHA with $[Mn^{III}(OO^tBu)(^{6Me}dpaq)]^+$ 32

Fig. S31. X-band EPR spectrum of $[Mn^{III}(OO^tBu)(^{6Me}dpaq)]^+$ with DHA 33

Fig. S32. Control experiments: DHA without $[Mn^{III}(OO^tBu)(^{6Me}dpaq)]^+$ at 323 K irradiated with UV light at 323 K 33

Fig. S33. Molecular orbitals plots of the Mn^{III}-alkylperoxo p-antibonding MOs of $[Mn^{III}(OO^tBu)(^{6Me}dpaq)]^+$ and $[Mn^{III}(OO^tBu)(dpaq)]^+$ 34

Fig. S34. Thermal decay of $[Mn^{III}(OOCm)(^{6Me}dpaq)]^+$ in CH_3CN and CD_3CN 35

Fig. S35. ^{1}H NMR of the reaction of $[Mn^{III}(OOCm)(^{6Me}dpaq)]^+$ with PPh_3 (organics) 35

Table S3. Crystal and refinement data for $[Mn^{II}(H_2O)(^{6Me}dpaq)](OTf)$, $[Mn^{III}(OH)(^{6Me}dpaq)](OTf)$, and $[Mn^{III}(OOCm)(^{6Me}dpaq)](OTf)$ 36

References 37

Experimental Details and Methods.

General Methods:

All chemicals were used as obtained from commercial sources unless noted otherwise. Acetonitrile, diethyl diethyl ether, and methanol were dried and degassed using a PureSolv Micro solvent purification system. 1,4-Benzoquine was purified by sublimation. Bis((6-methylpyridin-2-yl)methyl)amine was synthesized according to a reported procedure.[1] The concentration of *tert*-butyl hydroperoxide (^{t}BuOOH) in decane stock solution was found to be 4.3 M by iodometric titration.[2] ^{t}Bu^{18}O^{18}OH was synthesized following a previously published procedure.[3] Experiments were performed under dinitrogen atmosphere in a glovebox unless otherwise noted.

Instrumentation:

Electronic absorption experiments were performed using a Varian Cary 50 Bio UV−visible spectrophotometer equipped with a Unisoku cryostat and stirrer. Vibrational data were obtained using a PerkinElmer Spectrum100 FTIR spectrometer with samples sealed in 0.1 mm gastight NaCl cells. Electrospray ionization mass spectrometry (ESI-MS) experiments were performed using an LCT Premier MicroMass electrospray time-of-flight instrument. X-band EPR experiments were performed using a Bruker EMXplus with Oxford ESR900 continuous-flow liquid helium cryostat and an Oxford ITC503 temperature system. ^{1}H and ^{31}P NMR spectra were obtained on a Bruker DRX 400 MHz NMR spectrometer. ^{13}C and HSQC NMR spectra were obtained on an Avance AVIII 500 MHz NMR spectrometer. Hyperfine shifted ^{1}H NMR data were collected within the spectra width of 150 to -100 ppm with 1000 scans to provide sufficient S/N. Spectra were baseline subtracted with the multipoint fitting procedure using the spline functions in the MestReNova program. GC analysis was performed on the Agilent 6890N gas chromatograph coupled to a triple quadrupole mass analyzer with both electron impact and chemical ionization sources. X-ray crystallography experiments were performed on a Bruker diffractometer equipped with Helios high-brilliance multilayer optics, a platinum CCD detector and a Bruker MicroStar microfocus rotating anode X-ray source operating at 45 kV and 60 mA.

Synthesis of 2-(bis((6-methylpyridin-2-yl)methyl)amino)-N-(quinolin-8-yl)acetamide (H^{6Me}dpaq). The H^{6Me}dpaq ligand was synthesized according to a modified literature procedure.[4]

Under an inert atmosphere in a Schlenk flask, 0.879 g (6.1 mmol) 8-aminoquinoline and 1.238 g (11.7 mmol) sodium carbonate were dissolved in 20 mL CH_3CN. The solution was cooled to 273 K in an ice bath, and 1.231 g (6.1 mmol) bromoacetyl bromide was added dropwise to the cooled solution. The reaction was stirred for one hour at 273 K. The reaction mixture was filtered through an ultrafine frit, and the solvent was removed under vacuum. The resulting orange solid was combined in a flask with 1.002 g (9.5 mmol) sodium carbonate and dissolved in 40 mL CH_3CN under an inert atmosphere. The solution was cooled to 273 K in an ice bath. 1.387 g (6.1 mmol) of bis((6-methylpyridin-2-yl)methyl)amine was added slowly while stirring, and the reaction mixture was stirred overnight at 273 K. After *ca.* 20 hours, the reaction mixture was filtered through an ultrafine frit, and the solvent was removed under vacuum. The resulting red solid was purified through column chromatography on neutral alumina as the stationary phase and 99:1% vol:vol CH_2Cl_2:MeOH as the mobile phase. The purification was completed with 98:2% vol:vol CH_2Cl_2:MeOH. The final product was obtained as a dark yellow solid in 80% yield and characterized by ^{1}H, ^{13}C, and HSQC NMR methods (Fig. S1 – S3). ^{1}H NMR data (400 MHz) for H^{6Me}dpaq ($CDCl_3$, δ) = 11.61 (s, 1H), 8.94 (dd; *J* = 4.2, 1.7 Hz;1H), 8.75 (dd; *J* = 6.1, 2.9 Hz; 1H), 8.19 (dd; *J* = 8.3, 1.7 Hz; 1H), 7.82 (d; *J* = 7.7 Hz; 2H), 7.52 (m, 5H), 7.02 (m, 2H), 3.99 (s, 4H), 3.52 (s, 2H), 2.47 (s, 6H) ppm. ^{13}C NMR data (125 MHz) for H^{6Me}dpaq ($CDCl_3$, δ) = 169.92 (s, C=O), 157.88 (s, Py), 157.80 (s, Py), 148.19 (s, Qu), 139.02 (s, Qu), 137.01 (s, Qu), 136.42 (s, Qu), 134.58 (s, Qu), 128.22 (s, Qu), 127.58 (s; Qu), 121.98 (s; Py), 121.73 (d; Py,Qu), 120.30 (s, Py), 116.65 (s, Qu), 61.48 (-CH_2Py), 59.39 (-CH_2CO-), 24.50 (CH_3Py) ppm.

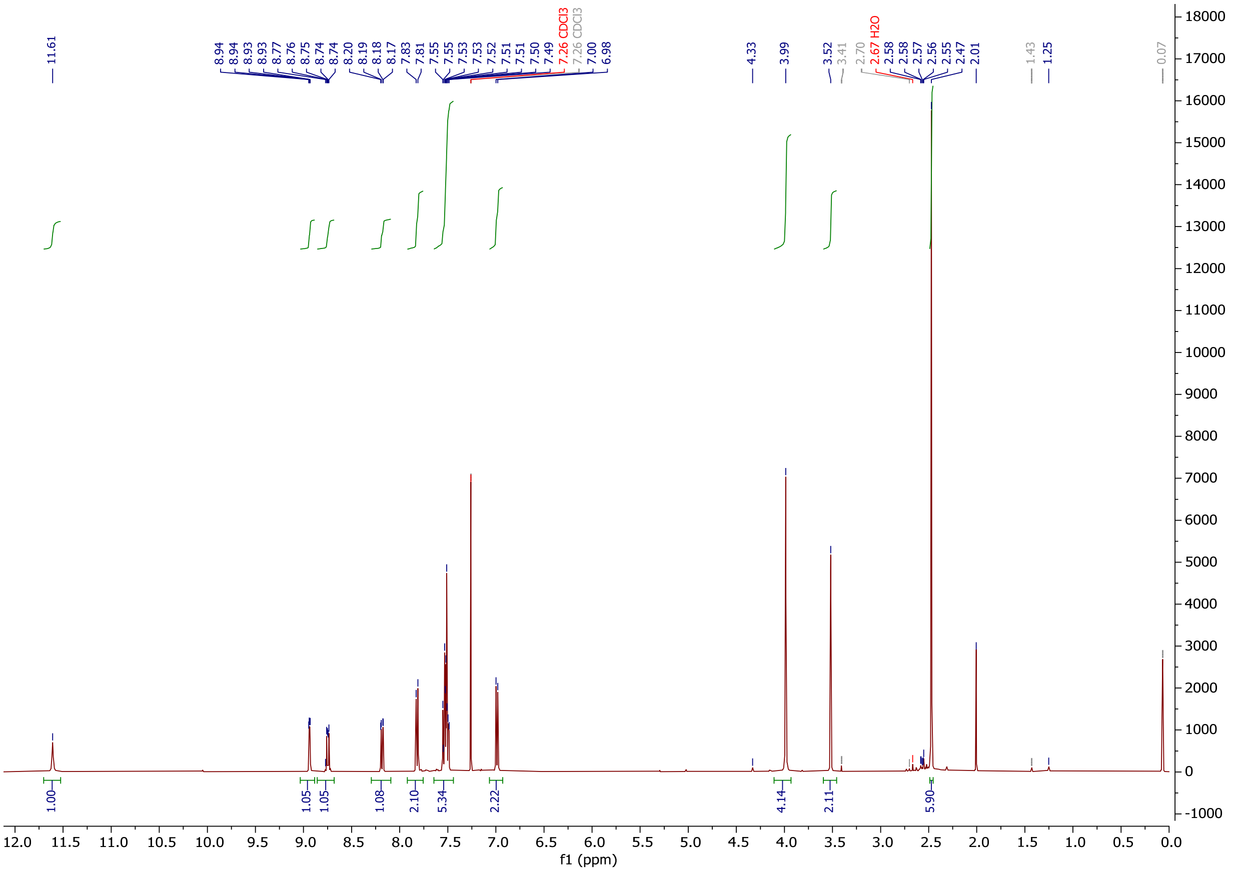

Fig. S1. ^{1}H NMR spectrum of H^{6Me}dpaq dissolved in $CDCl_3$ at 298 K.

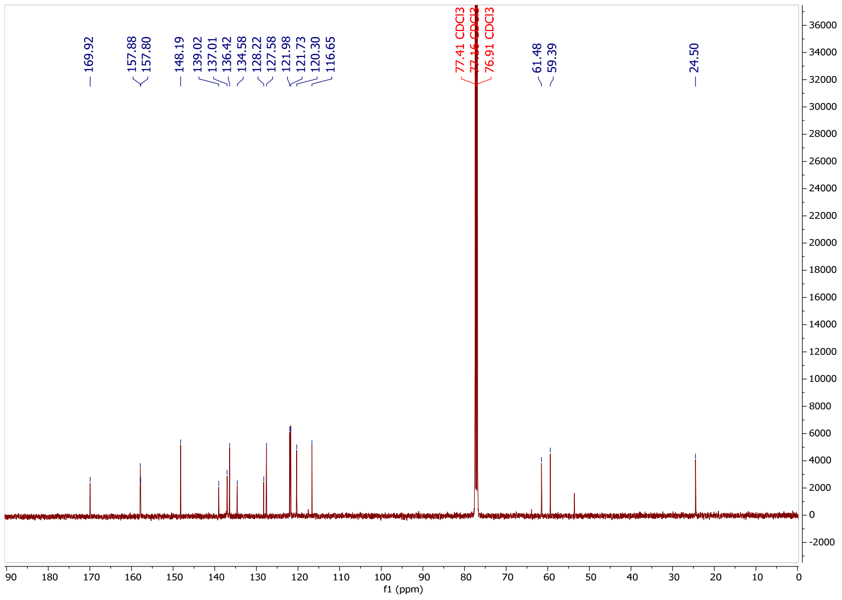

Fig. S2. ^{13}C NMR spectrum of H^{6Me}dpaq dissolved in $CDCl_3$ at 298 K.

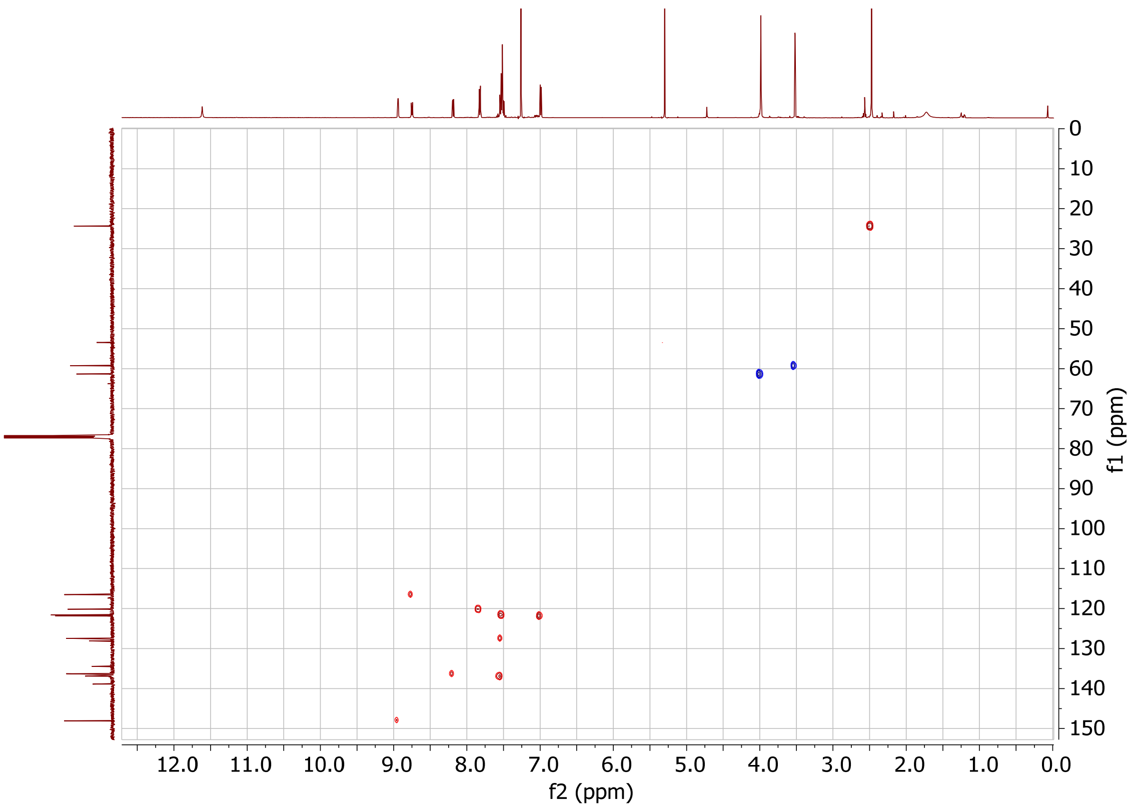

Fig. S3. HSQC NMR data for H^{6Me}dpaq dissolved in $CDCl_3$ at 298 K.

Synthesis and Characterization of [Mn^{II}(OH$_2$)(^{6Me}dpaq)](OTf). The reaction of 0.545 g (1.3 mmol) H^{6Me}dpaq with 0.577 g (1.3 mmol) Mn^{II}(OTf)$_2$·2CH$_3$CN in 40 mL MeOH under an inert atmosphere using 0.128 g (1.3 mmol) NaO^{t}Bu as a base stirred for 18 hours yields a bright orange solution. The MeOH was removed completely in vacuo leaving behind an orange powder. The orange powder was dissolved in a minimal amount of CH_3CN and layered with diethyl ether. This procedure led to the formation of an orange precipitate. The solvent was decanted, and the orange solid was dried, washed with diethyl ether, and dried again. The recrystallization procedures were repeated two more times and orange microcrystalline solid was obtained. The microcrystalline material was dissolved in a minimal amount of CH_3CN and set-up for crystal growth by slow vapor diffusion of diethyl ether into the CH_3CN solution. Single crystals suitable for X-ray crystallographic analysis were obtained by this method.

X-band, perpendicular-mode EPR data obtained for a *ca.* 2 mM solution of [Mn^{II}(H$_2$O)(^{6Me}dpaq)]OTf in CH_3CN reveals a 6-line signal (g = 2.00, A = 93.9G; Fig. S5). The g-

value and hyperfine splitting observed in this EPR spectrum are similar to those reported for the high-spin $[Mn^{II}(N4S)]^+$ complexes (g = 1.98 - 2.00, A = 90 – 100 G) and $[Mn^{II}(NCMe)(dpaq^{2Me})]^+$ (g = 2.04).[5, 6] Thus, we conclude that the monomeric structure observed in the X-ray structure of $[Mn^{II}(H_2O)(^{6Me}dpaq)](OTf)$ is retained in solution. In addition, a determination of the magnetic moment for this complex by the Evans method yielded a value of 5.5 μ_B, which is consistent with the assignment of this species as a high-spin Mn^{II} complex (the calculated spin-only value for an S = 5/2 species is 5.91 μ_B, see Fig. S7). Mass-spectral analysis of a CH_3CN solution of $[Mn^{II}(H_2O)(^{6Me}dpaq)](OTf)$ revealed a peak at m/z = 465.12, consistent with the $[Mn^{II}(^{6Me}dpaq)]^+$ ion (calculated m/z = 465.14; Fig. S6). The ESI-MS data also show an m/z peak at 482.14, which is consistent with $[Mn^{III}(OH)(^{6Me}dpaq)]^+$ (calculated m/z = 482.15). The appearance of $[Mn^{III}(OH)(^{6Me}dpaq)]^+$ indicates the oxidation of $[Mn^{II}(H_2O)(^{6Me}dpaq)]$OTf by ambient oxygen in air to form $[Mn^{III}(OH)(^{6Me}dpaq)]^+$. This phenomenon has been observed in other Mn^{II} complexes with similar ligands.[5, 7]

X-ray diffraction data collection and analysis for $[Mn^{II}(OH_2)(^{6Me}dpaq)](OTf)$. Complete sets of unique reflections were collected with monochromated CuKα radiation for a crystal sample of the $[Mn^{II}(H_2O)(^{6Me}dpaq)](OTf)$ compound. The $[Mn^{II}(H_2O)(^{6Me}dpaq)](OTf)$ crystal was a 95/5 racemic twin. Totals of 1639 1.0°-wide ω- or ϕ-scan frames with counting times of 4-6 seconds were collected for $[Mn^{II}(H_2O)(^{6Me}dpaq)](OTf)$ with a Bruker APEX II CCD area detector. X-rays were provided by a Bruker MicroStar microfocus rotating anode operating at 45kV and 60 mA and equipped with Helios multilayer x-ray optics. Preliminary lattice constants were obtained with the Bruker program SMART.[8] Integrated reflection intensities were produced using the Bruker program SAINT.[9] Data sets were corrected empirically for variable absorption effects using equivalent reflections. The Bruker software package SHELXTL was used to solve the structure using "direct methods" techniques. All stages of weighted full-matrix least-squares refinement were conducted using Fo^2 data with the SHELXTL v2014 software package.[10]

The final structural model incorporated anisotropic thermal parameters for all nonhydrogen atoms and isotropic thermal parameters for all hydrogen atoms. Hydrogen atoms in the ^{6Me}dpaq ligands and acetonitrile molecules of crystallization were fixed at idealized riding model sp^2- or sp^3-hybridized positions with C-H bond lengths of 0.95 - 0.99 Å. Both hydrogen atoms for the water

molecule were located from a difference Fourier and included in the structural model as independent isotropic atoms whose parameters were allowed to refine in least-squares refinement cycles. All methyl groups were refined as idealized rigid rotors (with a C-H bond length of 0.98 Å) that were allowed to rotate freely about their C-C bonds in least-squares refinement cycles. The isotropic thermal parameters of idealized hydrogen atoms were fixed at values 1.2 (non-methyl) or 1.5 (methyl) times the equivalent isotropic thermal parameter of the carbon atom to which they are covalently bonded. The relevant crystallographic and structure refinement data for $[Mn^{II}(H_2O)(^{6Me}dpaq)](OTf)$ are given in Table S3.

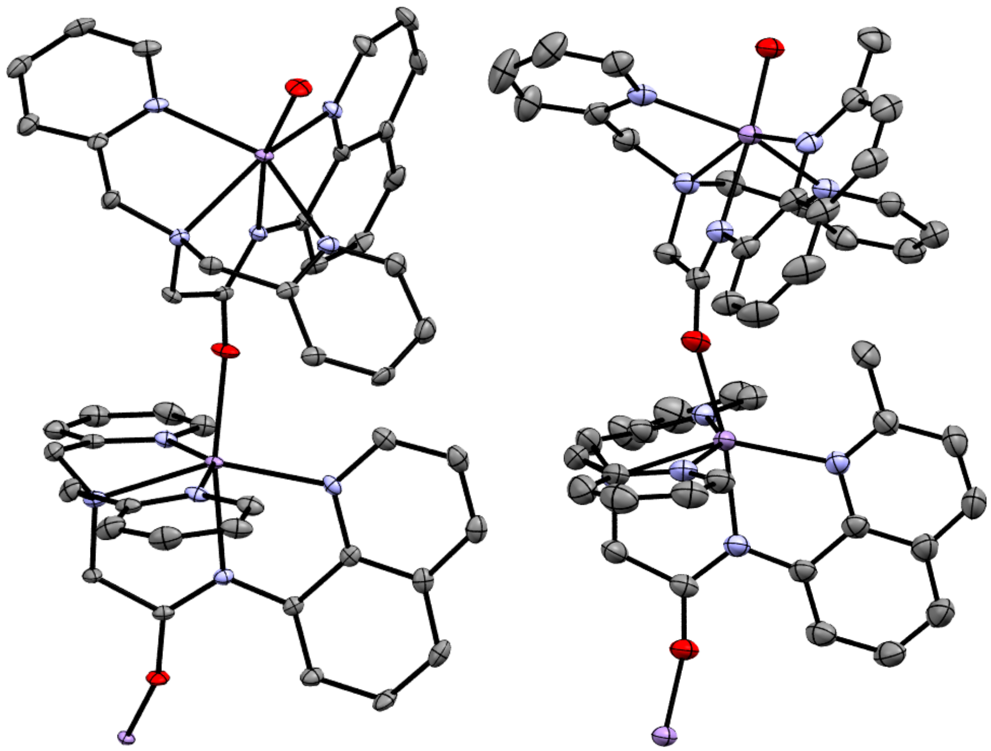

Fig. S4. Left: Polymeric structures of $[Mn^{II}(dpaq)](OTf)$[7] and $[Mn^{II}(dpaq^{2Me})](OTf)$[5] obtained by X-ray diffraction experiments described in ref. 7 and 4, respectively.[5]

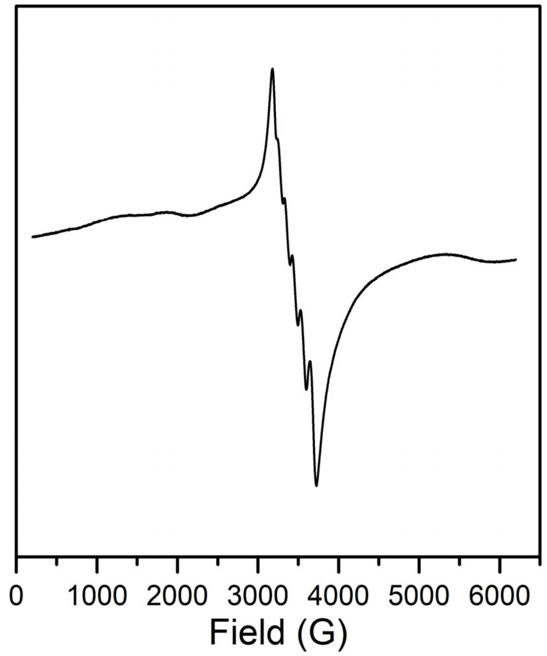

Fig. S5. EPR spectrum obtained for a 2 mM solution of $[Mn^{II}(H_2O)(^{6Me}dpaq)]OTf$ in CH_3CN showing a six-line signal consistent with the assignment of a mononuclear Mn^{II} species (g = 2.00, A = 93.9 G).

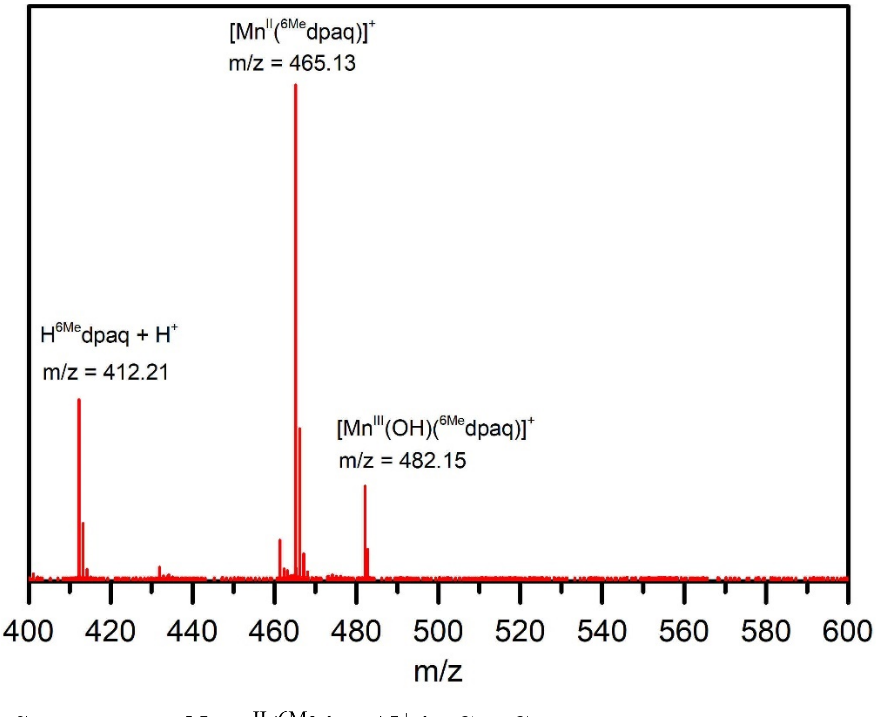

Fig. S6. ESI-MS spectrum of $[Mn^{II}(^{6Me}dpaq)]^+$ in CH_3CN.

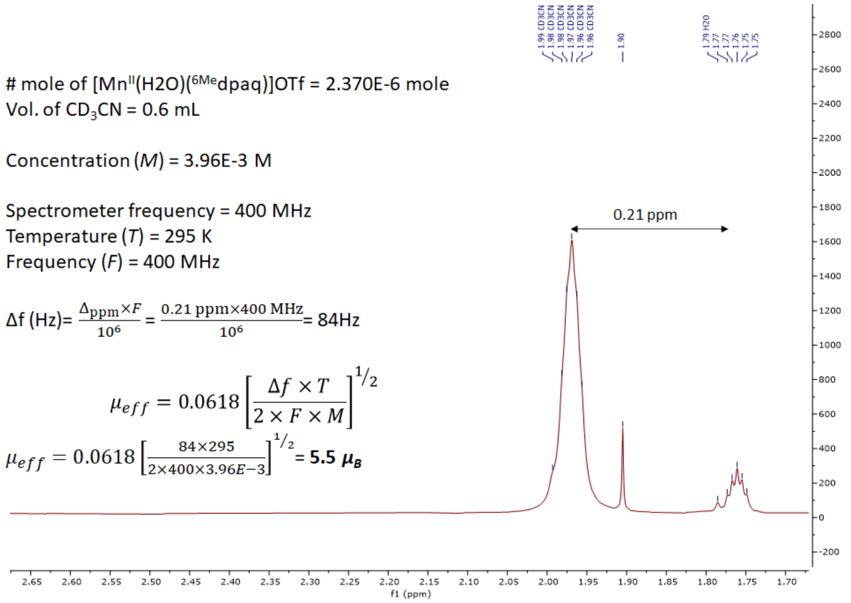

Fig. S7. Evans NMR of $[Mn^{II}(H_2O)(^{6Me}dpaq)]OTf$ in CD_3CN.

Synthesis and Characterization of [Mn^{III}(OH)(^{6Me}dpaq)](OTf). The [Mn^{II}(H_2O)(^{6Me}dpaq)]OTf complex reacts very slowly with dioxygen to produce [Mn^{III}(OH)(^{6Me}dpaq)]OTf (Fig. S8, left). A more ready method for obtaining [Mn^{III}(OH)(^{6Me}dpaq)]OTf was identified through oxidation of [Mn^{II}(H_2O)(^{6Me}dpaq)]OTf in CH_3CN using 0.5 equiv. PhIO (Fig. S8, right). The oxidation reaction led to a change in color from the bright orange color CH_3CN solution of [Mn^{II}(H_2O)(^{6Me}dpaq)]OTf to dark bronze color. Mass spectral analysis of CH_3CN solutions of [Mn^{III}(OH)(^{6Me}dpaq)](OTf) reveal a prominent peak at m/z = 482.14, in good agreement with the predicted m/z for [Mn^{III}(OH)(^{6Me}dpaq)]$^+$ (calculated m/z = 482.14, Fig. S9). The ESI-MS data also show a peak associated with the Na^+ cation of the H^{6Me}dpaq ligand (Fig. S9). The solution-phase magnetic moment of [Mn^{III}(OH)(^{6Me}dpaq)]$^+$, as determined using the Evans ^{1}H NMR method, support the assignment of this species as a high-spin Mn^{III} center (μ_{eff} = 4.9 μ_B, expected spin-only value of μ_{eff} = 4.90 μ_B, see Fig. S10). ^{1}H NMR characterization suggests that the dissolution of [Mn^{III}(OH)(^{6Me}dpaq)](OTf) in CD_3CN does not result in the formation of (μ-oxo)dimanganese(III, III) species.[8] Presumably the steric bulk of the additional 6-methyl moiety is sufficient to prohibit formation of a (μ-oxo)dimanganese(III, III) complex.

X-ray quality crystals of [Mn^{III}(OH)(^{6Me}dpaq)](OTf) were obtained by crystallization of the concentrated bronze color solution resulting from the oxidation of [Mn^{II}(H_2O)(^{6Me}dpaq)]OTf by slow vapor diffusion of diethyl ether into the CH_3CN solution at room temperature.

X-ray diffraction data collection and analysis for [Mn^{III}(OH)(^{6Me}dpaq)](OTf). A set of 4121 unique reflections were collected[9] for a 92/8 racemically-twinned crystal of [Mn($C_{25}H_{24}N_5O$)(OH)][O_3SCF_3] using 1.0°-wide ω- or ϕ-scan frames with scan times of 8-30 seconds and monochromated CuKα radiation (λ = 1.54178 Å) on a Bruker Proteum Single Crystal Diffraction System equipped with dual CCD area detectors. Data collection utilized a Platinum 135 CCD detector and Helios high-brilliance multilayer optics. X-rays were provided with a Bruker MicroStar microfocus Cu rotating anode x-ray source operating at 45 kV and 60 mA. The integrated data[10] were corrected empirically for variable absorption effects using equivalent reflections. The Bruker software package SHELXTL was used to solve the structure using "direct methods" techniques. All stages of weighted full-matrix least-squares refinement were conducted using F_o^2 data with the SHELXTL XL v2014 software package[11].

The asymmetric unit of [Mn($C_{25}H_{24}N_5O$)(OH)][O_3SCF_3] contains an ordered [Mn($C_{25}H_{24}N_5O$)(OH)]$^+$ cation and an ordered triflate anion. All nonhydrogen atoms of [Mn($C_{25}H_{24}N_5O$)(OH)][O_3SCF_3] were included in the structural model with anisotropic thermal parameters that were allowed to vary along with their positional parameters in least-squares refinement cycles. The hydrogen atom of the coordinated hydroxyl group was located from a difference Fourier and included in the structural model as an independent isotropic atom whose parameters was also allowed to vary. Methyl groups for [Mn($C_{25}H_{24}N_5O$)(OH)][O_3SCF_3] were incorporated into the structural model as idealized rigid rotors (using sp^3-hybridized geometry and a C–H bond length of 0.98 Å) that were permitted to rotate freely about their C–C bonds in least-squares refinement cycles. The remaining non-methyl hydrogen atoms for [Mn($C_{25}H_{24}N_5O$)(OH)][O_3SCF_3] were included in the structural model as idealized riding-model atoms (assuming sp^2- or sp^3-hybridization of the carbon atoms with C–H bond lengths of 0.95 or 0.99 Å). The isotropic thermal parameters of all idealized hydrogen atoms were fixed at values 1.2 (nonmethyl) or 1.5 (methyl) times the equivalent isotropic thermal parameter of the carbon atom to which they are covalently bonded.

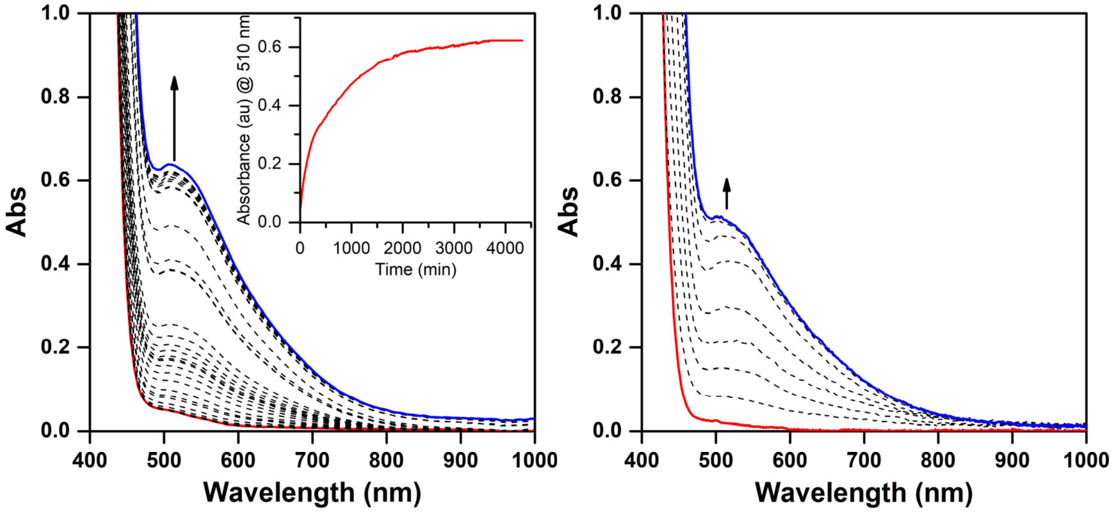

Fig. S8. Electronic absorption spectra monitoring the reaction of a 2.5 mM solution of [Mn^{II}(H_2O)(^{6Me}dpaq)]OTf (red trace) with dioxygen (left) and 2.0 mM solution of [Mn^{II}(H_2O)(^{6Me}dpaq)]OTf (red trace) with 0.5 equiv. PhIO in CH_3CN at 298 K (right). The dashed traces show the reaction progress, and the blue trace is the final spectrum.

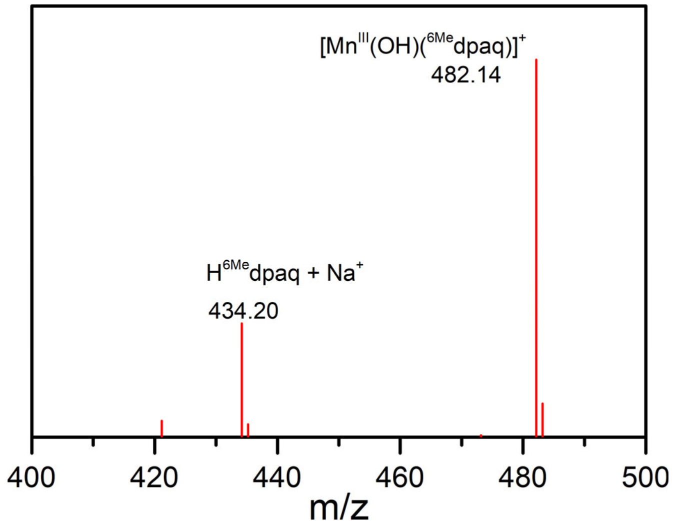

Fig. S9. ESI-MS of 0.002 mM $[Mn^{III}(OH)(^{6Me}dpaq)]^{+}$ in CH_3CN.

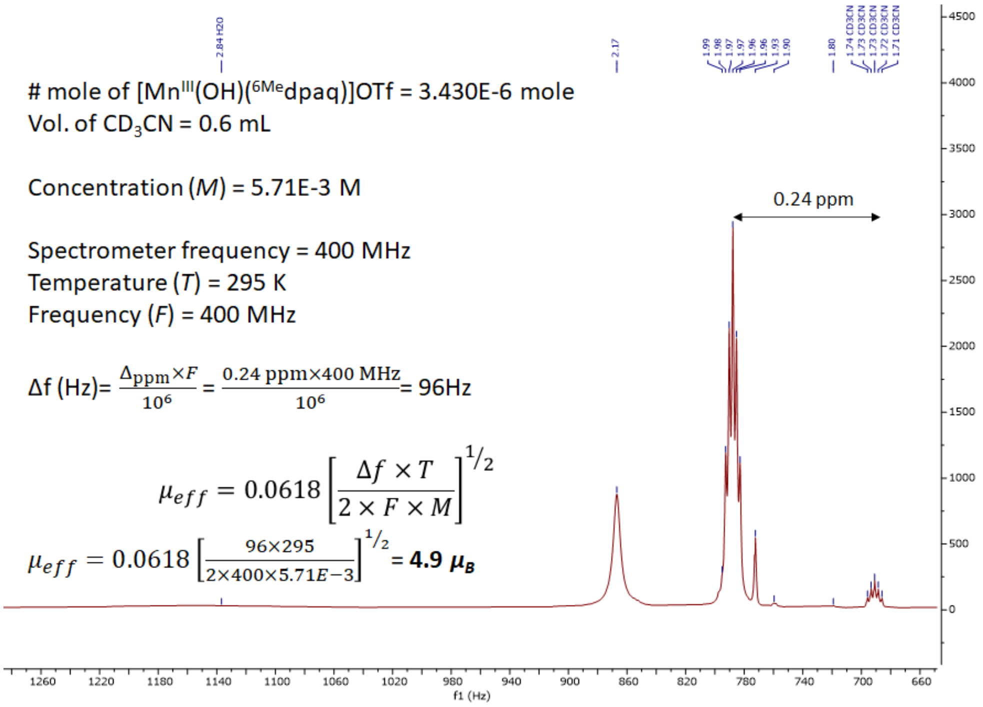

Fig. S10. Evans NMR of $[Mn^{III}(OH)(^{6Me}dpaq)]OTf$ in CD_3CN.

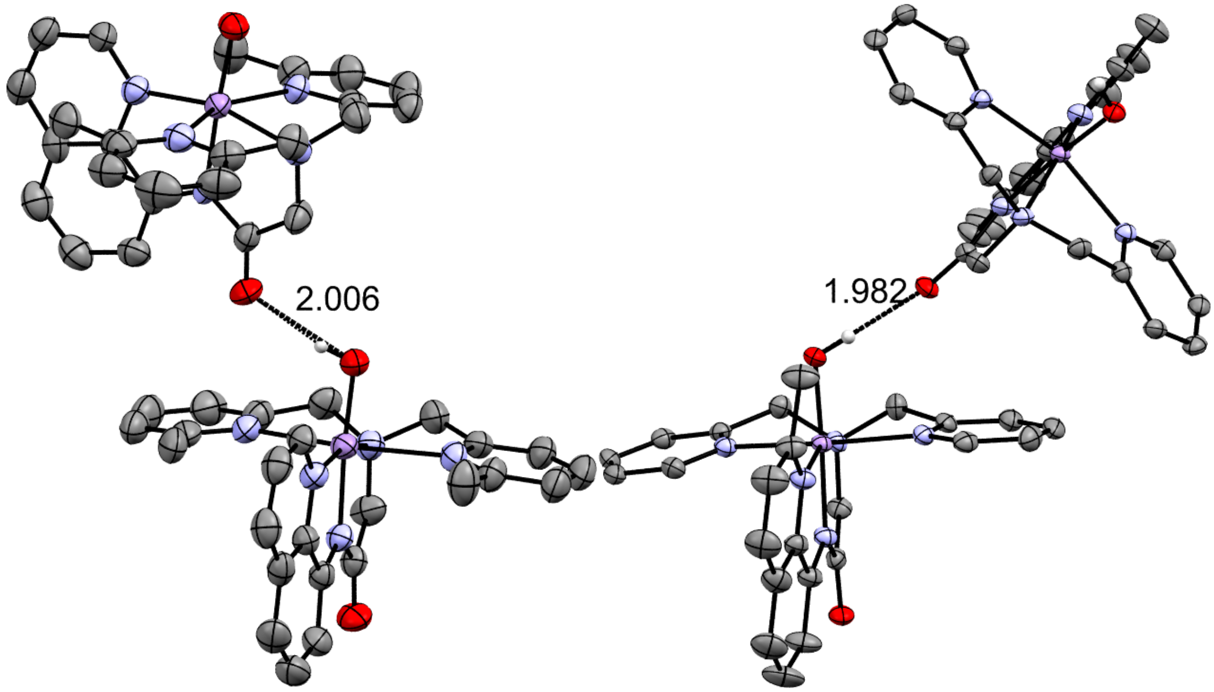

Fig. S11. ORTEP diagrams showing the hydrogen bonding interaction between two $[Mn^{III}(OH)(^{6Me}dpaq)]^+$ (left) and $[Mn^{III}(OH)(^{2Me}dpaq)]^+$ (right) molecules in the unit cell. Data for $[Mn^{III}(OH)(^{2Me}dpaq)]^+$ are described in reference [5].

Table S1. Manganese-ligand bond lengths (Å) from the crystal structures of $[Mn^{II}(OH_2)(^{6Me}dpaq)](OTf)$, $[Mn^{II}(dpaq)](OTf)$, $[Mn^{II}(dpaq^{2Me})](OTf)$, $[Mn^{III}(OH)(^{6Me}dpaq)](OTf)$, $[Mn^{III}(OH)(dpaq^{2Me})](OTf)$, $[Mn^{III}(OH)(dpaq^{2Me})](OTf)$, and $[Mn^{III}(OH)(dpaq^{Cl})](OTf)$.

	$[Mn^{II}(OH_2)(L)](OTf)$	$[Mn^{II}(L)](OTf)$		$[Mn^{III}(OH)(L)](OTf)$			
	L = ^{6Me}dpaq	L = dpaq	L= dpaq^{2Me}	L = ^{6Me}dpaq	L = dpaq	L = dpaq^{2Me}	L = dpaq^{5Cl}
Mn–O1	2.108(3)	2.079(2)	2.116(2)	1.806(6)	1.806(13)	1.819(3)	1.8067(18)
Mn–N1	2.233(3)	2.214(3)	2.268(3)	2.041(7)	2.072(14)	2.186(3)	2.066(2)
Mn– N2	2.152(4)	2.191(3)	2.172(3)	1.962(6)	1.975(14)	1.979(3)	1.9758(18)
Mn–N3	2.280(3)	2.314(3)	2.317(3)	2.130(6)	2.173(14)	2.303(3)	2.1668(19)
Mn–N4	2.354(4)	2.244(3)	2.275(3)	2.322(6)	2.260(14)	2.148(3)	2.245(2)
Mn–N5	2.417(3)	2.286(3)	2.286(3)	2.381(7)	2.216(15)	2.158(3)	2.218(2)

[a] Data for $[Mn^{II}(dpaq)](OTf)$ and $[Mn^{III}(OH)(dpaq)](OTf)$ are described in reference[7]. Data for $[Mn^{II}(dpaq^{2Me})](OTf)$ and $[Mn^{III}(OH)(dpaq^{2Me})](OTf)$ are described in reference[5]. Data for $[Mn^{III}(OH)(dpaq^{5Cl})](OTf)$ are described in reference[8].

Synthesis and Characterization of [Mn^{III}(OO^{t}Bu)(^{6Me}dpaq)]$^+$ and [Mn^{III}(OOCm)(^{6Me}dpaq)]$^+$. [Mn^{III}(OO^{t}Bu)(^{6Me}dpaq)]$^+$ and [Mn^{III}(OOCm)(^{6Me}dpaq)]$^+$ were prepared by the reaction of [Mn^{II}(H_2O)(^{6Me}dpaq)]OTf in CH_3CN with 1.5 equiv. of ^{t}BuOOH and CmOOH, respectively, at 298 K. The formation of [Mn^{III}(OO^{t}Bu)(^{6Me}dpaq)]$^+$ and [Mn^{III}(OOCm)(^{6Me}dpaq)]$^+$ were monitored by electronic absorption spectroscopy. These data show the appearance of an electronic absorption feature at around 650 nm. This feature increased in intensity with time, and the formation was deemed complete after there was no change in intensity with time. The formation of [Mn^{III}(OO^{t}Bu)(^{6Me}dpaq)]$^+$ and [Mn^{III}(OOCm)(^{6Me}dpaq)]$^+$ was also performed by the reaction of [Mn^{III}(OH)(^{6Me}dpaq)]$^+$ in CH_3CN with 1.0 equiv. ^{t}BuOOH and CmOOH, respectively, at 298 K. In thi case, the formation was evident by the decrease in the intensity of the 510 nm feature of [Mn^{III}(OH)(^{6Me}dpaq)]$^+$ and the appearance of a 650 nm feature. The formation was deemed complete after there was no change in intensity of the 650 nm feature with time. Mass spectral analysis of a CH_3CN solution of [Mn^{III}(OO^{t}Bu)(^{6Me}dpaq)]$^+$ shows a peak at m/z = 554.19 that is consistent with the formulation of this species as [Mn^{III}(OO^{t}Bu)(^{6Me}dpaq)]$^+$ (calculated m/z = 554.20, Fig. S14, left). The magnetic moment of [Mn^{III}(OO^{t}Bu)(^{6Me}dpaq)]$^+$, as determined using the Evans ^{1}H NMR method, supports the assignment of this species as a high-spin Mn^{III} center (μ_{eff} = 4.8 μ_B, which compares well with the free-ion value for an S = 2 system of 4.90 μ_B). ESI-MS data collected for [Mn^{III}(OOCm)(^{6Me}dpaq)]$^+$ show a characteristic peak at m/z = 616.18 that agrees with that expected for this complex (calculated m/z = 616.21, Fig. S14, right). X-ray quality crystals of the [Mn^{III}(OOCm)(^{6Me}dpaq)]$^+$ were obtained by layering of concentrated CH_3CN solution of [Mn^{III}(OOCm)(^{6Me}dpaq)]$^+$ with cold diethyl ether. The set-up was kept in a freezer at 233 K and green crystalline material used for X-ray crystallography studies were obtained after 2 days.

X-ray diffraction data collection and analysis for [Mn^{III}(OOCm)(^{6Me}dpaq)](OTf). Complete sets of unique reflections were collected with monochromated CuKα radiation for a crystal sample of the [Mn^{III}(OOCm)(^{6Me}dpaq)](OTf) with single domain. Totals of 4998 1.0°-wide ω- or ϕ-scan frames with counting times of 10-15 seconds were collected for [Mn^{III}(OOCm)(^{6Me}dpaq)](OTf) with a Bruker APEX II CCD area detector. X-rays were provided by a Bruker MicroStar microfocus rotating anode operating at 45kV and 60 mA and equipped with Helios multilayer x-

ray optics. Preliminary lattice constants were obtained with the Bruker program SMART.[8] Integrated reflection intensities were produced using the Bruker program SAINT.[9] Data sets were corrected empirically for variable absorption effects using equivalent reflections. The Bruker software package SHELXTL was used to solve the structure using "direct methods" techniques. All stages of weighted full-matrix least-squares refinement were conducted using Fo^2 data with the SHELXTL v2014 software package.[10]

The final structural model incorporated anisotropic thermal parameters for all nonhydrogen atoms and isotropic thermal parameters for all hydrogen atoms. Hydrogen atoms in the ^{6Me}dpaq ligands and acetonitrile molecules of crystallization were fixed at idealized riding model sp^2- or sp^3-hybridized positions with C-H bond lengths of 0.95 - 0.99 Å. All methyl groups were refined as idealized rigid rotors (with a C-H bond length of 0.98 Å) that were allowed to rotate freely about their C-C bonds in least-squares refinement cycles. The isotropic thermal parameters of idealized hydrogen atoms were fixed at values 1.2 (non-methyl) or 1.5 (methyl) times the equivalent isotropic thermal parameter of the carbon atom to which they are covalently bonded. The relevant crystallographic and structure refinement data for $[Mn^{III}(OOCm)(^{6Me}dpaq)](OTf)$ are given in Table S3.

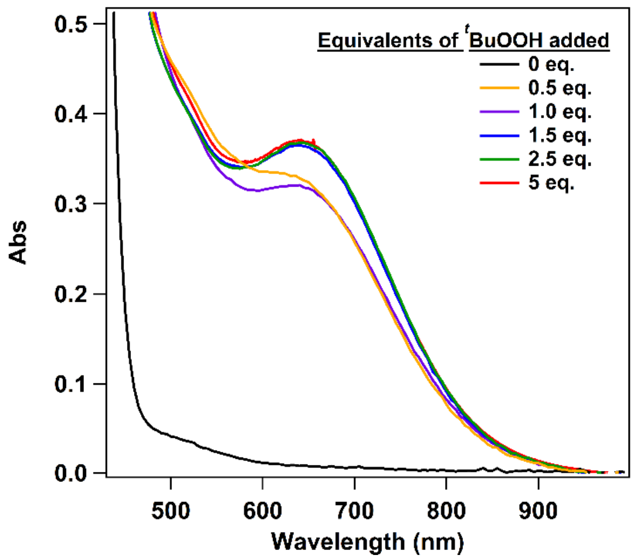

Fig. S12. Electronic absorption spectra obtained upon the addition of aliquots of ^{t}BuOOH to a 2 mM solution of $[Mn^{II}(H_2O)(^{6Me}dpaq)]OTf$ in CH_3CN at 298 K. Full formation of $[Mn^{III}(OO^tBu)(^{6Me}dpaq)]^+$ (λ_{max} = 650 nm) is achieved upon the addition of 1.5 equiv. of ^{t}BuOOH.

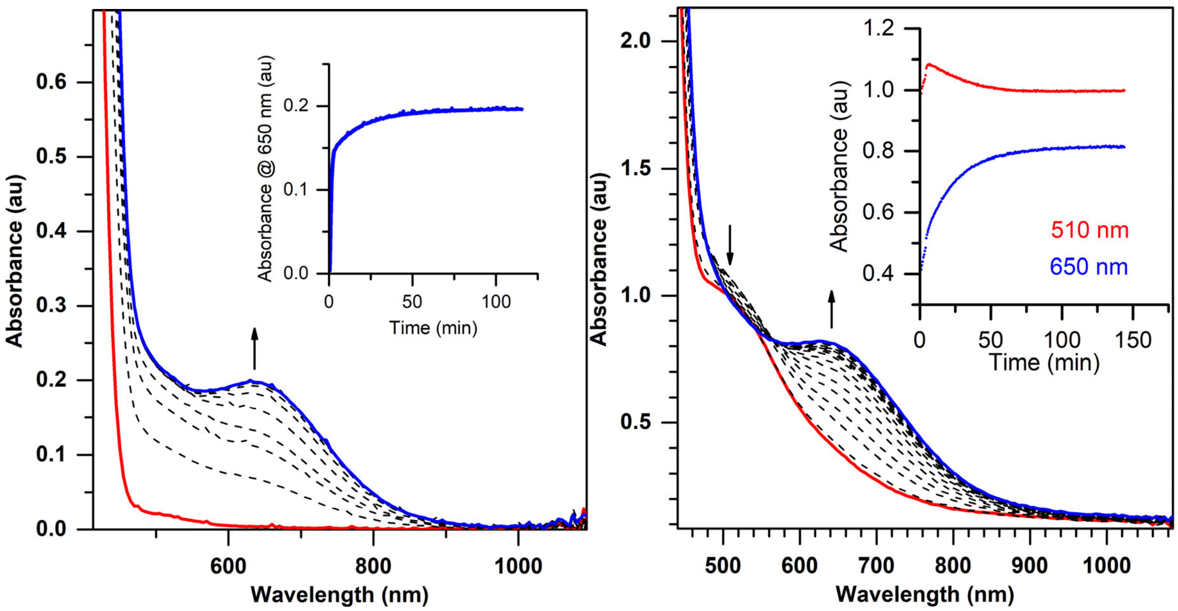

Fig. S13. Left: Electronic absorption spectra showing the formation of the green $[Mn^{III}(OOCm)(^{6Me}dpaq)]^+$ species (blue trace) from the oxidation of 1.0 mM $[Mn^{II}(H_2O)(^{6Me}dpaq)]OTf$ (red trace) with 1.5 equiv. CmOOH. Right: Electronic absorption spectra showing the formation of $[Mn^{III}(OOCm)(^{6Me}dpaq)]^+$ from the reaction of 3.0 mM $[Mn^{III}(OH)(^{6Me}dpaq)]^+$ (red trace) with 1.0 equiv. CmOOH (blue trace is the final spectrum). Time courses for each reaction are shown in the insets.

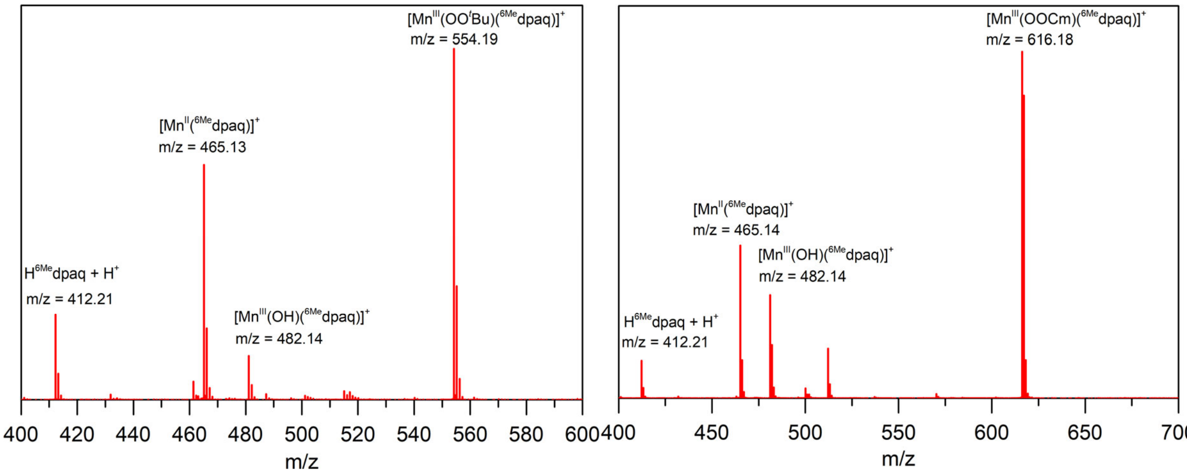

Fig. S14. ESI-MS of 0.002 mM $[Mn^{III}(OO^tBu)(^{6Me}dpaq)]^+$ (left) and $[Mn^{III}(OOCm)(^{6Me}dpaq)]^+$ (right) in CH_3CN.

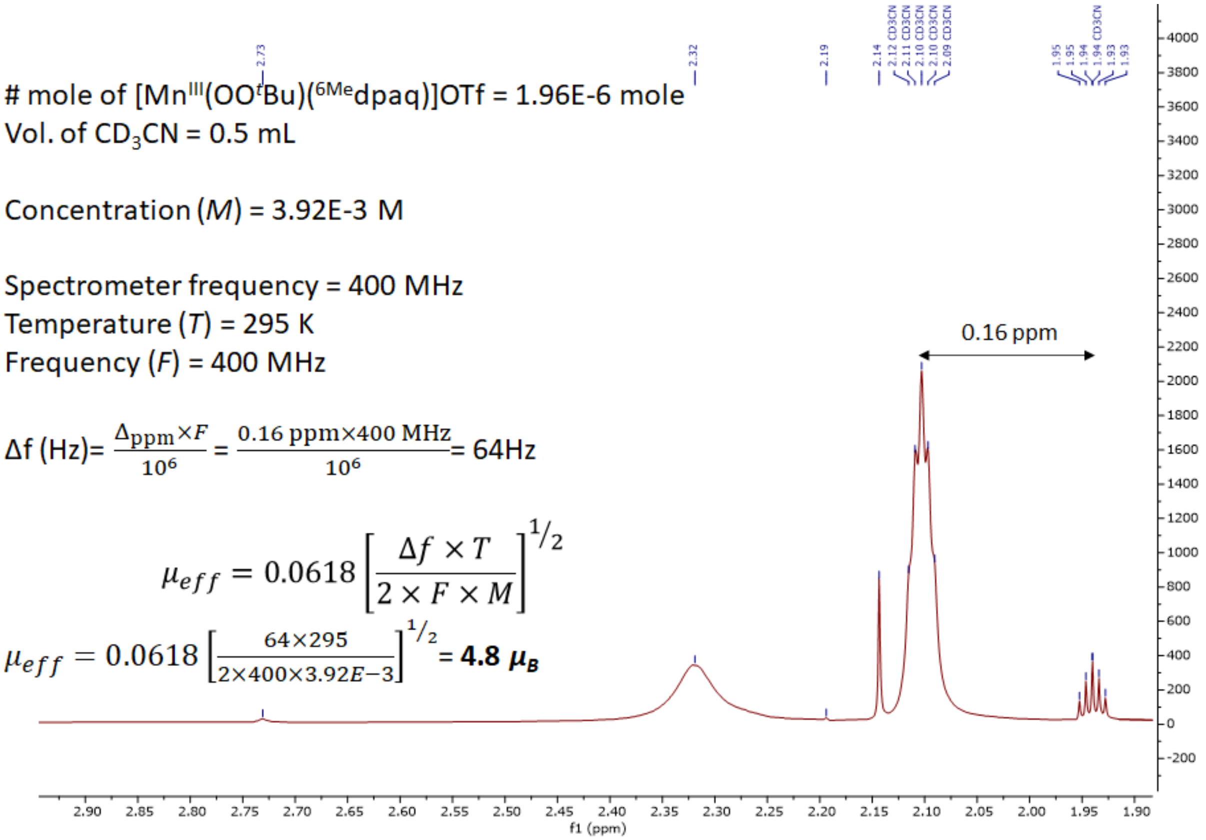

Fig. S15. Evans NMR Data for [Mn^{III}(OO^{t}Bu)(^{6Me}dpaq)]OTf in CD_3CN.

mole of [Mn^{III}(OOCm)(^{6Me}dpaq)]OTf = 3.14E-6 mole
Vol. of CD_3CN = 0.5 mL

Concentration (M) = 6.27E-3 M

Spectrometer frequency = 400 MHz
Temperature (T) = 295 K
Frequency (F) = 400 MHz

$$\Delta f\text{ (Hz)} = \frac{\Delta_{ppm} \times F}{10^6} = \frac{0.26\text{ ppm} \times 400\text{ MHz}}{10^6} = 104\text{Hz}$$

$$\mu_{eff} = 0.0618\left[\frac{\Delta f \times T}{2 \times F \times M}\right]^{1/2}$$

$$\mu_{eff} = 0.0618\left[\frac{104 \times 295}{2 \times 400 \times 6.27E-3}\right]^{1/2} = \mathbf{4.8}\ \boldsymbol{\mu_B}$$

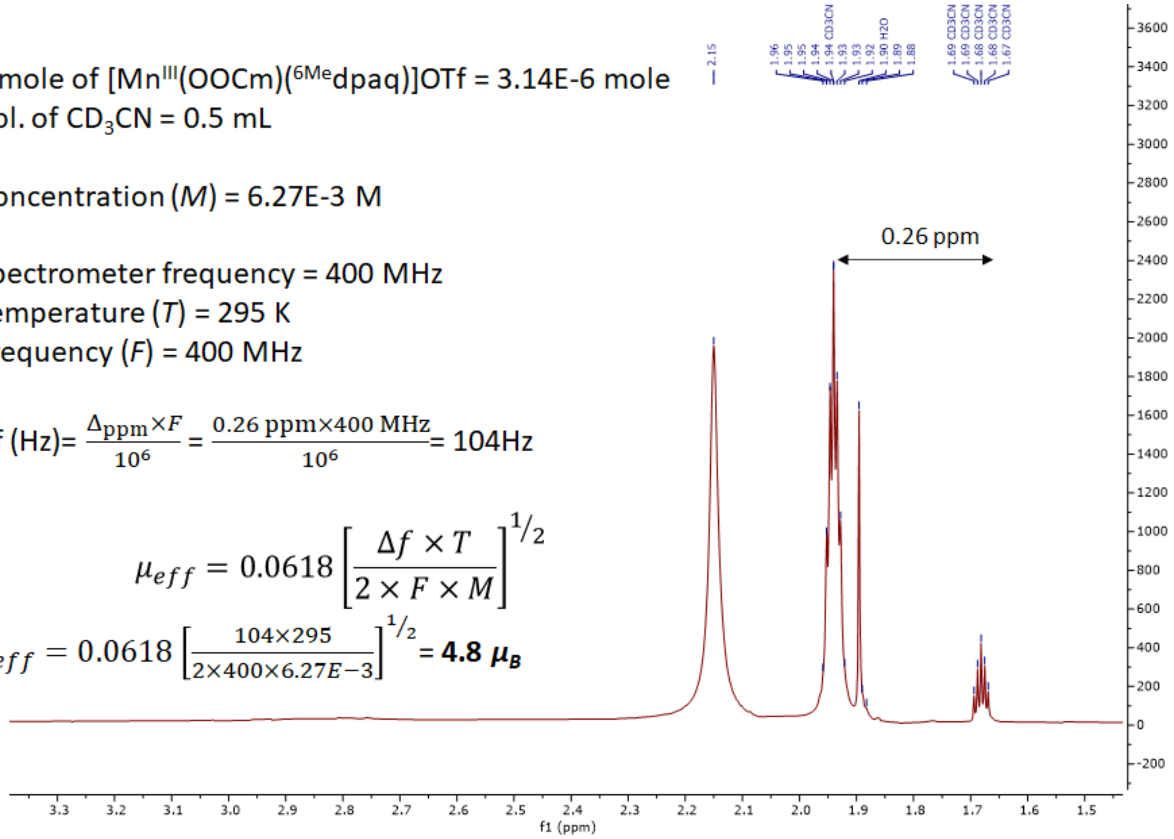

Fig. S16. Evans NMR Data for [Mn^{III}(OOCm)(^{6Me}dpaq)]OTf in CD_3CN.

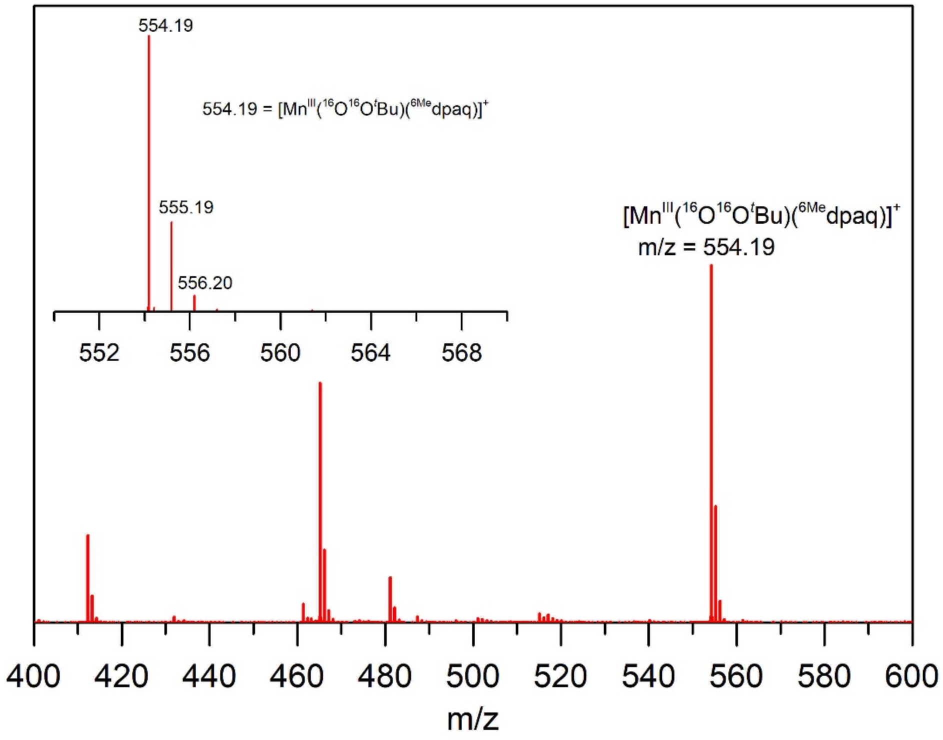

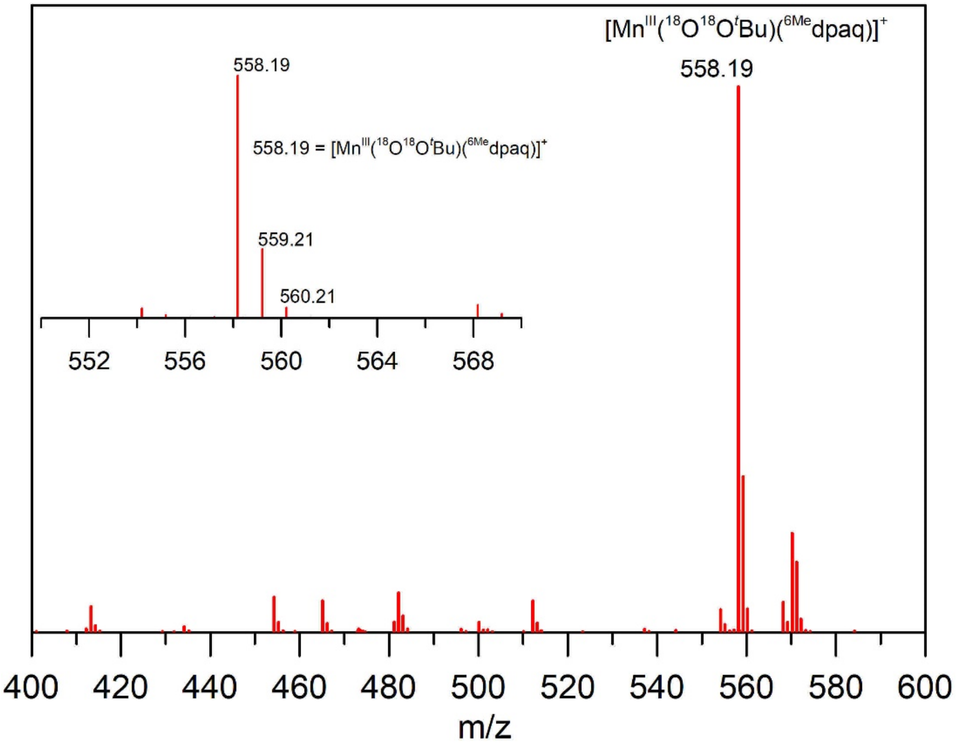

Fig. S17. ESI-MS data for $[Mn^{III}(^{16}O^{16}O^{t}Bu)(^{6Me}dpaq)]^{+}$ (top) and $[Mn^{III}(^{18}O^{18}O^{t}Bu)(^{6Me}dpaq)]^{+}$ (bottom) in MeCN.

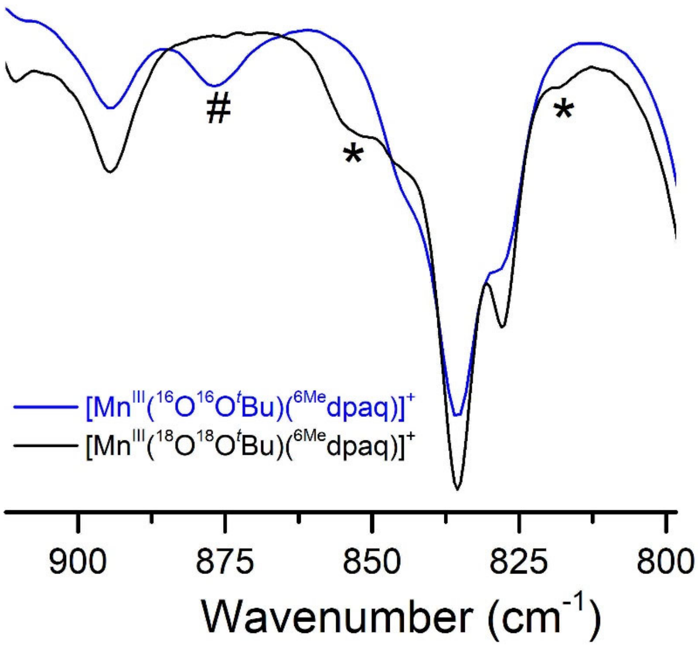

Fig. S18. IR spectra for $[Mn^{III}(^{16}O^{16}O^{t}Bu)(^{6Me}dpaq)]^{+}$ (blue trace) and $[Mn^{III}(^{18}O^{18}O^{t}Bu)(^{6Me}dpaq)]^{+}$ (black trace). The hash (#) and the asterisk (*) symbols represent missing and new features respectively in the spectrum of $[Mn^{III}(^{18}O^{18}O^{t}Bu)(^{6Me}dpaq)]^{+}$ relative to $[Mn^{III}(^{16}O^{16}O^{t}Bu)(^{6Me}dpaq)]^{+}$.

Table S2. Electronic Absorption Band Maxima (nm), Selected Bond Lengths (Å) and Angles (°), and O–O Stretching Frequencies ($\nu_{O\text{-}O}$, cm^{-1}) for Mn^{III}-alkylperoxo Complexes.

complex	λ		Mn-O	O-O	Mn-O-O	Mn-N[a]	$\nu_{O\text{-}O}$	Ref.
$[Mn^{III}(OO^{t}Bu)(^{6Me}dpaq)]^{+}$	500	650					877	[b]
$[Mn^{III}(OOCm)(^{6Me}dpaq)]^{+}$	500	650	1.849(3)	1.466(4)	110.4(2)	2.339	861	[b]
$[Mn^{III}(OO^{t}Bu)(dpaq)]^{+}$	475	710					872	11
$[Mn^{III}(OO^{t}Bu)(dpaq^{2Me})]^{+}$	475	690					NR	11
$[Mn^{III}(OO^{t}Bu)(S^{Me2}N_4(6\text{-Me-DPPN}))]^{+}$	420	585	1.843(3)	1.431(5)	124.2(3)	2.511	893	10
$[Mn^{III}(OO^{t}Bu)(S^{Me2}N_4(\text{QuinoPN}))]^{+}$	415	590	1.840(4)	1.438(5)	121.1(3)	2.484	895	10
$[Mn^{III}(OO^{t}Bu)(S^{Me2}N_4(\text{QuinoEN}))]^{+}$	385	590	1.861(5)	1.457(7)	109.2(4)	2.436	888	9, 10
$[Mn^{III}(OO^{t}Bu)(S^{Me2}N_4(6\text{-Me-DPEN}))]^{+}$	355	600	1.853(6)	1.468(7)	112.4(4)	2.413	875	10

[a] Mn–N bond length for the elongated bonds associated with the 6-Me-pyridyl or quinolinyl donors. [b] From this work.

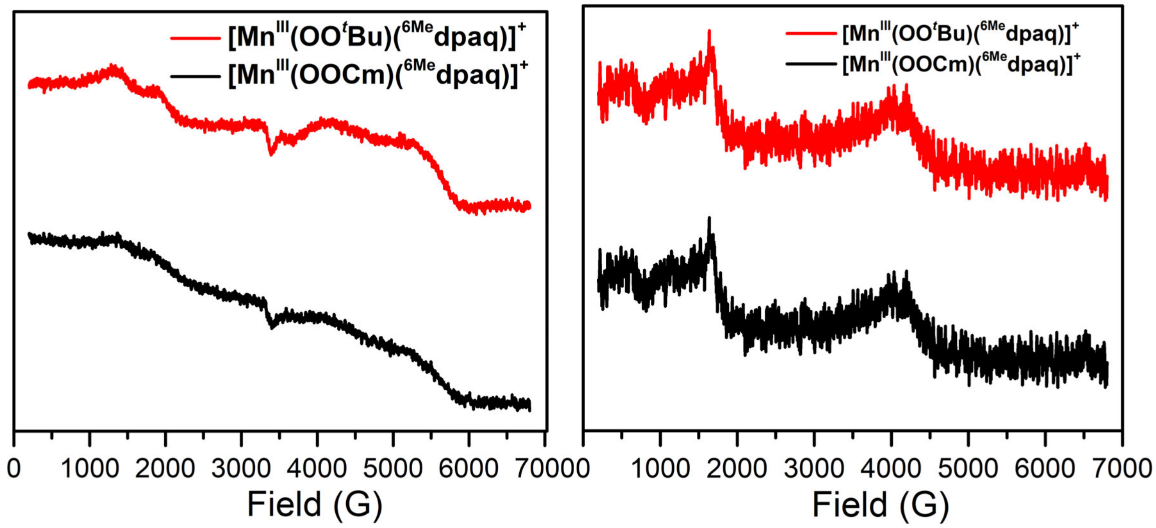

Fig. S19. X-band EPR spectra of frozen 5m M acetonitrile solutions of $[Mn^{III}(OO^tBu)(^{6Me}dpaq)]^+$ and $[Mn^{III}(OOCm)(^{6Me}dpaq)]^+$ at 10 K in perpendicular-mode (left) and parallel-mode (right).

Decay kinetics. 1.25 mM sample solutions of the $[Mn^{II}(OOR)(^{6Me}dpaq)]^+$ complexes (R = ^{t}Bu and Cm) in CH_3CN were prepared in the glovebox, dispensed into a quartz cuvette and covered with a rubber septum. The septum was wrapped with Parafilm. The cuvette was taken out of the glovebox, and the decay kinetics were monitored on a Varian Cary 50 Bio UV−visible spectrophotometer equipped with a temperature controller and stirrer.

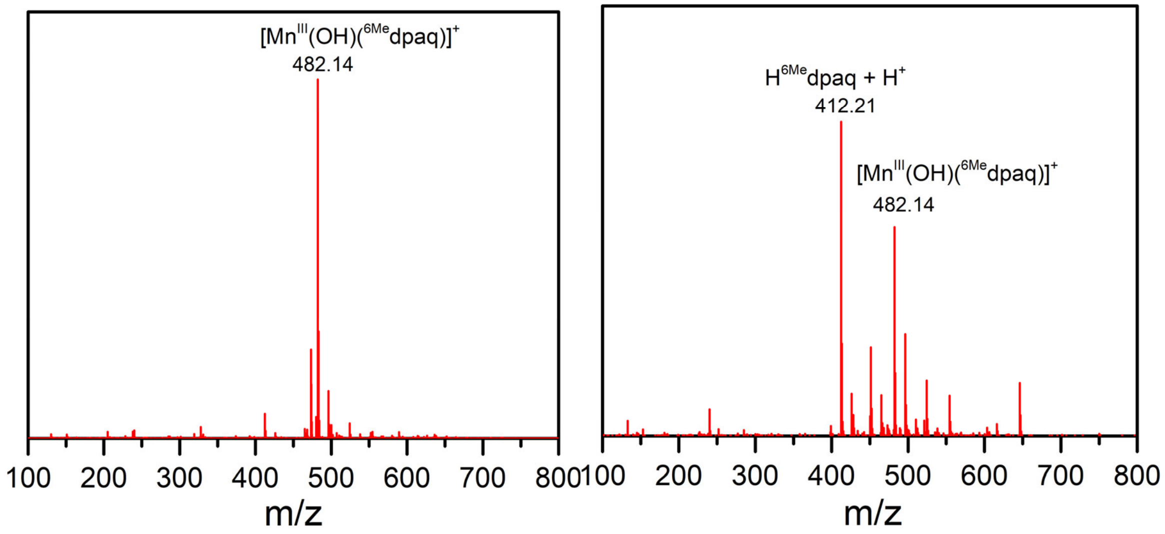

Fig. S20. ESI-MS data for $[Mn^{III}(OO^tBu)(^{6Me}dpaq)]^+$ decay product (left) and $[Mn^{III}(OOCm)(^{6Me}dpaq)]^+$ decay product (right) in CH_3CN.

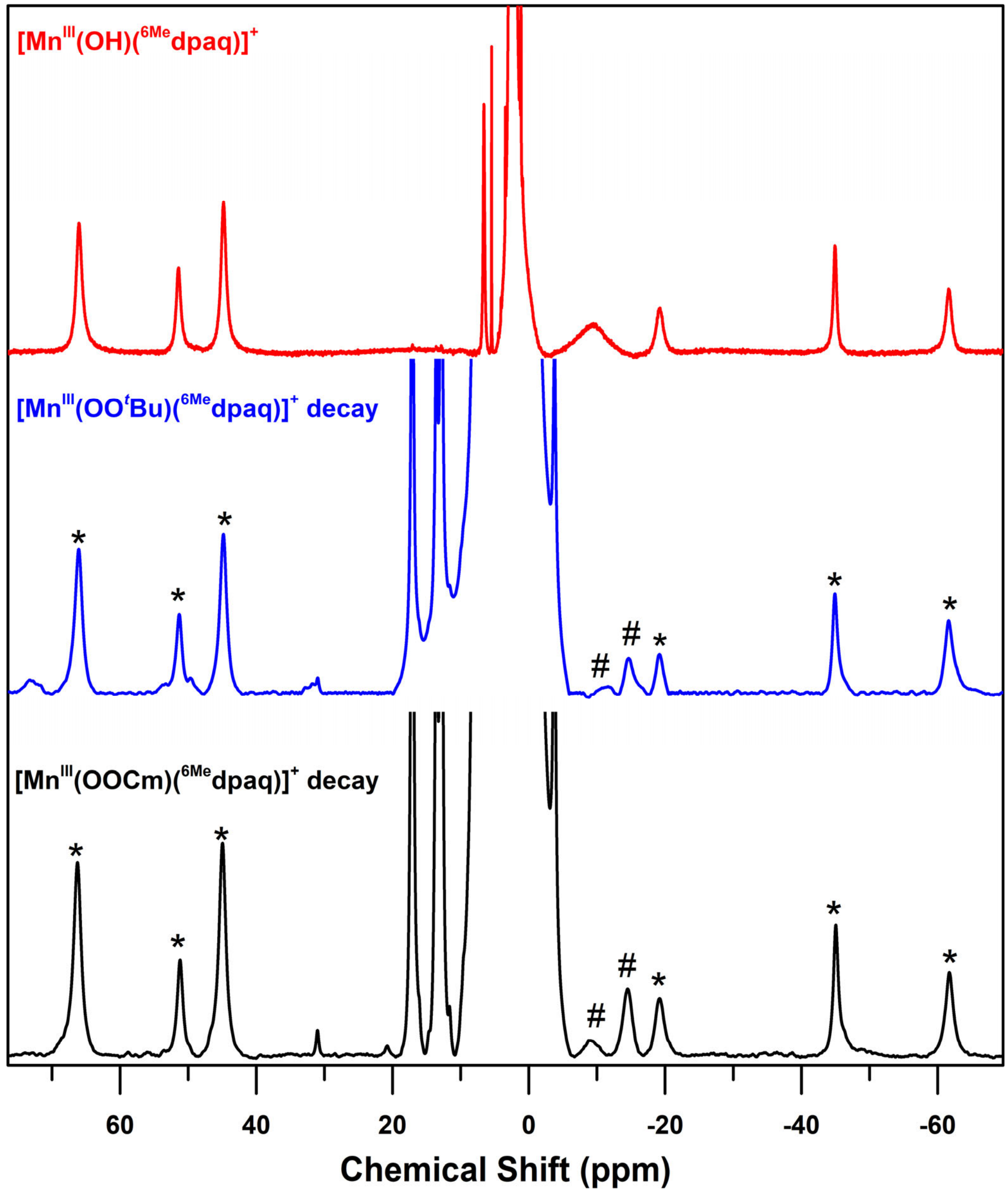

Fig. S21. ^{1}H NMR spectra of 20 mM solutions of $[Mn^{III}(OH)(^{6Me}dpaq)]^{+}$ (red) and the decay products from 12 mM solution $[Mn^{III}(OO^{t}Bu)(^{6Me}dpaq)]^{+}$ (blue), and 12 mM solution of $[Mn^{III}(OOCm)(^{6Me}dpaq)]^{+}$ (black). All samples were prepared in CD_3CN at 298 K. The asterisk marks peaks found in $[Mn^{III}(OH)(^{6Me}dpaq)]^{+}$. The hashtag marks peaks that could result from the resolution of the broad peak found at -9.6 ppm in $[Mn^{III}(OH)(^{6Me}dpaq)]^{+}$.

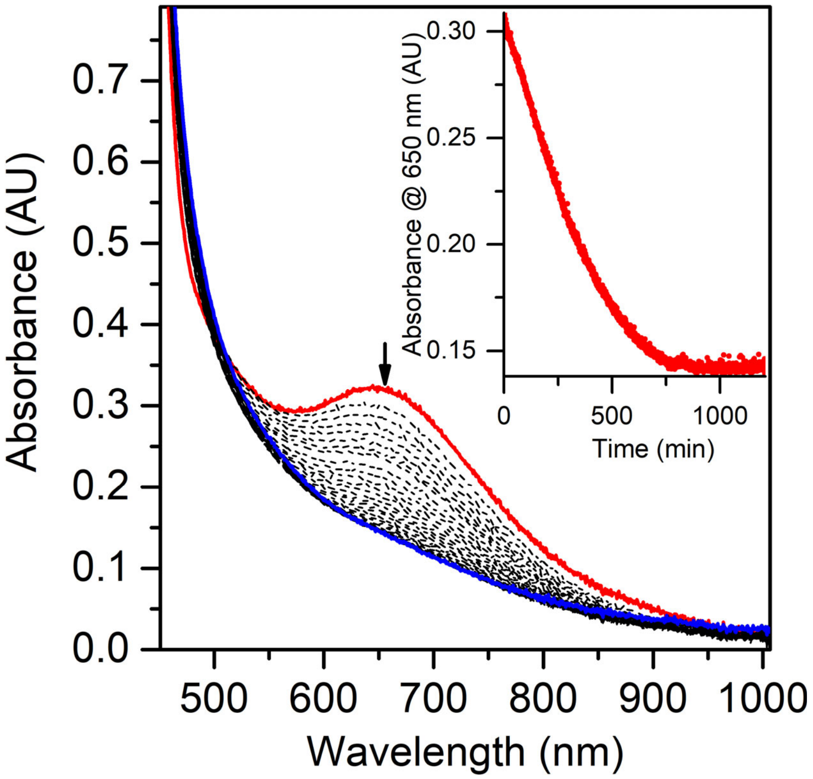

Fig. S22. Electronic absorption spectra showing the decay of anerobic CH_3CN solutions of 1.5 mM crude $[Mn^{III}(OOCm)(^{6Me}dpaq)]^+$ at 323K.

PPh_3 reaction kinetics and Eyring analysis. Samples of the $[Mn^{III}(OOR)(^{6Me}dpaq)]^+$ complexes in CH_3CN were prepared in the glovebox and dispensed into a quartz cuvette, covered with a rubber septum and wrapped with Parafilm. 300 μL of dichloromethane was added to an amount of PPh_3 in a 4.0 mL vial. The vial was covered with a rubber septum and wrapped with Parafilm. The cuvette and the vial containing the substrate were taken outside the glovebox. The cuvette was placed on the UV-vis spectrometer and equilibrated at 298 K for 10 minutes before the PPh_3 solution was added using a gastight syringe that was purged five times with nitrogen gas. For variable temperature kinetic experiments, the same procedure was repeated using 1.25 mM $[Mn^{III}(OOR)(^{6Me}dpaq)]^+$ and 100 equiv. PPh_3 in the temperature range of 288 - 313 K.

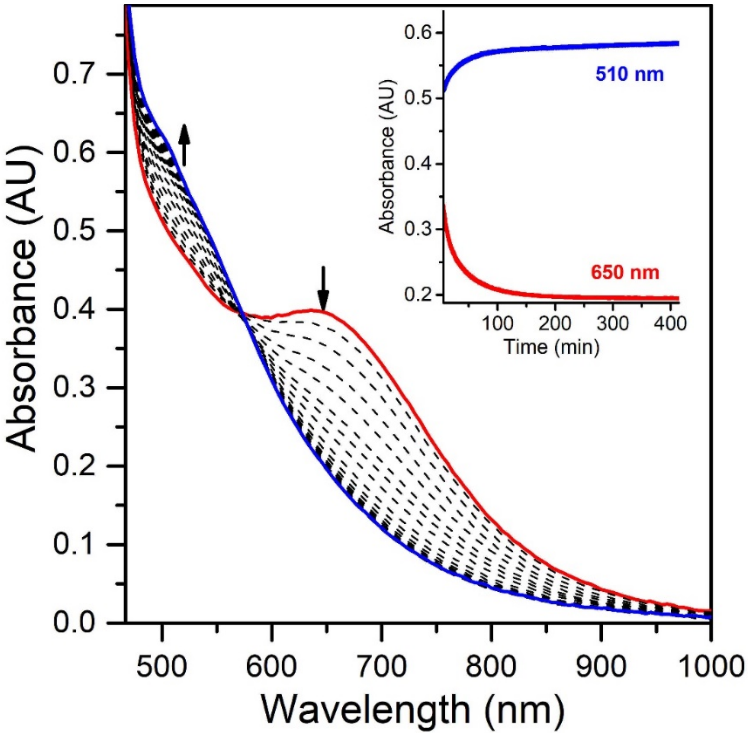

Fig. S23. Anaerobic decay of 1.5 mM $[Mn^{III}(OOCm)(^{6Me}dpaq)]^{+}$ in Benzonitrile at 323 K.

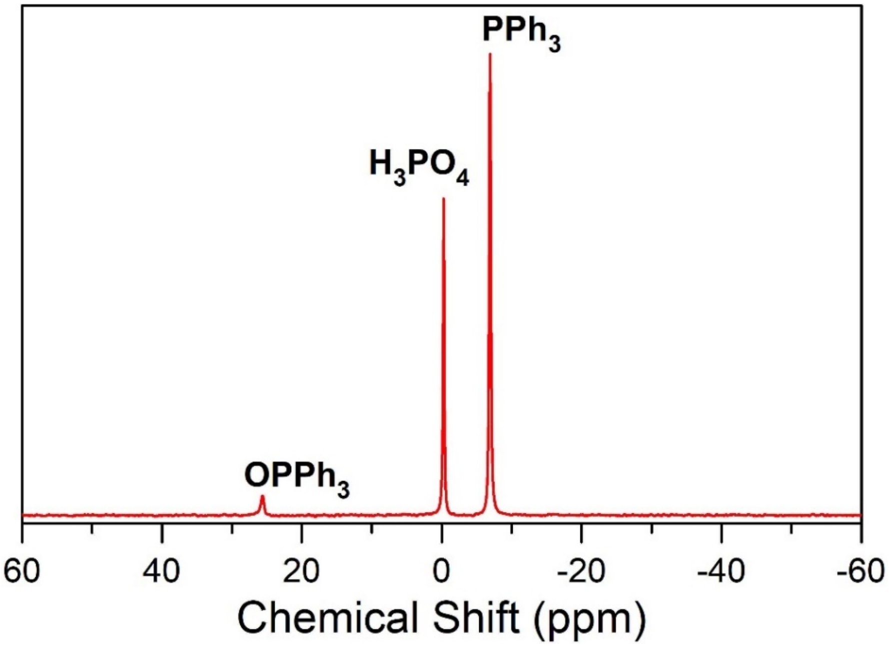

Fig. S24. ^{31}P NMR analysis of the products of the reaction of 10 equiv. of PPh_3 with $[Mn^{III}(OO^tBu)(^{6Me}dpaq)]^{+}$ in CH_3CN using H_3PO_4 as internal standard at 298 K.

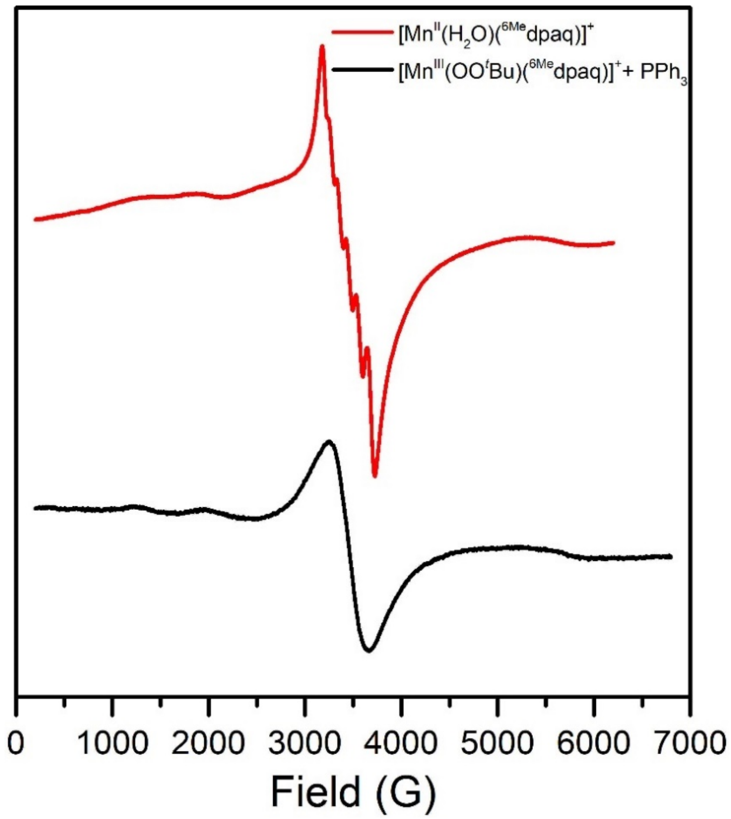

Fig. S25. 10 K, perpendicular-mode X-band EPR spectrum of the frozen CH_3CN solution following the reaction of 2 mM $[Mn^{III}(OO^tBu)(^{6Me}dpaq)]^+$ with 100 equiv. PPh_3 at 298 K (black trace). The 10 K EPR spectrum of $[Mn^{II}(OH_2)(^{6Me}dpaq)]^+$ in MeCN is included for comparison.

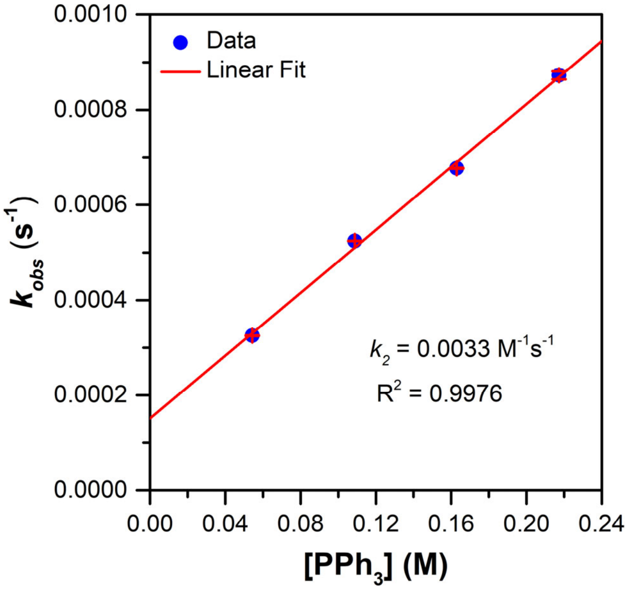

Fig. S26. Pseudo-first-order rate constants, k_{obs} (s^{-1}), versus PPh_3 concentration for a 1.0 mM CH_3CN solution of [Mn^{III}(OOCm)(^{6Me}dpaq)]$^+$ at 298 K.

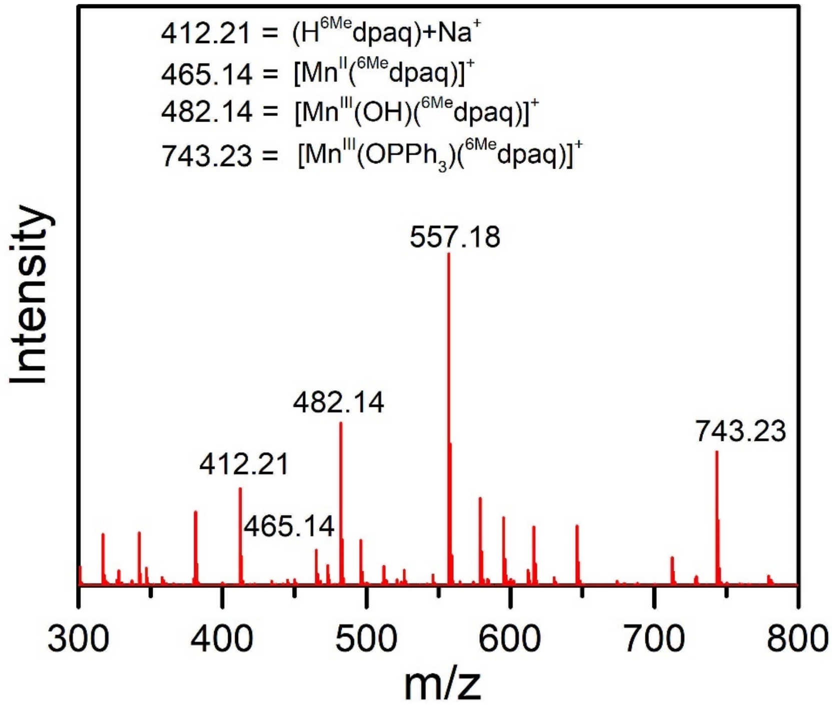

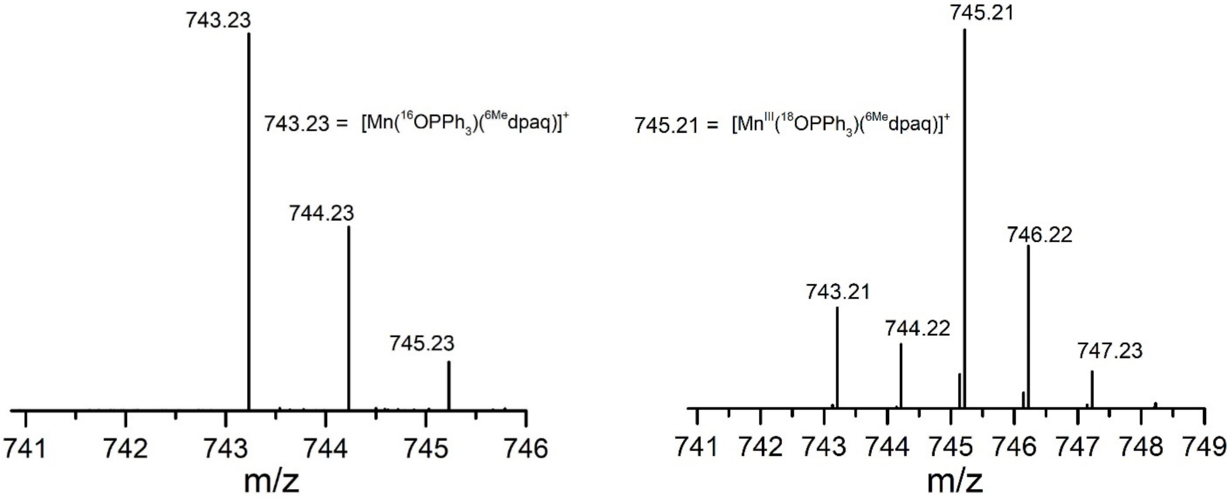

Fig. S27. ESI-MS data for the reaction of 22 mM of $[Mn^{III}(OOCm)(^{6Me}dpaq)]^+$ with 5 equiv. of PPh_3 at 298 K (top). Bottom: Expanded view of the spectral region for the $[Mn(OPPh_3)(^{6Me}dpaq)]^+$ complex for the Mn^{III}-alkylperoxo adduct prepared using $^{t}Bu^{16}O^{16}OH$ (left) and $^{t}Bu^{18}O^{18}OH$ (right).

Reactivity of $[Mn^{III}(OO^tBu)(^{6Me}dpaq)]^+$ and $[Mn^{III}(OOCm)(^{6Me}dpaq)]^+$ with DHA. The reaction of $[Mn^{III}(OO^tBu)(^{6Me}dpaq)]^+$ with 9,10-dihydroanthracene (DHA) was performed by adding 100 equiv. DHA, dissolved in 100 µL of CH_2Cl_2 to a cuvette containing a 1.75 mM solution of $[Mn^{III}(OO^tBu)(^{6Me}dpaq)]^+$ in CH_3CN in the glovebox. The cuvette was sealed with a rubber septum and wrapped with Parafilm. The reaction mixture was brought out of the glovebox and the reaction was heated to 323 K while monitoring the reaction by electronic absorption spectroscopy. After the reaction was completed, evident by the disappearance of the 650 nm feature, the reaction mixture was passed through a 2-inch silica plug and the eluate was dried in vacuo. The solid residue was analyzed by ^1H NMR spectroscopy for characterization and quantification. Quantification was performed with 1,4-benzoquinone as an internal standard (Fig. S30). At first, quantification showed 2.4 equiv. DHA converted to anthracene. However, control experiments without the $[Mn^{III}(OO^tBu)(^{6Me}dpaq)]^+$ also showed the conversion of DHA to anthracene (Fig. S32). Another control experiment was conducted where UV-light from the spectrometer was isolated from a reaction solution. In this case, the control experiment lacking $[Mn^{III}(OO^tBu)(^{6Me}dpaq)]^+$ revealed no oxidation of DHA. This result revealed that UV-light from the spectrometer contributes to the observed 2.4 equiv. conversion of DHA to anthracene. In subsequent procedures, we isolate the reaction mixture from the interference from the UV-light and quenched the reaction after 7 hours. These experiments revealed that 1.4 equiv. DHA were converted to anthracene. A similar reaction performed at 298 K over the course of 13 hours revealed only trace amounts of anthracene. Under these conditions, the final solution was still green, indicating the lack of full consumption of $[Mn^{III}(OO^tBu)(^{6Me}dpaq)]^+$. Similar conditions were used to explore the reaction of $[Mn^{III}(OOCm)(^{6Me}dpaq)]^+$ with DHA. In this reaction, we also found *ca.* 1.4 equiv. DHA converted to anthracene. The decay rate of $[Mn^{III}(OOCm)(^{6Me}dpaq)]^+$ was 0.0188 min^{-1}, which is comparable to that of $[Mn^{III}(OO^tBu)(^{6Me}dpaq)]^+$ under the same conditions (0.0141 min^{-1}). These decay rates are indistinguishable from the thermal decays rates of these complexes. The presence of O_2 also had an effect on the product distribution. Aerobic studies of this reaction gave mixtures of products, including anthracene and the oxygenated products anthraquinone and anthrone. The same experimental procedure was repeated for the reaction of $[Mn^{III}(OO^tBu)(^{6Me}dpaq)]^+$ with d_4-DHA, and no change in the decay rate of the Mn^{III}-alkylperoxo species was observed (Fig. S29).

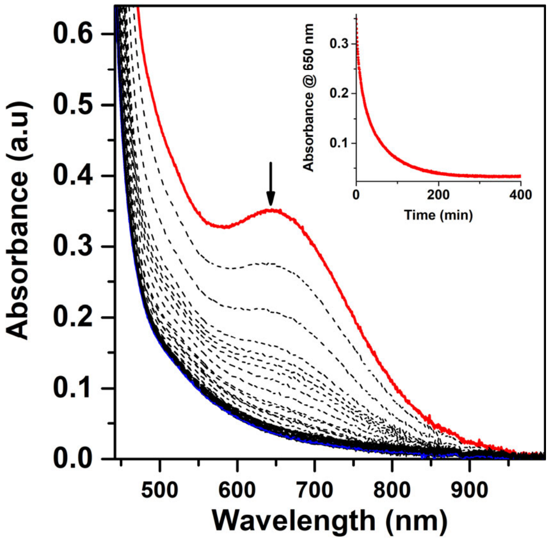

Fig. S28. Electronic absorption spectra monitoring the reaction of a 1.75 mM anaerobic solution of [Mn^{III}(OO^{t}Bu)(^{6Me}dpaq)]$^+$ (red trace) in CH_3CN with 100 equiv. of DHA at 323 K. The dashed traces show the reaction progress over time and the blue trace is the final product solution. Inset: time course for spectral changes at 650 nm.

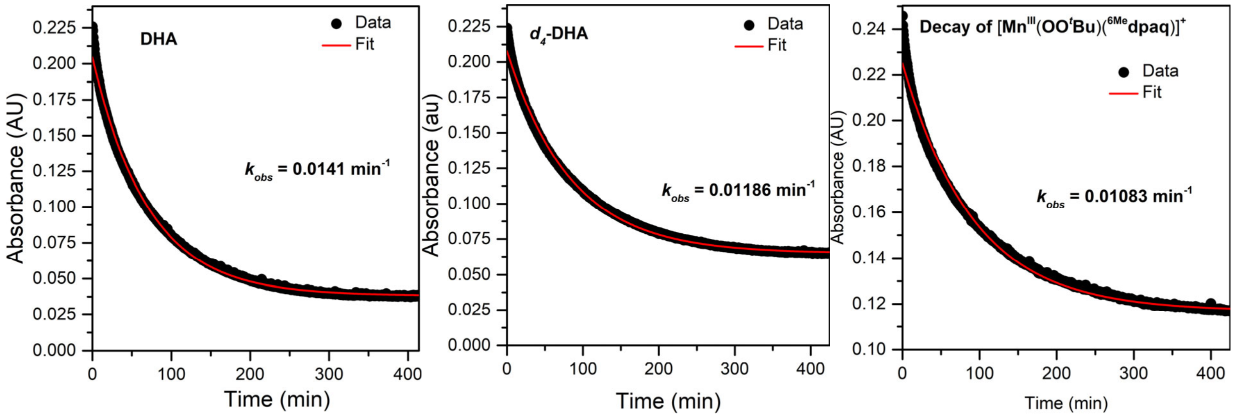

Fig. S29. Time trace for the reaction of 1.0 mM [Mn^{III}(OO^{t}Bu)(^{6Me}dpaq)]$^+$ with 100 equiv. DHA (left) d_4-DHA (middle) at 323 K and the decay of [Mn^{III}(OO^{t}Bu)(^{6Me}dpaq)]$^+$ in CH_3CN at 323 K.

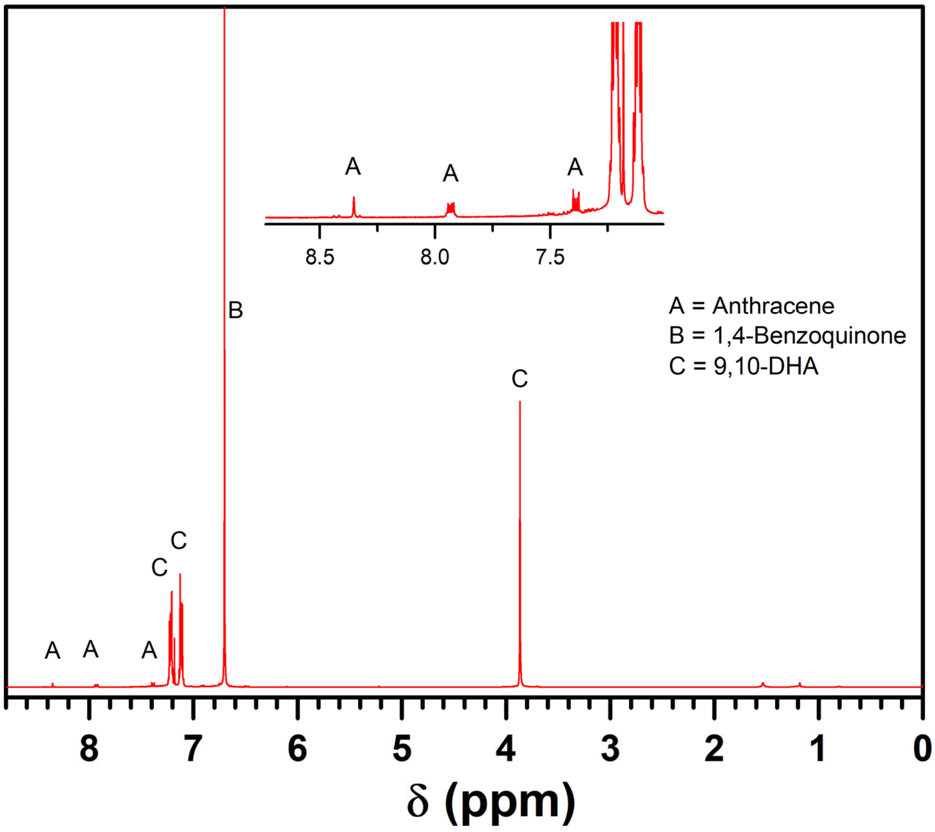

Fig. S30. ^{1}H-NMR spectrum of the organic product resulting from the reaction of 100 equiv. DHA with 1.75 mM [Mn^{III}(OO^{t}Bu)(^{6Me}dpaq)]$^+$ in CDCl$_3$ at 298 K under anaerobic conditions. Quantification of products was obtained using 1,4-benzoquinone (10 molar equiv. relative to starting Mn^{II} complex) as an internal standard.

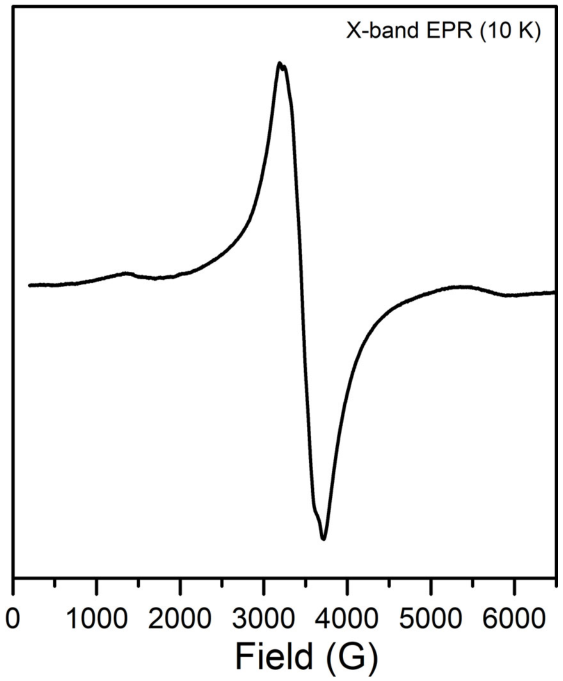

Fig. S31. 10 K, perpendicular-mode X-band EPR spectrum of the frozen CH_3CN solution following the reaction of 2 mM $[Mn^{III}(OO^tBu)(^{6Me}dpaq)]^+$ with 100 equiv. DHA at 298 K.

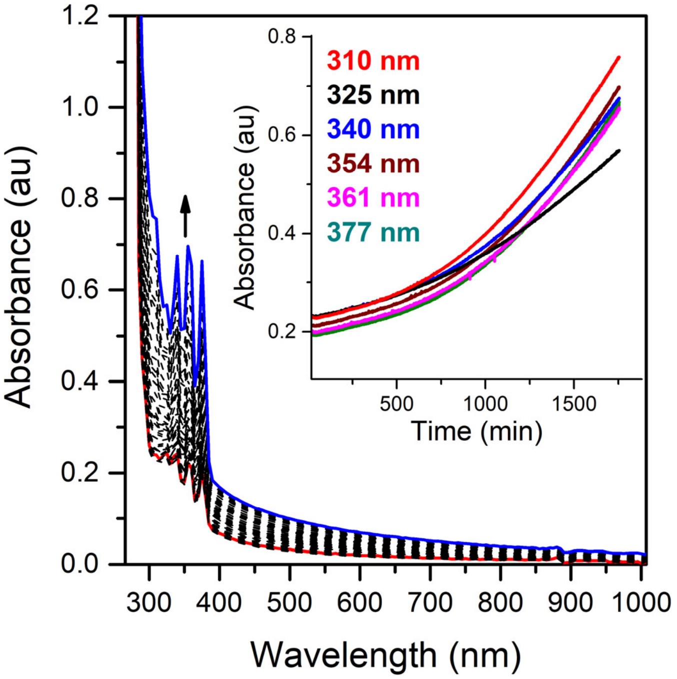

Fig. S32. Control experiments: DHA without $[Mn^{III}(OO^tBu)(^{6Me}dpaq)]^+$ at 323 K irradiated with UV light at 323 K under anaerobic condition for 33 hrs to investigate the contribution of Uv-radiation to the higher than expected conversion of DHA to anthracene.

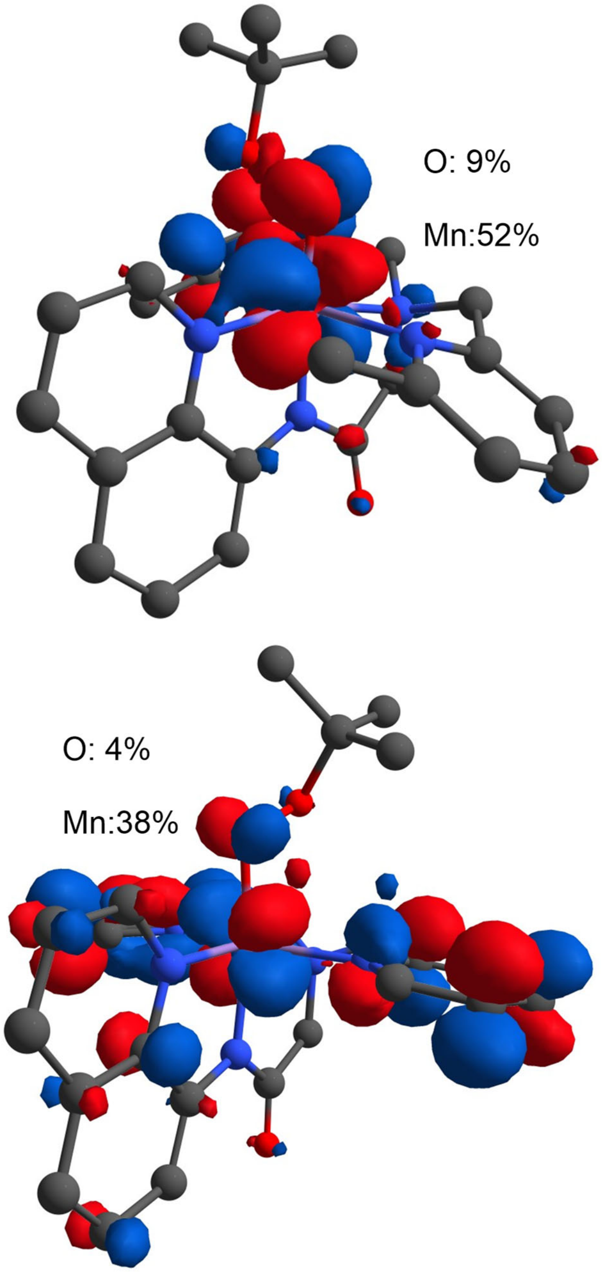

Fig. S33. Surface contour plots of the Mn^{III}-alkylperoxo π-antibonding MOs for $[Mn^{III}(OO^tBu)(^{6Me}dpaq)]^+$ (top) and $[Mn^{III}(OO^tBu)(dpaq)]^+$ (bottom) from DFT computations. Contributions to the MOs from the Mn and alkylperoxo O atoms are given as insets to the figures. The computations were performed using the *ORCA* 4.2.1 program, with the B3LYP functional with D3 corrections, def2-TZVP basis set for all atoms. The computations utilized the ZORA approximation and Grid5 and GridX5 parameters.

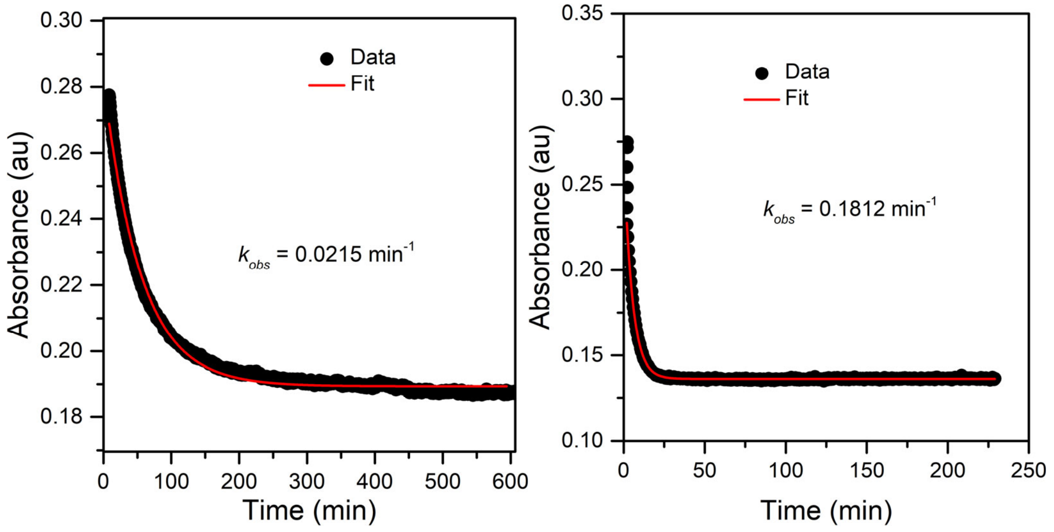

Fig. S34. Time traces for the thermal decay of 1.2 mM solutions of $[Mn^{III}(OOCm)(^{6Me}dpaq)]^+$ at 323 K in CH_3CN (left) and CD_3CN (right).

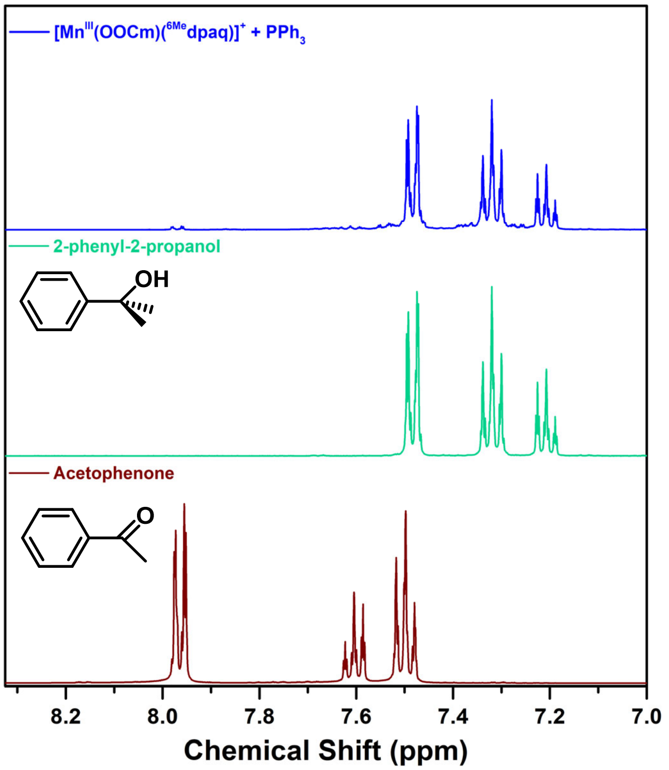

Fig. S35. ^1H NMR spectra of the organic products from the reaction of 22 mM $[Mn^{III}(OOCm)(^{6Me}dpaq)]^+$ with 5 equiv. of PPh_3 at 298 K (blue, top), and authentic samples of 2-phenyl-2-propanol (cyan, middle) and acetophenone (brown, bottom) in CD_3CN at 298 K.

Table S3. Crystal and refinement data for $[Mn^{II}(H_2O)(^{6Me}dpaq)](OTf)$, $[Mn^{III}(OH)(^{6Me}dpaq)](OTf)$, and $[Mn^{III}(OOCm)(^{6Me}dpaq)]$ (OTf).

Parameter	$[Mn^{II}(H_2O)(^{6Me}dpaq)](OTf)$	$[Mn^{III}(OH)(^{6Me}dpaq)](OTf)$	$[Mn^{III}(OOCm)(^{6Me}dpaq)](OTf)$
Formula	$C_{26}H_{26}F_3MnN_5O_5S$	$C_{26}H_{25}F_3MnN_5O_5S$	$C_{37}H_{38}F_3MnN_6O_6S$
Identification code	q66k	v60d	q05l
Formula weight	632.52	631.51	806.73
Crystal system	Orthothombic	Orthorhombic	Triclinic
Space group	$Pna2_1$	$Pna2_1$	P-1
Crystal size (mm^3)	0.109 x 0.082 x 0.025	0.100 x 0.050 x 0.030	0.140 x 0.020 x 0.010
a/Å	22.4917(5)	22.7367(9)	8.8894(4)
b/ Å	10.2847(2)	10.2260(4)	12.1129(5)
c/ Å	12.0025(3)	11.5888(4)	18.7531(9)
α/°	90.00	90.00	81.779(3)
β/°	90.00	90.00	82.588(3)
γ/°	90.00	90.00	72.703(3)
V/ $Å^3$	2776.42(11)	2694.46(18)	396.68(8)
Z	4	4	2
Dcalcd/g cm^{-3}	1.513	1.557	1.410
F(000)	1300	1296	836
μ(MoKα)/mm^{-1}	5.158	5.315	3.924
T/K	200(2)	200(2)	200(2)
λ/ Å	1.54178	1.54178	1.54178
θ range/°	3.931-70.211	3.888-68.257	2.390-70.328
Reflections collected	16163	9990	30272
Completeness to θ=66.000° (%)	96.7	99.5	96.0
Index ranges	$-26\leq h\leq 23$, $-12\leq k\leq 11$, $-11\leq l\leq 13$	$-26\leq h\leq 24$, $-12\leq k\leq 8$, $-13\leq l\leq 13$	$-10\leq h\leq 10$, $-14\leq k\leq 14$, $-22\leq l\leq 22$
Data/Restraint/parameters	4144 / 1 / 381	4121 / 1 / 378	6685 / 2 / 492
R(F), w $R_2(F^2)$ (> $2\sigma(F^2)$)	0.040, 0.0987	0.0603, 0.1696	0.0766, 0.2200
R(F), w $R_2(F^2)$ (all data)	0.0452, 0.1006	0.0659, 0.1767	0.0873, 0.2282
Absorption correction	Multi-scan	Multi-scan	Multi-scan
GOF on F^2	1.007	1.162	1.081
Largest peak/hole/ e$Å^{-3}$	0.864/-0.420	0.398/-0.752	1.001/-0.456
Max and min transmission	0.7533/0.4398	1.000/0.699	0.7533/0.5237
CCDC #	2048663	2049911	2048664

References

1. H. Nagao, N. Komeda, M. Mukaida, M. Suzuki and K. Tanaka, *Inorganic Chemistry*, 1996, **35**, 6809-6815.
2. V. R. Kokatnur and M. Jelling, *Journal of the American Chemical Society*, 1941, **63**, 1432-1433.
3. C. Walling and S. A. Buckler, *Journal of the American Chemical Society*, 1955, **77**, 6032-6038.
4. Y. Hitomi, K. Arakawa, T. Funabiki and M. Kodera, *Angew. Chem., Int. Ed.*, 2012, **51**, 3448-3452.
5. D. B. Rice, G. B. Wijeratne, A. D. Burr, J. D. Parham, V. W. Day and T. A. Jackson, *Inorganic Chemistry*, 2016, **55**, 8110-8120.
6. M. K. Coggins, S. Toledo, E. Shaffer, W. Kaminsky, J. Shearer and J. A. Kovacs, *Inorganic chemistry*, 2012, **51**, 6633-6644.
7. G. B. Wijeratne, B. Corzine, V. W. Day and T. A. Jackson, *Inorganic Chemistry*, 2014, **53**, 7622-7634.
8. D. B. Rice, A. Munasinghe, E. N. Grotemeyer, A. D. Burr, V. W. Day and T. A. Jackson, *Inorganic Chemistry*, 2019, **58**, 622-636.
9. Data Collection: SMART Software in APEX2 v2014.11-0 Suite. Bruker-AXS, 5465 E. Cheryl Parkway, Madison, WI 53711-5373 USA.
10. Data Reduction: SAINT Software in APEX2 v2014.11-0 Suite. Bruker-AXS, 5465 E. Cheryl Parkway, Madison, WI 53711-5373 USA.
11. Refinement: SHELXTL Software in APEX2 v2014.11-0 Suite. Bruker-AXS, 5465 E. Cheryl Parkway, Madison, WI 53711-5373 USA.
